# Wireless acoustic-driven current source achieving nanoampere-level at millimeter-scale

**DOI:** 10.1038/s41467-026-78004-y

**Published:** 2026-09-28

**Authors:** Yang Yuan, Shengkuo Zhang, Jikai Zhang, Zhuo Liu, Yuxiang Wang, Wanli Yang, Xi Chen, Yanghui Liu, Fa Chen, Kuan Ning, Kunkun Yan, Guangzu Zhang, Qiuyun Fu, Yongqing Fu, Tao Sun, Wei Luo

**Affiliations:** 1 School of Integrated Circuits, Huazhong University of Science and Technology, Wuhan, PR China; 2 School of Microelectronics, Jiangxi Normal University, Nanchang, PR China; 3 Jiangxi Provincial Key Laboratory of Advanced Electronic Materials and Devices, Jiangxi Normal University, Nanchang, PR China; 4 Naval University of Engineering, Wuhan, PR China; 5 Tongji Medical College, Huazhong University of Science and Technology, Wuhan, PR China; 6 School of Aerospace Engineering, Huazhong University of Science and Technology, Wuhan, PR China; 7 School of Engineering, Physics and Mathematics, Northumbria University at Newcastle, Newcastle upon Tyne, UK; 8 School of Instrument Science and Technology, Xi’an Jiaotong University, Xi’an, PR China; 9 State Key Laboratory of High End Heavy Load Robots, Foshan, PR China

**Keywords:** Electrical and electronic engineering, Electronic and spintronic devices

## Abstract

Piezoelectric surface acoustic wave devices can drive charge carriers in semiconductors through the acoustoelectric effect, generating direct currents with potential applications in sensing and biomedicine. However, current sources based on this mechanism that combine nanoampere-level precision, a wide dynamic range, and long-term operational stability have yet to be demonstrated. Here, we demonstrate an acoustically driven current source based on an InSb heterostructure integrated with a piezoelectric substrate. The output current is tunable from 1.22 nA to 25.46 μA by adjusting the input radio-frequency power, with a sensitivity down to the nanoampere scale. At an input power of 10 mW, the device delivers an average output current of 1.54 μA with 1% accuracy and maintains a standard deviation of 4.82 nA over 12 h of continuous operation. Its acoustic-to-electrical conversion efficiency can be further enhanced by implementing multiple interdigital transducer arrays and single-phase unidirectional transducer structures, enabling cooperative excitation and directional acoustic energy propagation. The device also supports wireless excitation and accelerates wound healing in a rat full-thickness skin defect model under low-intensity electrical stimulation. These results establish a wireless acoustoelectric platform for stable, tunable, and miniaturized current delivery, suggesting potential for biomedical applications.

## Abstract (web summary)

The work demonstrates a wireless, miniaturized current source using surface acoustic waves and a semiconductor heterostructure, achieving stable, tunable nanoampere to microampere currents with high accuracy and long-term stability, suggesting potential for biomedical applications.

## Introduction

Microcurrent stimulators have been widely employed for neural modulation, tissue regeneration, and pain therapy, where stable and low-amplitude currents are critical for ensuring therapeutic efficacy^1–4^. Similar requirements exist for sensor networks, which are commonly dependent on compact and remotely powered current sources to maintain basic operations or support intermittent activities^5^. More recently, the development of radio-frequency (RF) signal processor based on piezoelectric acoustic wave technologies has underscored the need for micro-scale and acoustically compatible power modules, which are capable of achieving reliable performance under highly demanded environmental conditions^6–8^. Miniaturized current sources with nanoampere-level capabilities have significant application potentials in various biomedical systems, sensor networks, and RF signal processing platforms.

Modern complementary metal oxide semiconductor (CMOS) technology can generate stable nanoampere-level currents^9–11^, typically relying on wired power delivery and thereby constraining device integration and system flexibility. Wireless power transfer (WPT) offers a viable pathway toward autonomous and untethered operation. In the conventional WPT systems, a stable direct current (DC) output is typically obtained through a cascade of functional stages, including an antenna for RF reception, impedance matching networks, nonlinear rectification elements, and filtering components. At sufficiently high RF input power levels, the conventional diode- or transistor-based rectification circuits can be substantially simplified, and complex impedance matching networks are often no longer required. Such rectifiers can therefore be realized in highly compact integrated forms using mature silicon technologies.

In this context, rather than pursuing conventional semiconductor-based RF-to-DC conversion technologies, we explore an alternative physical mechanism based on the acoustoelectric (AE) effect. In this approach, a continuous DC current is generated through the interaction between surface acoustic waves (SAWs) and semiconductors. This mechanism enables RF-to-DC conversion without relying on discrete semiconductor rectification elements, conceptually consolidating the energy conversion process within a single heterostructure platform. This enables a compact and battery-free solution capable of stable nanoampere-level output under wireless RF excitation, where the incident RF signal is coupled via an antenna to directly drive the device.

The electric field accompanying the SAWs propagating on a piezoelectric substrate can be applied for manipulating and transporting carriers, generating an AE current. This phenomenon has been confirmed in high-mobility materials such as graphene and two-dimensional electron gas (2DEG)^12–14^. Nonetheless, preparation of such the acoustic-driven current sources encounters several difficulties, mainly including the following two aspects, i.e., (1) difficulties for integrating high-quality semiconductor materials with strongly piezoelectric materials such as LiNbO_3_^15,16^; and (2) weak interactions between SAW and semiconducting carriers^17–19^.

Recent advancements in AE devices have explored suspended thin-film architectures, such as lithium niobate-on-silicon waveguides supporting Lamb waves, which achieve DC yields by strictly confining acoustic energy within the membrane^20^. However, fabrication of such suspended structures typically involves complex wafer bonding and cavity release processes, significantly increasing manufacturing costs and integration complexity. Developing AE current sources based on simpler and monolithically grown heterostructures on bulk piezoelectric substrates remains highly desirable, despite the inherent challenges of material integration. For fabricating high-speed and low-power electronic devices, InSb stands out as a material with considerable merits, attributed to its high electron mobility^21,22^. However, the lattice mismatch between InSb and LiNbO_3_, coupled with oxygen diffusion from the LiNbO_3_ substrate into InSb, makes the deposition of InSb on LiNbO_3_ substrates a challenging task. Previous studies revealed that introducing a dielectric layer, SiO_*x*_, between the LiNbO_3_ substrate and InSb film effectively suppressed degradation of crystal quality caused by oxygen diffusion from the LiNbO_3_ substrate^23^. Nonetheless, this SiO_*x*_ dielectric layer failed to improve the crystalline structure of the InSb film, consequently severely affecting its electrical properties.

Herein, we report a methodology using semiconductor heterostructures to enhance the interaction between SAWs and semiconducting carriers, thereby achieving significant improvements in the AE conversion efficiency. The proposed acoustic-driven current source is based on a heterostructure comprising a SiO_*x*_ protective layer, an InSb active layer with a high carrier mobility and a low carrier density, an In_0.6_Al_0.4_Sb buffer layer, and a 128°YX LiNbO_3_ substrate. The fundamental structure of such acoustic-driven current sources is a SAW delay line with a resonant frequency of 434.75 MHz, where the SiO_*x*_/InSb/In_0.6_Al_0.4_Sb film is precisely deposited in the acoustic wave propagation region. Utilizing a coupling of modes (COM) model, which includes the AE effect, we compute the transmission coefficient *S*_21_ for the acoustic-driven current source, accurately predicting the output current values. The device’s performance has been further enhanced through an optimized design based on the model. The acoustic-driven current source demonstrates a wide dynamic range, high output stability, and precise tunability. In addition, wireless operation and in vivo demonstrations highlight the device’s potential for future biomedical electronic applications.

## Results

### Material platform overview

**Fig. 1:**
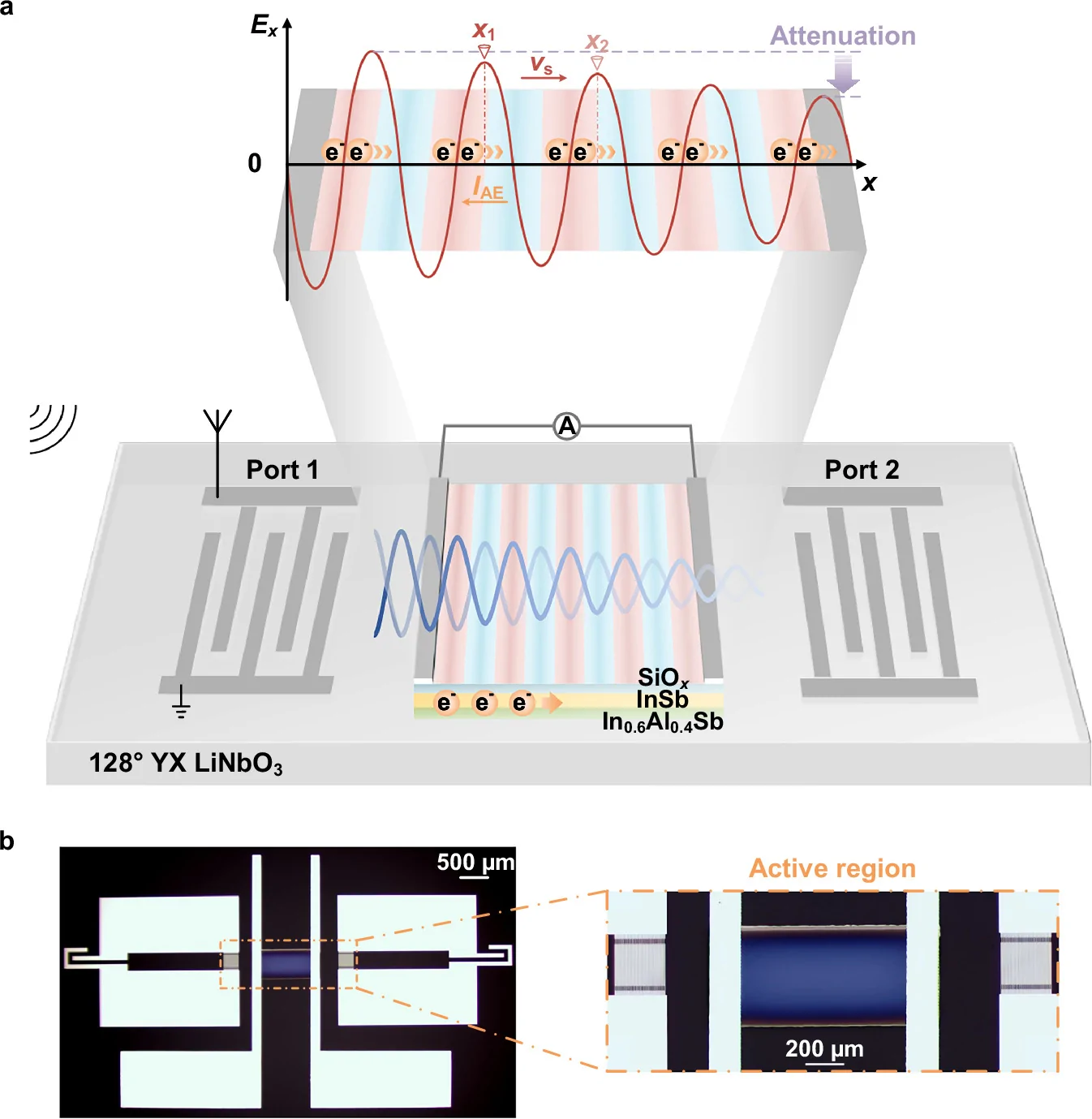
Operating mechanism and device structure of the acoustic-driven current source. **a** Schematic illustration of the AE current generation and wireless excitation mechanism. The dark gray regions represent the Al IDTs and Ag contact electrodes. In the AE film region, the alternating red and blue areas represent the periodic potential field associated with the SAW propagating along the *x*-axis. *E*_*x*_ denotes the electric field along the *x*-direction, while *x*_1_ and *x*_2_ mark the locations of two adjacent electric field peaks. *v*_s_ denotes the SAW velocity, e^-^ denotes electrons transported by the SAW, and *I*_AE_ represents the AE current. **b** Optical micrograph of the fabricated acoustic-driven current source, whose miniaturized active region measures approximately 2.3 mm × 0.5 mm. Source data are provided as a Source Data file.

A schematic illustration of an acoustic-driven current source with the implemented AE effect is shown in Fig. 1a. The wireless acoustic-driven current source receives the RF signals through antennas, which excite Rayleigh waves propagating from the interdigitated transducers (IDTs) along the X-axis of the LiNbO_3_ substrate. When the acoustic wave propagates as elastic vibrations on the surface of the piezoelectric substrate, the resulting strain field induces polarization of the substrate materials, establishing a mobile piezoelectric potential field. The transient electric field associated with the acoustic waves facilitates charge accumulation, generating measurable AE currents within the film through the interaction between phonons and electrons. The AE current ${I}_{{\mbox{AE}}}$ is expressed as follows^24^,

1 $${I}_{{{{\rm{AE}}}}}=\frac{\mu {Pf}}{{{v}_{{{\rm{S}}}}}^{2}}{K}_{{{{\rm{eff}}}}}^{2}\pi \frac{{\sigma }_{{{{\rm{S}}}}}/{\sigma }_{{{{\rm{M}}}}}}{1+{\left({\sigma }_{{{{\rm{S}}}}}/{\sigma }_{{{{\rm{M}}}}}\right)}^{2}}$$

where μ is the mobility of the AE film, P represents the power of the SAW at the center of the film, calculated from the input RF power and the transmission coefficient *S*_21_ of the device. The SAW frequency is defined as $f={v}_{{\mbox{S}}}/\lambda$, where *v*_S_ and λ denote the acoustic velocity and wavelength, respectively. ${K}_{{{{\rm{eff}}}}}^{2}$ denotes the electromechanical coupling coefficient. The sheet conductivity *σ*_S_ of the heterostructure films is given by ${\sigma }_{{\mbox{S}}}={n}_{{\mbox{S}}}{e}_{0}\mu$, where ${n}_{{\mbox{S}}}$ is the sheet carrier density, and ${e}_{0}$ is the elementary charge. The characteristic sheet conductivity ${\sigma }_{{\mbox{M}}}$ is given by ${\sigma }_{{\mbox{M}}}={v}_{{\mbox{S}}}({\varepsilon }_{0}+\varepsilon )$, where$\,{\varepsilon }_{0}$ is the vacuum dielectric constant, and ε is the dielectric constant of the piezoelectric medium.

According to Eq. (1), the AE current in the device is primarily governed by three key factors. (1) The piezoelectric substrate. The AE current is directly proportional to the ${K}_{{{{\rm{eff}}}}}^{2}$ and inversely proportional to the *v*_S_. (2) The IDT structure. The AE current reaches its maximum value at the resonant frequency defined by the IDT period (i.e., wavelength *λ*), where the acoustic energy transferred to carriers is maximized. Notably, longer wavelengths enhance bulk wave penetration at the expense of surface energy density, thereby reducing the AE current amplitude. (3) The AE film. It is crucial to maximize the mobility μ of the semiconductor film. When ${{\upsigma}}_{{\mbox{S}}}={{\upsigma}}_{{\mbox{M}}}$, the semiconducting carriers acquire the maximum energy from the acoustic waves. To achieve this condition, it is crucial to ensure high mobility μ and low sheet conductivity ${\sigma }_{{\mbox{S}}}$ of the semiconductor film.

**Fig. 2:**
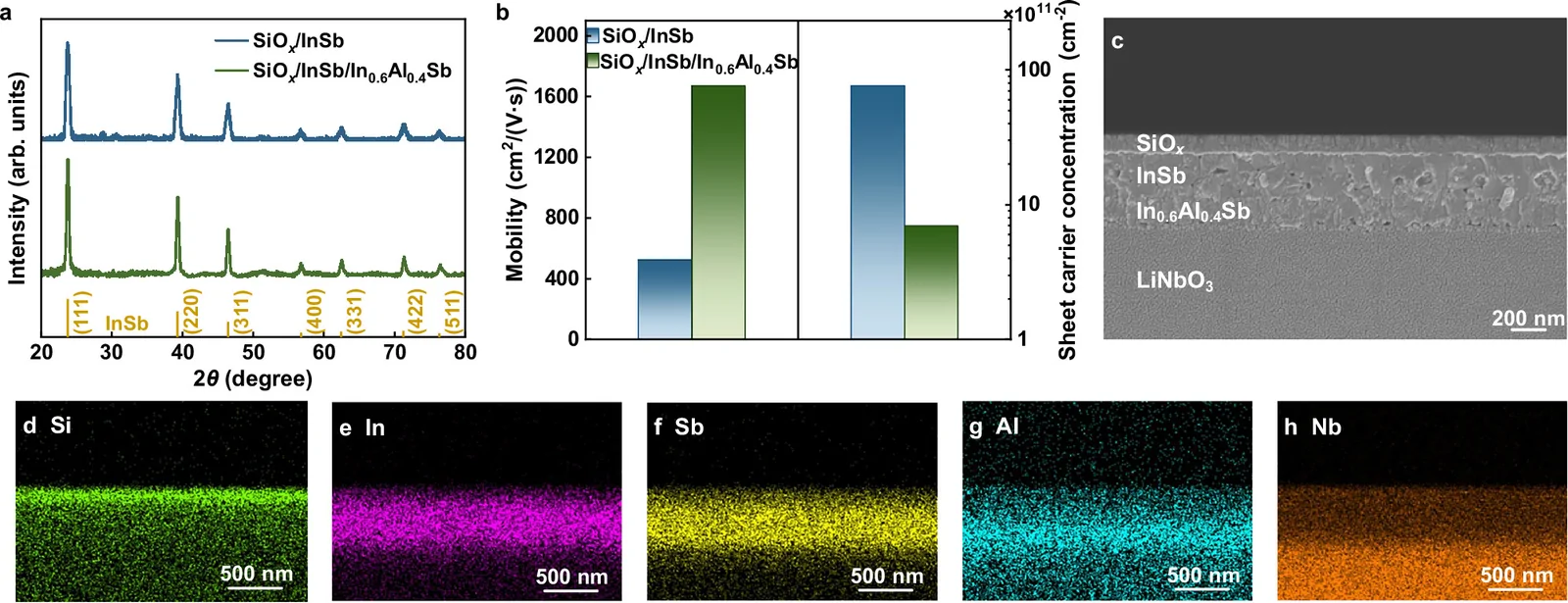
Structural and electrical characterization of SiO_*x*_/InSb/In_0.6_Al_0.4_Sb heterostructure films. **a** GIXRD patterns of the InSb layer, and (**b**) mobility and sheet carrier concentration of SiO_*x*_/InSb and SiO_*x*_/InSb/In_0.6_Al_0.4_Sb films. **c** SEM image, and (**d–h**) EDS mapping images of the SiO_*x*_/InSb/In_0.6_Al_0.4_Sb heterostructure films on a LiNbO_3_ substrate. Source data are provided as a Source Data file.

An optical micrograph of the fabricated acoustic-driven current source is shown in Fig. 1b. The device was constructed on a heterostructure platform that integrated an epitaxial InSb semiconductor film onto a 128°YX LiNbO_3_ substrate, where the InSb layer was deposited by magnetron sputtering. However, direct deposition of InSb on LiNbO_3_ led to severe lattice mismatch and oxygen diffusion from the substrate. To address these challenges, an In_0.6_Al_0.4_Sb buffer layer was introduced between the LiNbO_3_ and InSb layers, while a SiO_*x*_ capping layer was added on top of the InSb film to prevent degradation during annealing. The measurable AE current was generated exclusively in the InSb layer, where its high carrier mobility and suitable sheet carrier density allowed SAW-induced piezoelectric fields to efficiently drive electron transport, yielding the observed DC current. This SiO_*x*_/InSb/In_0.6_Al_0.4_Sb multilayer structure enabled precise control of both layer thickness and alloy composition, allowing optimization of the electrical performance of the InSb active layer and enhancing the coupling between charge carriers and SAWs. To further verify the structural quality and electrical properties of the designed multilayer platform, detailed material characterizations of the SiO_*x*_/InSb/In_0.6_Al_0.4_Sb heterostructure films were performed, as summarized in Fig. 2.

Crystalline structure of the InSb layer in the heterostructure films, with and without the In_0.6_Al_0.4_Sb buffer layer, was examined using grazing incidence X-ray diffraction (GIXRD, PANalytical B.V., X’Pert PRO) with Cu Kα radiation over a 2*θ* range of 15–85°. The incident angle was carefully adjusted to ensure that the diffraction signal originated predominantly from the InSb layer while minimizing contributions from the underlying layers and the LiNbO_3_ substrate. As shown in Fig. 2a, introducing the In_0.6_Al_0.4_Sb buffer layer significantly increases the diffraction peak intensity of InSb and narrows its full width at half maximum (FWHM), verifying the improved crystalline quality of the active layer. Additional analysis of the buffer layer’s influence on the structural properties of InSb is provided in Supplementary Note Ⅰ. As depicted in Fig. 2b, Hall measurements (ECOPIA, HMS-5500) revealed that the SiO_*x*_/InSb/In_0.6_Al_0.4_Sb heterostructure shows higher mobility and lower sheet carrier density than the SiO_*x*_/InSb heterostructure. Specifically, it achieves a mobility of 1667 cm^2^·V^−1^·s^−1^, a sheet carrier density of 6.94 × 10^11 ^cm^−2^, and a sheet conductivity of 1.9 × 10^−4^ S at room temperature. The InSb active layer is n-type, whereas the In_0.6_Al_0.4_Sb buffer layer exhibits p-type conductivity. A scanning electron microscope (SEM, ZEISS, GeminiSEM 300) image (Fig. 2c) clearly shows interfaces among SiO_*x*_, InSb, and In_0.6_Al_0.4_Sb layers. The InSb film thickness is approximately 250 nm, an optimized value identified through finite-element simulations to balance electrical performance with AE coupling efficiency (see Supplementary Note Ⅰ,Ⅰ). Energy-dispersive X-ray spectroscopy (EDS) mapping (Fig. 2d–h) further verifies the multilayer stacking sequence and the elemental distribution within each layer.

Benefiting from its crystallinity, high mobility, and optimized carrier density, the SiO_*x*_/InSb/In_0.6_Al_0.4_Sb heterostructure can be adopted as the material platform for the device, providing a high-quality active layer that enables efficient coupling between charge carriers and SAWs and forming the basis for subsequent evaluation of its performance.

### Frequency response and AE current

**Fig. 3:**
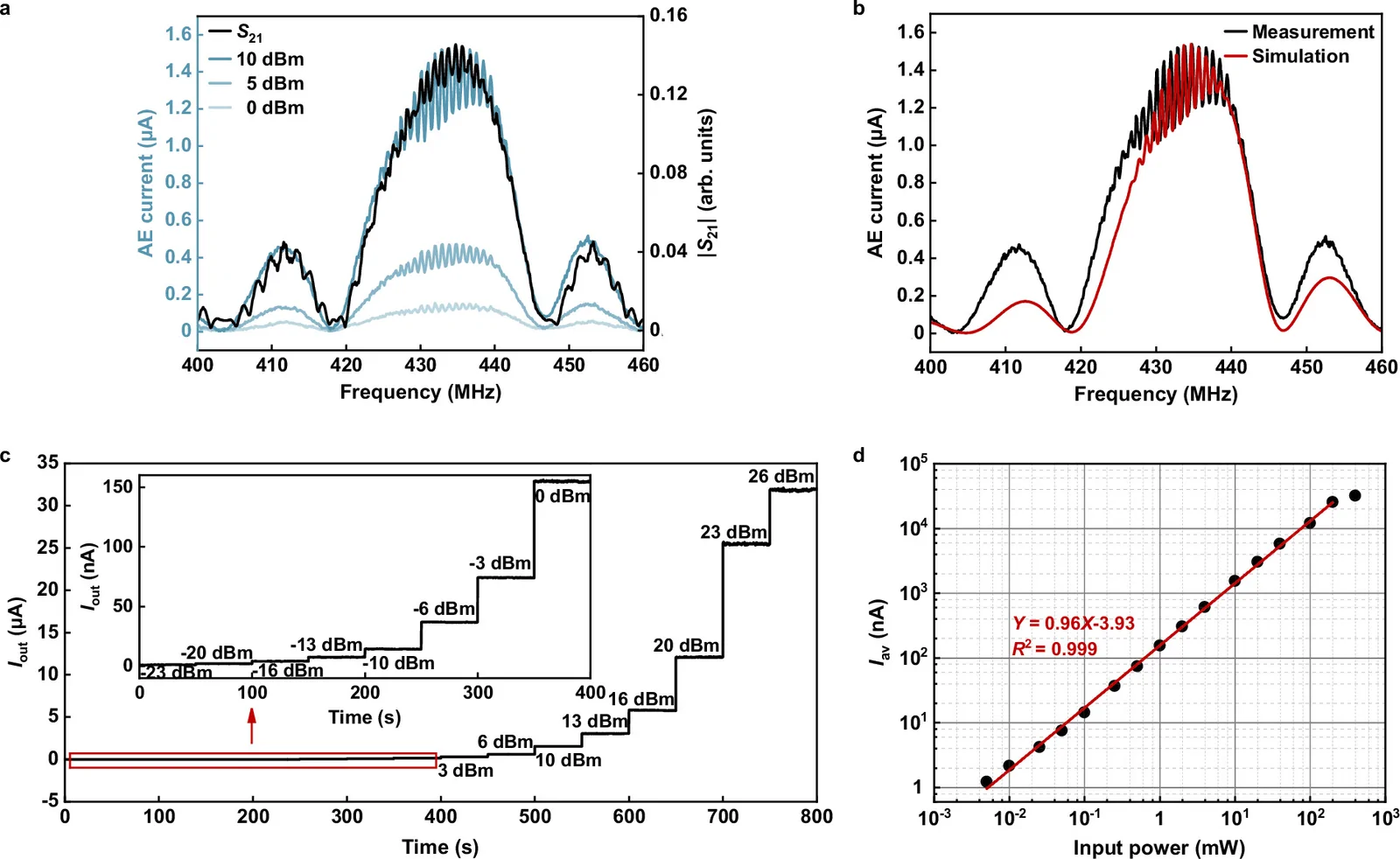
Frequency response and AE current characteristics of the SAW device. **a** Measured AE current (left axis) and *S*_21_ (right axis) versus frequency at different input RF powers. **b** The simulation and measurement of AE current as a function of frequency at an input RF power of 10 dBm. **c** Current responses and (**d**) average current versus input RF power, both measured at the device center frequency of 434.75 MHz. Source data are provided as a Source Data file.

The AE effect converts the applied RF signal into a DC current in the semiconductor film. Current characteristics of the acoustic-driven current source are shown in Fig. 3. In Fig. 3a, a frequency sweeping signal (400–460 MHz) with various input powers (0 dBm, 5 dBm, 10 dBm) was applied to the IDTs. The AE current, which is a DC current output generated from the input RF signal via the AE effect, was then measured without apply any DC bias. The contour of the AE currents closely resembled the measured transmission coefficient *S*_21_, demonstrating a significant correlation between them (see the Supplementary Note Ⅰ,Ⅰ,Ⅰ for frequency response characteristics of the device). The passband ripples in *S*_21_ and AE current arise from triple transit, caused by multiple reflections of the acoustic wave at the output IDT, which modulate the acoustic wave amplitude and are mirrored in the AE current. The AE current exhibits a positive correlation with the acoustic wave intensity, where the acoustic wave intensity is influenced by the resonant frequency of the device and RF power. At the center frequency of 434.75 MHz, the acoustic wave intensity reaches its maximum, resulting in both the minimum insertion loss and peak value of AE currents. Furthermore, with the increase in input RF power, there is an increased transfer of energy from the acoustic waves into the carriers, leading to a proportional increase in the AE current at different frequencies. The theoretical AE current curve, calculated by Eq. (1), is shown in Fig. 3b. The power values of the SAWs at the center of the film at different frequencies were determined using the *S*_21_ values obtained from the COM model and the input RF powers. Theoretical prediction results are aligned well with the experimental results, indicating that the combination of the COM model and Eq. (1) accurately predicted the AE currents.

The dynamic range of the output current is a crucial parameter metric for evaluating the performance of the current source. The AE current is primarily affected by RF power and SAW frequency, with the power exhibiting a linear trend with the AE current. Therefore, for practical applications of the current source, the RF frequency can be maintained at the center frequency of the IDTs, enabling the modulation of AE currents by adjusting the input powers. The temperature and relative humidity level during the current tests were maintained at 25 °C and 50%, respectively. As shown in Fig. 3c, continuous RF signals, with a frequency equal to the device’s center frequency, were applied to measure the currents at different RF powers. As the power was increased, the output currents were gradually increased. According to Fig. 3c, the average current ${I}_{{\mbox{av}}}$ was calculated, and the power units were converted to establish the functional relationship between the average current and input power as shown in Fig. 3d. The fitting results for the average currents in the logarithmic coordinate system across the range of input RF power from 5 μW to 200 mW showed a linear relationship with a correlation coefficient *R*^2^ of 0.999. The critical current value of the linear region was identified as the operational range for the current source, ranging from 1.22 nA to 25.46 μA and spanning four orders of magnitude. The corresponding output voltage, measured under the same conditions as the AE current, reached approximately 335 mV (see Supplementary Note IV), defining the maximum voltage for reliable operation with external loads.

The lower limit of the output current was primarily dependent on the RF power required to generate SAWs and the noise in the current. In cases of low powers, there were insufficient excitation of SAWs, and the accompanying electric field of the SAWs was quite weak. When the interactions between phonons and electrons were weak, the energy acquired by the carriers was insufficient to generate effective directional movement, leading to weak or insignificant output currents. With the increase in output current, the primary factor affecting the linearity of the curve was the Joule heating generated in the semiconducting layer. Under the constantly applied RF signals, the center frequency of the IDT was shifted due to the influences of temperature coefficients of frequency for both the film and substrate, leading to a decrease in the linearity of the output current curves. To further evaluate the effect of input power on the device temperature, a steady-state thermal simulation was performed (see the Supplementary Note V for details of the power-dependent steady-state temperature distribution in the acoustic-driven current source). Severe thermal dissipation resulted in damage to the device, such as dielectric breakdown in the film and damage of electrodes. To mitigate such types of Joule heating, we can increase the thermal conductivity of the substrate, such as using LiNbO_3_ thin film on Si instead of a bulk LiNbO_3_ piezoelectric to enhance heat dissipation efficiency^8^.

### Electrical performance of the current source

**Fig. 4:**
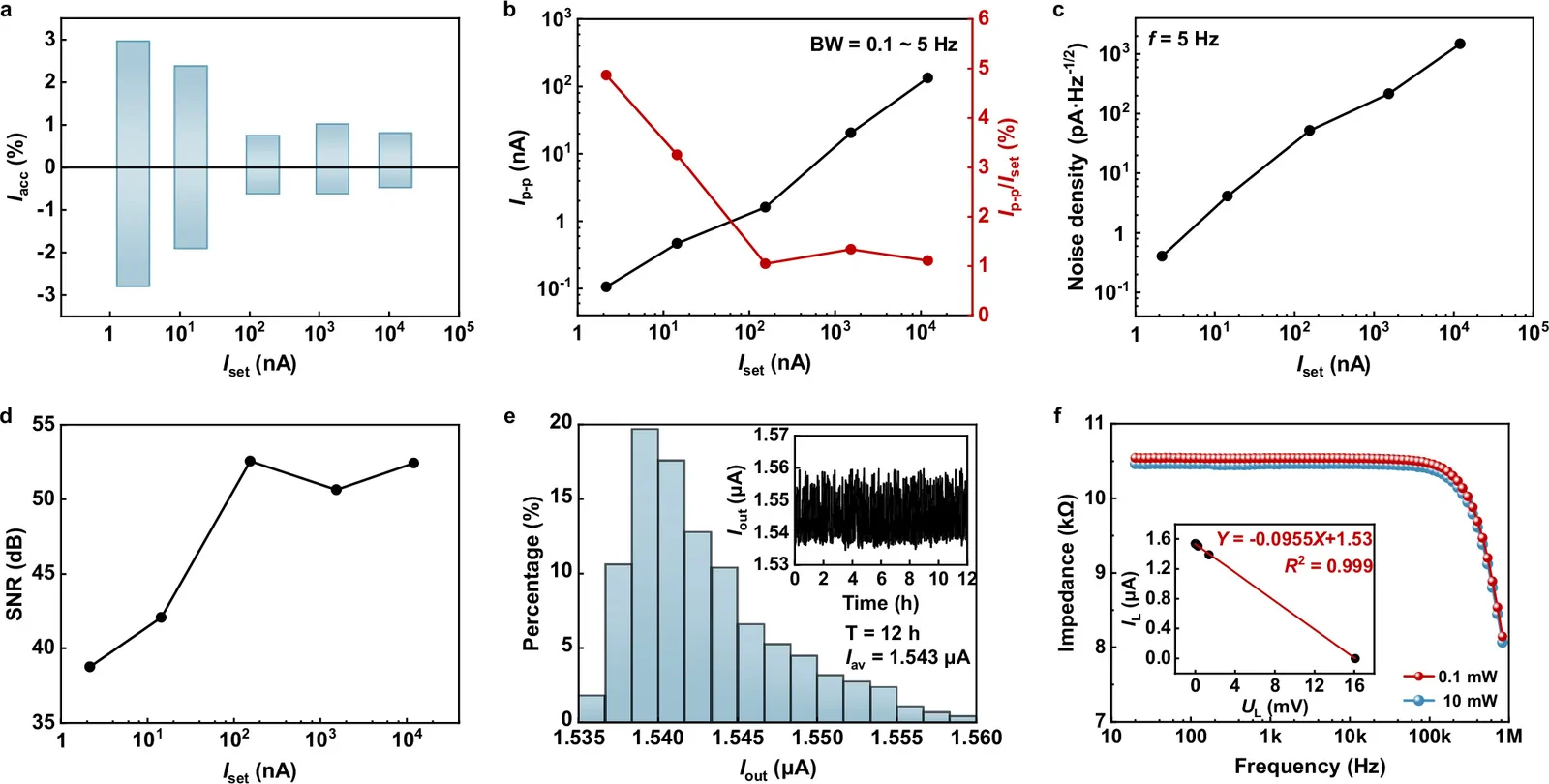
Stability, noise, and impedance of the acoustic-driven current source. At different input RF powers (0.01, 0.1, 1, 10, and 100 mW), (**a**) current accuracy, (**b**) ${I}_{{\mbox{p}}-{\mbox{p}}}$ and ${I}_{{\mbox{p}}-{\mbox{p}}}/{I}_{{\mbox{set}}}$ in the frequency range from 0.1 Hz to 5 Hz, **c** current noise density at 5 Hz, and (**d**) SNR of current noise density. **e** The continuous 12 h operation output current at an RF power of 10 mW. **f** The impedance characteristics of the current source at RF powers of 0.1 and 10 mW. Source data are provided as a Source Data file.

The stability of the output currents from the acoustic-driven current source was further investigated. The electrical characteristics of the current, with the input RF powers of 0.01, 0.1, 1, 10, and 100 mW, were analyzed over a sampling time of T = 50 s and a sampling rate ${F}_{s}=1{{{\rm{Hz}}}}$. The average current ${I}_{{\mbox{av}}}$ was considered as the set value ${I}_{{\mbox{set}}}$ for the current source, and the current accuracy ${I}_{{\mbox{acc}}}$, given by ${I}_{{\mbox{acc}}}=(I-{I}_{{\mbox{av}}})/{I}_{{\mbox{av}}}\times 100\%$, was employed to assess the discrepancies between the output current I and ${I}_{{\mbox{set}}}$. As shown in Fig. 4a, the current accuracy was maintained below 3% and exhibited an overall decreasing trend with increasing ${I}_{{\mbox{set}}}$, reaching a local minimum at *I*_set_ = 154.71 nA. The adjustable current source chip of LM134/LM234 from Texas Instruments demonstrated a maximum current accuracy of 8% when 2 μA ≤ *I*_set_ < 10 μA. In contrast, our developed acoustically-driven current source showed a higher current accuracy. We also evaluated the stability performance of the output currents in the frequency range from 0.1 Hz to 5 Hz, and the current was transformed from the time domain to the frequency domain through a fast Fourier transform. In the frequency domain, according to the Nyquist-Shannon sampling theorem, the maximum analyzable current frequency *f*_max_ is given by ${f}_{\max }={F}_{{\mbox{s}}}/2=6{{{\rm{Hz}}}}$. Due to the constraints of the sampling time T, the minimum analyzable current frequency ${f}_{\min }$ is given by ${f}_{\min }=1/T=0.02{{{\rm{Hz}}}}$. Utilizing a digital low-pass filter, the time domain peak-to-peak current ${I}_{{\mbox{p}}-{\mbox{p}}}$ and ${I}_{{\mbox{p}}-{\mbox{p}}}/{I}_{{\mbox{set}}}$ in the frequency range 0.1 Hz to 5 Hz was obtained, and the data are shown in Fig. 4b. With the increase of power, the value of ${I}_{{\mbox{p}}-{\mbox{p}}}/{I}_{{\mbox{set}}}$ was decreased from around 5% to 1.2%, even though the ${I}_{{\mbox{p}}-{\mbox{p}}}$ value exhibited an increment trend. Furthermore, when the value of ${I}_{{\mbox{set}}}$ exceeded 100 nA, the value of ${I}_{{\mbox{p}}-{\mbox{p}}}/{I}_{{\mbox{set}}}$ fluctuated around 1.2%, approaching its stability limit.

In practical applications, noise is a crucial metric for assessing the current’s stability. Therefore, the current noise density of the device was commonly investigated. As shown in Fig. 4c, the noise density at 5 Hz was selected as the representative low-frequency reference for quantitative comparisons, as it minimized instrument noise while capturing current fluctuations. The full noise spectrum across the measurable frequency range is provided in Supplementary Note VI. As the frequency was increased, there was a consistently decreasing trend in the current noise density under different input powers. Analysis of the noise density variations at 5 Hz at different set currents revealed that the current source exhibited the higher noise densities at higher output currents, possibly due to the introduction of additional nonlinear effects or thermal noises. To quantify the relationship between the current and noise, we calculated the signal-to-noise ratio (SNR), defined as ${\mbox{SNR}}=20{{\mathrm{lg}}}({I}_{{\mbox{set}}}/{\mbox{RMS}}(I-{I}_{{\mbox{set}}}))$. Figure 4d shows that the value of SNR increases with the increase of current and eventually becomes stabilized, and a higher SNR indicates a better current quality. Table 1 summarizes the electrical characteristics of the acoustic-driven current source, obtained from Fig. 4a–d, when the input RF power is 10 mW.

**Table 1:** The electrical characteristics of the acoustic-driven current source at *P*_*in*_ = 10 mW, *T* = 25 °C

Parameter	Test conditions	Value	Unit
Average current *I*_av_		1.54	μA
Current accuracy *I*_acc_		±1	%
Peak-to-peak current *I*_p-p_	BW = 0.1 ~ 5 Hz	20.58	nA
Current noise density	*f* = 5 Hz	214.23	${\mbox{pA}}$ Hz^-1/2^
SNR		50.64	dB

The continuous operation performance of the current source was further evaluated by conducting a continuous 12 h test at a temperature of 25 °C, with an input RF signal power of 10 mW. As shown in Fig. 4e, the obtained currents, i.e., *I*_min_ = 1.535 μA, *I*_max_ = 1.560 μA, *I*_med_ = 1.542 μA, are closely approximating *I*_av_ = 1.543 μA. The current was concentrated around *I*_av_, with a low standard deviation (σ = 4.82 nA), demonstrating its minimal fluctuations. These results clearly show that the current source demonstrates its good reliability and stability.

For the current source, it is desirable to maximize the output impedance to ensure that the influences of the load impedance on it can be negligible. In this study, the impedance characteristics of the current source were measured using an impedance analyzer (Wayne Kerr, 6500B) at RF powers of 0.1 and 10 mW, and the data are shown in Fig. 4f. At frequencies ranged from 20 Hz to 100 kHz, the impedance is approximately 10.5 kΩ. This value was further confirmed by direct I–V measurements under varying load resistances (Supplementary Note VII). The current source shows a capacitive characteristic, resulting in a decrease in impedance as the frequency exceeds 100 kHz. This RC roll-off behavior, with a corner frequency around 100 kHz, originates from the intrinsic capacitance of the IDT structure and the semiconductor heterojunction, in parallel with the output resistance. The variations in output impedances of the current source were negligible under different input powers. With an increase in input power, there is a slight reduction in the impedance due to the influence of Joule heating. Overall, while the output impedance of the acoustic-driven current source is lower than that of the conventional voltage-controlled current sources, it remains sufficiently high for intended low-power wireless applications, particularly when the load resistance is much smaller than the source impedance (e.g., < 1 kΩ). This ensures that current errors due to loading remain below 10%.

The output impedance is primarily determined by the nature of the AE film. Equation (1) shows that the semiconducting thin film carriers obtain its maximum energy when ${\sigma }_{{\mbox{S}}}={\sigma }_{{\mbox{M}}}=2.04\times {10}^{-6}$ S. The heterojunction film fabricated in this investigation exhibits a sheet conductivity of *σ*_S_ = 1.9 × 10^−4^ S. Despite attempts to further refine the film’s preparation process and decrease the sheet conductivity for increasing impedance, the potential increase in impedance is still constrained by the characteristic sheet conductivity ${{\upsigma}}_{{\mbox{M}}}$ of the substrate. Nevertheless, the developed acoustic-driven current source exhibits smaller output current and smaller volume compared to those of the voltage-controlled current source. With further optimization of film fabrication and device structure, the impedance output may be further enhanced, potentially extending the applicability of the device to higher-precision measurement scenarios. Within the temperature range between −20 and 40 °C, the frequency and current of the current source exhibited a linear relationship with temperature, demonstrating its predictability and repeatability (Supplementary Note VIII). Therefore, temperature compensation techniques can be easily employed to mitigate the temperature effects, ensuring a stable operation of such a current source.

### Wireless acoustic-driven current source

Most microcurrent sources have required wired connections to the power sources, increasing the size of the current source and limiting its application in confined or enclosed spaces. The wireless characteristics of SAW devices make the acoustic-driven current source suitable for scenarios which require miniaturized devices and wireless power supply, thereby reducing the needs for power maintenance and battery replacements^25,26^.

**Fig. 5:**
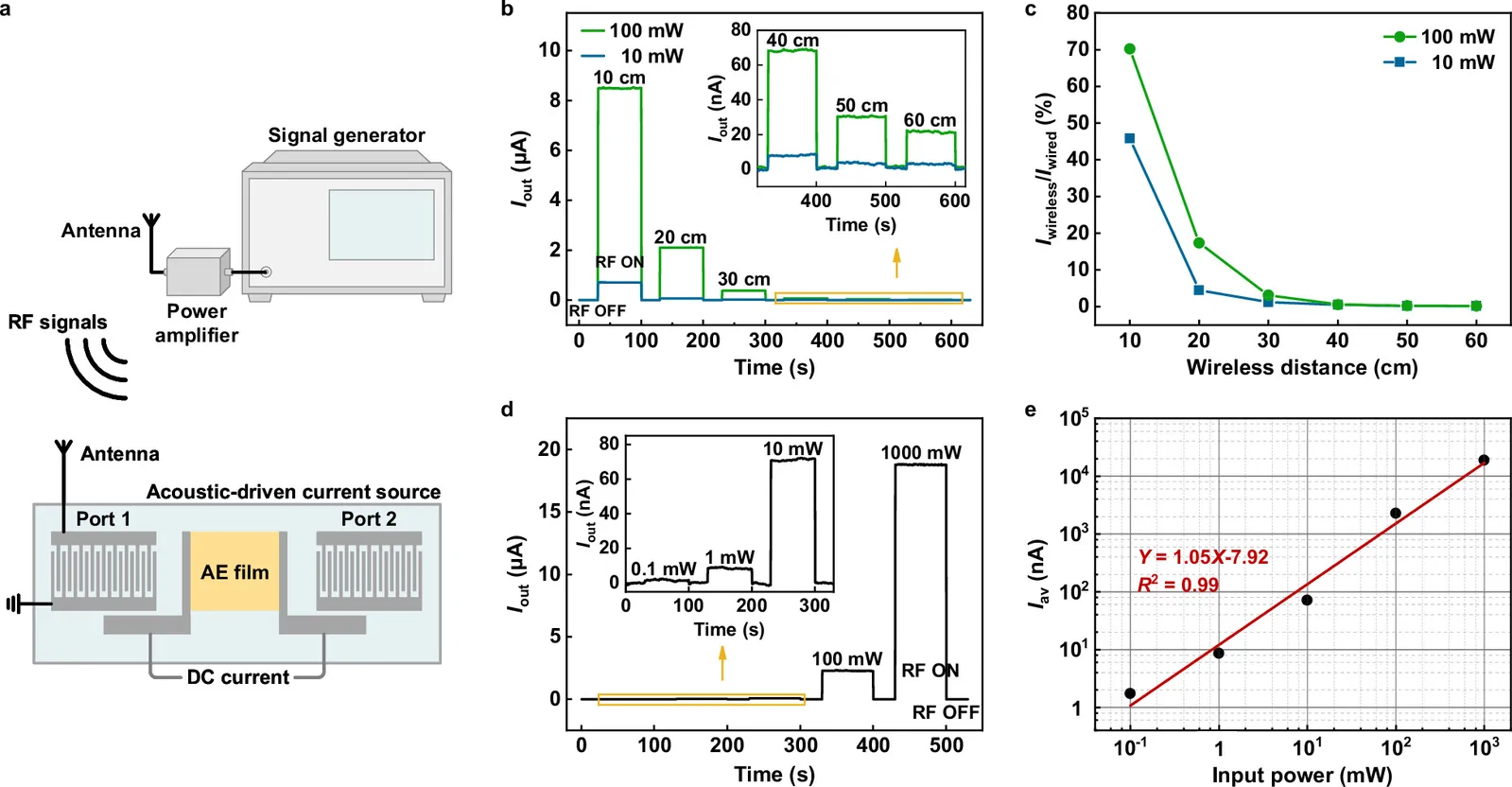
Wireless operation of the acoustic-driven current source. **a** Schematic illustration of the wireless acoustic-driven current source. In this device, the light blue region represents the 128° YX LiNbO_3_ substrate, and the dark gray regions represent the Al IDTs and Ag contact electrodes. **b** Current as a function of wireless transmission distance at RF powers of 10 mW and 100 mW. **c** Ratio of wireless to wired current (*I*_wireless_/*I*_wired_) at different wireless transmission distances. At a wireless transmission distance of 20 cm, (**d**) current as a function of RF power and (**e**) fitted curve of average current versus input RF power. Source data are provided as a Source Data file.

The acoustic-driven current source can receive the RF signals through antennas and converts them into SAWs, utilizing the AE effect to generate output currents, as shown in Fig. 5a. To verify this, we measured the current responses at different wireless transmission distances, with the RF signal frequency set as the center frequency of IDTs and the powers set at 10 mW and 100 mW, as shown in Fig. 5b. Stable current outputs were obtained at distances shorter than 60 cm. Considering that most implantable biomedical systems, such as neural stimulators and wireless therapeutic devices, are typically implanted just a few millimeters beneath the skin and require transmission distances of several tens of centimeters, the demonstrated transmission range of the acoustic-driven current source sufficiently meets the practical demands of these applications^27^. With the use of coaxial cables, average currents at the powers of 10 mW and 100 mW were obtained as 1.54 μA and 12.08 μA, respectively, providing a baseline for evaluating the effect of wireless transmission distance on the ratio of the wireless current to the wired current. As the wireless transmission distance was further increased, the RF power loss increased, and the corresponding current ratio decreased. When the distance was increased to 30 cm, the wireless current dropped to less than 5% of the wired baseline, as shown in Fig. 5c. At the same wireless transmission distance, the current with an input RF power of 100 mW exhibited a higher current ratio compared to that with the power of 10 mW. This is attributed to the larger power signals, which can maintain higher energy over a shorter distance, effectively overcoming signal loss in the propagation.

We further measured the current responses to RF power ranging from 0.1 to 1000 mW with the wireless transmission distance set at 20 cm, and the obtained results are shown in Fig. 5d. At an RF power of 0.1 mW, the current source reached the lower limit of the output current for that transmission distance. The fitting curve between average current and power, shown in Fig. 5e, exhibits a notable linearity, allowing for precisely controlled output current by modulating the input RF power. While this demonstrates the effectiveness of the acoustic-driven current source for wireless power delivery, its linearity in practical scenarios may be influenced by external factors such as antenna orientation, multi-path fading, and environmental reflections, which can cause non-linear attenuation of the received RF power. Future work will be focused on optimizing antenna design and incorporating impedance matching networks to enhance the robustness of the power transfer and maintain linearity across a wider range of practical deployment conditions.

Compared with the conventional WPT technologies, such as inductive^28,29^, magnetoelectric^30^, and optical coupling^31^, the proposed acoustic-driven current source introduces a fundamentally different mechanism based on the AE effect. In this approach, SAWs directly drive charge carriers within a semiconductor layer to generate the direct current, without requiring rectification circuits. This not only simplifies the device structure but also facilitates integration, offering new possibilities for miniaturized and multifunctional wireless systems.

### Different excitation IDT schemes

To enhance the output current in wireless operation, we identified and addressed two key limitations of the conventional delay line structure. The first one is acoustic wave reflection at the output IDT, which generates a counterproductive reverse current. The second one is the bidirectional propagation of acoustic waves excited by the input IDT, which results in half of the energy loss.

**Fig. 6:**
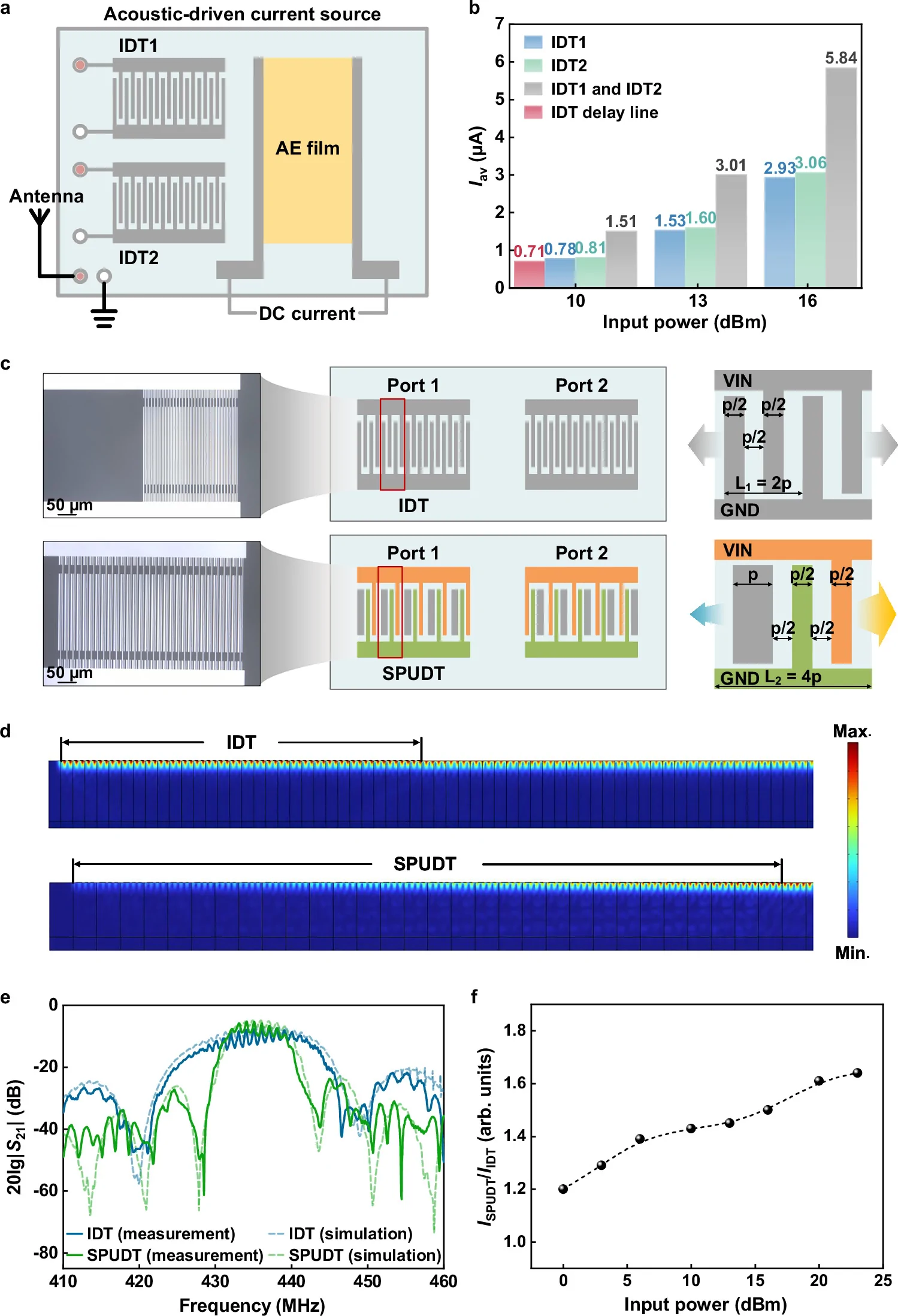
Wireless performance of acoustic-driven current sources with different excitation IDT schemes. **a** Schematic diagram of the acoustic-driven current source incorporating a multi-IDT array, where the light blue region represents the 128° YX LiNbO_3_ substrate, and the dark gray regions represent the Al IDTs and Ag contact electrodes. **b** Linear superposition characteristic of the total *I*_av_ (10/13/16 dBm, wireless transmission distance of 10 cm), where the red bar represents the *I*_av_ of IDT delay line operating, the blue and green bars represent the *I*_av_ of IDT1 and IDT2 operating independently, respectively, and the gray bars denote the *I*_av_ when IDT1 and IDT2 work simultaneously. **c** Schematic diagrams and optical micrographs of the IDT and SPUDT delay lines. **d** Displacement field simulations of the IDT and SPUDT. **e** Measured (solid lines) and simulated (dashed lines) frequency response characteristics of the IDT (blue) and SPUDT (green) delay lines without the AE film. **f** Output current ratio (*I*_SPUDT_/*I*_IDT_) of the two types of delay line-based devices across a range of input powers (0–23 dBm, wireless transmission distance of 10 cm). Source data are provided as a Source Data file.

By disabling the output IDT and operating the device with only a single input IDT, we observed an enhanced output current compared with the configuration containing both input and output IDTs, indicating that single-port excitation is more suitable for wireless current generation. Based on this finding, we developed a modified IDT architecture to further improve performance, namely a multi-IDT array for current enhancement. The multi-IDT array employed two identical input IDTs (IDT1 and IDT2) operating on an expanded AE film. As shown in Fig. 6a, these IDTs had structural parameters fully consistent with those previously detailed and used the same antenna configuration for wireless excitation. When activated simultaneously under the same RF input power, their combined output currents closely matched the linear sum of their individual outputs (Fig. 6b), demonstrating effective current superposition and scalable design.

In parallel, we designed a single-phase unidirectional transducer (SPUDT) structure to suppress bidirectional acoustic loss as shown in Fig. 6c. Its asymmetric electrode layout confines most acoustic energy to one propagation direction, whereas that of the conventional IDT structure spreads bidirectionally, as verified by simulated displacement fields (Fig. 6d). Experimentally, the SPUDT delay line without the AE film exhibited a lower insertion loss of 5.2 dB compared to 7.2 dB for the conventional IDT (Fig. 6e). Following the deposition of the AE film, the output current ratio showed that the SPUDT-based device consistently generated a higher current across the tested input powers (Fig. 6f), confirming improved energy utilization.

Under the conditions of 10 dBm RF input at a 10 cm wireless distance, the multi-IDT array and SPUDT structures achieved average current enhancements of approximately 113% and 43%, respectively, compared with the conventional IDT delay line, which exhibited an average current of 0.71 μA.

### Preliminary evaluation of AE stimulation in vivo

To evaluate the feasibility of wireless AE stimulation in vivo, we investigated the device in a rat full-thickness skin defect model under wireless RF excitation. The wireless configuration avoids the need for transcutaneous leads, enabling operation in freely moving subjects. Complete experimental procedures, in vivo datasets, histological analyses, and corresponding figures are provided in Supplementary Note IX. These preliminary results indicate a trend toward accelerated wound closure under low-intensity AE stimulation. However, the current output level is constrained by the relatively low RF-to-DC conversion efficiency. In practical wireless scenarios, achieving higher stimulation currents would require larger receiving antennas or improved system-level efficiency. These findings demonstrate the feasibility of wireless AE current delivery in a biological setting, while further improvements in conversion efficiency, device integration, and systematic validation will be required to assess broader biomedical applications. The demonstrated capabilities of stable current delivery, tunable output, and wireless operation support further development of AE current sources for biological and electronic systems.

## Discussion

This study presents an acoustic-driven current source exploiting the AE effect between SAWs and semiconductors, distinguishing it from the conventional voltage-controlled current sources. Under the wired operation, the device exhibits a linear relationship between output current and input RF power (*R*^2^ = 0.999) and provides continuously tunable outputs ranging from 1.22 nA to 25.46 μA. At an input RF power of 10 mW, the device delivers a stable output current with a standard deviation of 4.82 nA over 12 h of continuous operation. While the commercial integrated circuits offer highly stable current regulation, this acoustic-driven current source demonstrates the physical viability of achieving precisely tunable nA-to-µA currents through a fundamentally distinct, rectifier-free AE mechanism.

Under the wireless RF excitation, the device delivers tunable nanoampere-level currents while maintaining long-term stability. At a wireless input of 10 dBm and a 10 cm transmission distance, the conventional single-IDT design generates approximately 0.71 µA, whereas the optimized multi-IDT array and SPUDT structures increase the output by about 113% and 43%, respectively, by enabling cooperative operation and directional acoustic energy propagation.

The preliminary in vivo evaluation in a rat wound model demonstrates that the AE current source can be wirelessly excited to provide low-intensity electrical stimulation. Further investigations involving optimized device configurations, improved energy conversion efficiency, and systematic biological validation will be required to assess its broader biomedical potential. These results demonstrate the feasibility of wireless AE current delivery and support future exploration of this platform for low-current biological stimulation and other miniaturized electronic systems.

Although the proposed acoustic-driven current source demonstrates a rectifier-free mechanism and shows significant potential for specific bioelectronic applications, it is needed to acknowledge its current performance boundaries if compared to those using conventional semiconductor technologies. Firstly, the low RF-to-DC power conversion efficiency of the AE effect in the current single-device configuration is lower than that of conventional rectification circuits^32,33^. As shown in Supplementary Note X, which compares output DC power and input RF power, the observed efficiency is orders of magnitude lower than that typically reported for rectification circuits. Consequently, this technology is not intended to serve as a direct replacement for conventional rectifiers in scenarios which demand the maximum point-to-point power transfer efficiency. In contrast, in the ambient or broadcast RF environments, its simplified architecture allows multiple distributed AE devices to be excited simultaneously. As demonstrated in this study, a multi-IDT array can scale up the harvested current to meet practical application thresholds. Secondly, the low conversion efficiency of the developed device presents its constraints for practical implementation in clinical biomedical interfaces when realistic radiofrequency exposure regulations are considered. According to the IEEE Standard for Safety Levels (IEEE C95.1-1991), the maximum safe continuous power density on the human body at 430 MHz is limited to approximately 0.3 mW·cm^−2^. If the device were required to operate within these safe exposure limits to harvest RF power, the required receiving antenna area would be impractically large, even if the therapeutic current were limited to just 10 μA. This imposes a constraint on the trade-off between device miniaturization and transmission distance. Finally, the current device exhibits a moderate output impedance and a voltage compliance in the range of tens to hundreds of millivolts. While these characteristics may present challenges for directly driving standard high-impedance silicon logic gates without supplementary voltage boosting, they are applicable for a variety of low-voltage and low-impedance micro-loads. Specifically, the device is matched for driving biological interfaces^34,35^, where continuous nA-to-µA level currents are functionally effective. Consequently, the present work should primarily be regarded as a fundamental demonstration of an alternative AE mechanism for direct RF-to-DC conversion. Future advances in material properties, including enhancing the electromechanical coupling coefficient and semiconductor’s carrier mobility, together with improvements in device architecture and system-level design, will be required before this approach can be considered for broader wireless power transfer applications.

## Methods

### Animal study

All animal procedures were approved by the Animal Ethics Committee of Wuhan Myhalic Biotechnology Co., Ltd. (approval No. HLK-20250805-001) and were performed in accordance with the institutional guidelines for the care and use of laboratory animals.

Twenty-four male Sprague–Dawley (SD) rats (180–200 g, 6–7 weeks old) were obtained from Wuhan Myhalic Biotechnology Co., Ltd., and housed under specific pathogen-free (SPF) conditions at a controlled temperature of 22–26 °C, relative humidity of 50–60%, and a 12 h light/dark cycle. All animals had ad libitum access to standard rodent chow and drinking water.

After a 7 day acclimatization period, a full‑thickness skin defect (1 cm diameter) was created on the dorsum of each rat. The animals were randomly divided into three groups (*n* = 8 per group): a control group receiving no stimulation, a low‑intensity electrical stimulation (LI‑ES) group receiving 10 μA, and a high‑intensity electrical stimulation (HI‑ES) group receiving 20 μA. Stimulations were applied once daily for 20 min over 11 consecutive days via surface electrodes placed on both sides of the wound. Wound closure was monitored by photographing the wounds at regular intervals, and relative wound areas were quantified using ImageJ. On day 11, skin tissues were harvested for histological examination (Hematoxylin and eosin staining and Van Gieson staining). Complete details of surgical procedures, stimulation protocols, staining, and statistical analyses are provided in Supplementary Note IX.

### Device fabrication

The active region of the current source, which included the IDTs and the multilayer film, had a compact dimension of approximately 2.3 mm × 0.5 mm × 0.5 mm (length × width × height), reflecting the device’s miniaturized design. The IDTs were patterned using the conventional photolithography process. Each IDT was comprised of 30 finger pairs with a period of 8.92 µm. A Cr/Al (5 nm/270 nm) bilayer was subsequently deposited on the wafer using an e-beam evaporator, followed by a lift-off process to form the IDTs. The aperture of the IDTs was 223 μm, and the propagation length was 1784 μm. The SiO_*x*_/InSb/In_0.6_Al_0.4_Sb film with a length of 1200 μm was deposited on a 128°YX LiNbO_3_ substrate using an RF sputter (Technol, JCP500). There is a lattice mismatch between LiNbO_3_ and InSb, and oxygen diffusion from LiNbO_3_ into InSb leads to a degradation in the performance. Therefore, an In_*y*_Al_1−*y*_Sb buffer layer was inserted between InSb and the substrate, and this buffer layer was deposited by co-sputtering from InSb, Al, and Sb targets (diameter of 3 inches, with a purity of 99.99%). This buffer layer exhibited a high resistivity of approximately 2000 Ω·cm. This ensures that it does not short-circuit the piezoelectric field of the SAWs, thereby allowing the efficient coupling of acoustic energy to the charge carriers in the overlying InSb layer, which is essential for a strong AE effect. Moreover, a SiO_*x*_ layer was strategically incorporated into the structure to mitigate Sb’s out-diffusion from InSb during annealing, thereby improving the electrical properties of the heterostructure film. Subsequently, the device was annealed at 500 °C for 1 h. Annealing at 500 °C for 1 h showed a negligible impact on the piezoelectric coefficient *d*_33_ of LiNbO_3_. The *d*_33_ values were measured at five different locations on the substrate using a *d*_33_ meter (ZJ-3, Institute of Acoustics, Chinese Academy of Sciences) before and after annealing; the average change was less than 2.5% (Supplementary Note XI). Ag electrodes with a thickness of 200 nm for current measurements were deposited on both sides of the AE film using a thermal evaporator (Technol, ZHD400).

### Experimental design and characterization

The current source was fixed on a specially designed printed circuit board (PCB), and aluminum wires were applied for the connection between the current source and the measurement system. To ensure stability and minimize fluctuations in the output currents, a shielding enclosure, compatible with the dedicated adapter plate, was incorporated into the system. This shield protected the acoustic waves from the external electromagnetic field interferences and minimized the effects of light on the migration of charge carriers within the AE film. Sub-Miniature version A (SMA) connectors were used for the input and outputs of the RF signals, and the Bayonet Neill-Concelman (BNC) connectors were employed for the outputs of the AE currents. The transmission spectrum *S*_21_ of the device was obtained via a network analyzer (Agilent, E5071C). The RF signal source (Stanford Research Systems, SG394) was used to produce the RF signal to the busbar of the IDT to excite acoustic waves, and a power amplifier (Rflight, NTMSWPA-008102) was employed to enhance the energy of the RF signal. The AE current was measured using the source measurement unit (Keithley, 2450), configured with the output voltage set to be 0 V. The acoustic-driven current source measurement system is shown in Supplementary Note XII. To ensure measurement accuracy, an isolated grounding scheme was adopted, in which the ground of the source measurement unit was electrically isolated from that of the acoustic-driven current source, effectively eliminating the ground loops and minimizing errors caused by the ground potential differences (see Supplementary Note XIII for validation of this configuration). Furthermore, time-domain transient measurements utilizing a high-frequency transimpedance amplifier confirmed that the intrinsic dynamic response time (rise/fall time) of the AE current generation was less than 1 μs, demonstrating the device’s capability for rapid signal tracking (see Supplementary Note XIV). Moreover, under the continuous RF excitation, the device produced a DC output. When the RF signal was amplitude-modulated, a corresponding AC current was observed, demonstrating both the device’s potential as an AC source and confirming its response characteristics (Supplementary Note XV).

### Reporting summary

Further information on research design is available in the Nature Portfolio Reporting Summary linked to this article.

## Supplementary information

Supplementary Information

Reporting Summary

Transparent Peer Review file

## Source data

Source Data

## Data Availability

All data supporting the findings of this study are available within the paper and its Supplementary Information. Source data are provided in this paper.
