# Supplementary material for: Wireless acoustic-driven current source achieving nanoampere-level at millimeter-scale
Source: Nat Commun. 2026 Sep 28;17:11181. doi: 10.1038/s41467-026-78004-y (PMC13714251; doi:10.1038/s41467-026-78004-y)
Supplement: Supplementary file 1 [file 41467_2026_78004_MOESM1_ESM.pdf]

Supplementary Information

Wireless Acoustic-Driven Current Source Achieving Nanoampere-Level at Millimeter-Scale

Yang Yuan[1, 2, 3, #], Shengkuo Zhang[1, #], Jikai Zhang[4, #], Zhuo Liu[5], Yuxiang Wang[1], Wanli Yang[6], Xi Chen[1], Yanghui Liu[1], Fa Chen[1], Kuan Ning[1], Kunkun Yan[1], Guangzu Zhang[1], Qiuyun Fu[1], Yongqing Fu[7,8], Tao Sun[9] and Wei Luo[1, *]

[1] School of Integrated Circuits, Huazhong University of Science and Technology, Wuhan, PR China

[2] School of Microelectronics, Jiangxi Normal University, Nanchang, PR China

[3] Jiangxi Provincial Key Laboratory of Advanced Electronic Materials and Devices, Jiangxi Normal University, Nanchang, PR China

[4] Naval University of Engineering, Wuhan, PR China

[5] Tongji Medical College, Huazhong University of Science and Technology, Wuhan, PR China

[6] School of Aerospace Engineering, Huazhong University of Science and Technology, Wuhan, PR China

[7] School of Engineering, Physics and Mathematics, Northumbria University at Newcastle, Newcastle upon Tyne, NE1 8ST, UK

[8] School of Instrument Science and Technology, Xi'an Jiaotong University, Xi'an, PR China

[9] State Key Laboratory of High End Heavy Load Robots, Foshan, PR China

[#] These authors contributed equally: Yang Yuan, Shengkuo Zhang and Jikai Zhang.

*Corresponding author: Prof. Wei Luo, E-mail: luowei@hust.edu.cn

CONTENTS

I. Optimization of Film Thickness and Composition S2

II. Simulation Results of Film Thickness on Piezoelectric Substrate Characteristics S5

III. Frequency Response Characteristics of the SAW Device S8

IV. Output Voltage Range Characterization of the Acoustic-Driven Current Source S9

V. Simulation of Power-Dependent Steady-State Temperature Distribution S10

VI. The Reasons for Selecting 5 Hz for Noise Density Analysis S11

VII. DC Output Impedance of the Acoustic-Driven Current Source S13

VIII. Temperature Stability of the Acoustic-Driven Current Source S14

IX. Electrical Stimulation to Facilitate Acute Wound Healing S17

X. Comparison of RF-to-DC Conversion Efficiency S21

XI. The Effect of Annealing Process on $LiNbO_3$ Substrates S23

XII. The Acoustic-Driven Current Source Measurement System S25

XIII. Validation of Isolated Grounding Configuration S26

XIV. Characterization of Acoustoelectric Current Response Time S28

XV. AC Current Source Testing Results S30

References S33

I. Optimization of Film Thickness and Composition

1. Mechanism of Lattice Mismatch Mitigation by the Buffer Layer

As predicted by the Vegard's Law[1], the selected buffer layer, $In_{0.6}Al_{0.4}Sb$, has a lattice constant between those of AlSb (6.135 Å) and InSb (6.479 Å). However, there exists a significant lattice mismatch between $LiNbO_3$ and InSb, which would lead to a high density of crystal defects if the InSb is directly deposited onto $LiNbO_3$. The lattice parameter of $In_{0.6}Al_{0.4}Sb$ is quite compatible with that of InSb, facilitating the epitaxial growth of high-quality InSb films on top of the buffer layer and significantly reducing dislocations and structural defects.

Besides, this paper verified the structural improvement experimentally by conducting grazing incidence X-ray diffraction (GIXRD) on the prepared samples with and without using the buffer layer. The introduction of $In_{0.6}Al_{0.4}Sb$ leads to much stronger diffraction peak intensities and narrower full width at half maximum (FWHM) values for InSb, confirming the enhanced crystalline quality (Manuscript Fig. 2).

2. Mechanism of Oxygen Diffusion Suppression by Using the Buffer Layer

Oxygen diffusion from $LiNbO_3$ into the overlying InSb layer can seriously deteriorate its electrical performance[2].

From the structural aspect, although the earlier studies have used dielectric layers such as SiO_x for partial isolation[3], their loose structure, poor thermal stability, and lack of effective oxygen-trapping mechanisms limit the blocking effectiveness. In contrast, $In_{0.6}Al_{0.4}Sb$, being as a III–V compound semiconductor material, possesses high crystallinity and thermal stability, mainly attributed to its continuous barrier interface.

From the chemical aspect, the presence of Al atoms in the $In_{0.6}Al_{0.4}Sb$ enables the formation of strong Al-O bonds, providing its superior ability to suppress oxygen migration from $LiNbO_3$. This facilitates the chemical trapping of diffusing oxygen species, further enhancing the blocking efficiency of such a buffer layer.

3. Impact on Electrical Properties

This paper fabricated two sets of multilayer structures on $LiNbO_3$ substrates, i.e., SiO_x/InSb and SiO_x/InSb/$In_{0.6}Al_{0.4}Sb$, and performed the Hall effect measurements. The

measurement results showed that the sample with the buffer layer exhibited an increased mobility of 1667 $cm^2 \cdot V^{-1} \cdot s^{-1}$ (compared to 523 $cm^2 \cdot V^{-1} \cdot s^{-1}$ without the buffer), along with a lower carrier concentration of 6.94×10^{11} cm^{-2}. These results indicate the improved crystal quality and interface properties (Manuscript Fig. 2).

4. Thickness of the Buffer and Active Layers, and Measurement Methods

In this study, the $In_{0.6}Al_{0.4}Sb$ buffer layer was designed with a thickness of 150 nm, and the active InSb layer was 250 nm thick. Both the thicknesses were confirmed by using the cross-sectional scanning electron microscope (SEM). The selection of these values was mainly based on the following considerations[4]:

(1) For the buffer layer, a moderate thickness is essential to reduce the defect density and enhance the crystalline quality of the overgrown InSb film. However, an overly thick buffer could short-circuit the accompanying electric field of surface acoustic waves (SAWs), limiting its ability to penetrate and interact with carriers in the active layer. A thickness of 150 nm was selected to balance the good crystalline quality with acoustic-electric interaction efficiency.

(2) For the InSb active layer, insufficient thickness leads to poor crystallinity and reduced electron mobility, while excessive thickness enhances surface conductivity, thereby weakening the coupling between SAWs and the electron system. By controlling the sputtering time, a thickness of 250 nm for the InSb layer was selected to ensure both high carrier mobility and effective acoustic-electric coupling.

5. Atomic Composition of the Buffer Layer and Its Impact on Electrical Properties

The $In_{0.6}Al_{0.4}Sb$ buffer layer was fabricated using a co-sputtering process involving three targets of InSb, Al, and Sb targets. By adjusting the sputtering power of the InSb target, this paper systematically investigated the influences of In/Al atomic ratios on the electrical properties of the multilayer structure (Supplementary Fig. 1). The elemental ratios were determined via energy-dispersive X-ray spectroscopy (EDS), and the results are shown in Supplementary Table 1.

(1) Carrier mobility initially increases and then decreases with increasing In/Al ratio,

reaching a peak value of 631 $cm^2 \cdot V^{-1} \cdot s^{-1}$ when In/Al ≈ 0.56/0.44.

(2) Sheet carrier concentration exhibits an inverse trend with the above, reaching a minimum value of 1.64×10^{12} cm^{-2} at the same composition.

These trends are attributed to the interplays among lattice matching, crystalline quality, and surface roughness. Importantly, the samples in this sputtering-power series were prepared for composition optimization and trend analysis and are distinct from the final heterostructure used for device fabrication. The series identified a favorable In/Al compositional ratio, which provided guidance for subsequent optimization of other fabrication parameters, including growth temperature and annealing temperature/time. Because the growth and thermal-processing conditions can affect the elemental distribution and actual In/Al ratio of the buffer layer, the final buffer composition ($In_{0.6}Al_{0.4}Sb$) was determined based on the combined optimization of the sputtering conditions, growth temperature, and annealing conditions.

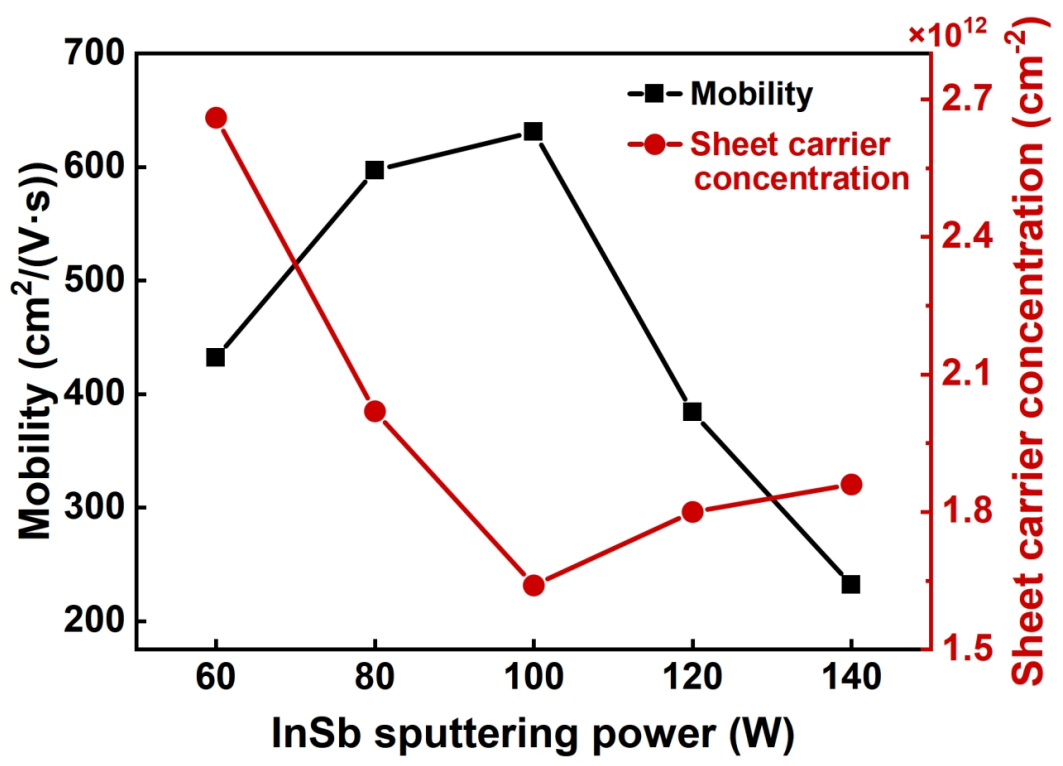

Supplementary Fig. 1. Carrier mobility and sheet carrier concentration of SiO_x/InSb/$In_yAl_{1-y}Sb$ films as a function of InSb sputtering power (60–140 W). Source data are provided as a Source Data file.

Supplementary Table 1. Atomic percentages of each element in $In_yAl_{1-y}Sb$ films fabricated at different InSb sputtering powers

InSb sputtering power (W)	In (at%)	Al (at%)	Sb (at%)	In/Al	$In_yAl_{1-y}Sb$
60	18.29	32.00	49.71	0.57	$In_{0.36}Al_{0.64}Sb$
80	24.56	25.45	49.99	0.97	$In_{0.49}Al_{0.51}Sb$
100	27.98	22.26	49.76	1.26	$In_{0.56}Al_{0.44}Sb$
120	30.60	19.60	49.81	1.56	$In_{0.61}Al_{0.39}Sb$
140	32.64	17.14	50.22	1.90	$In_{0.66}Al_{0.34}Sb$

II. Simulation Results of Film Thickness on Piezoelectric Substrate Characteristics

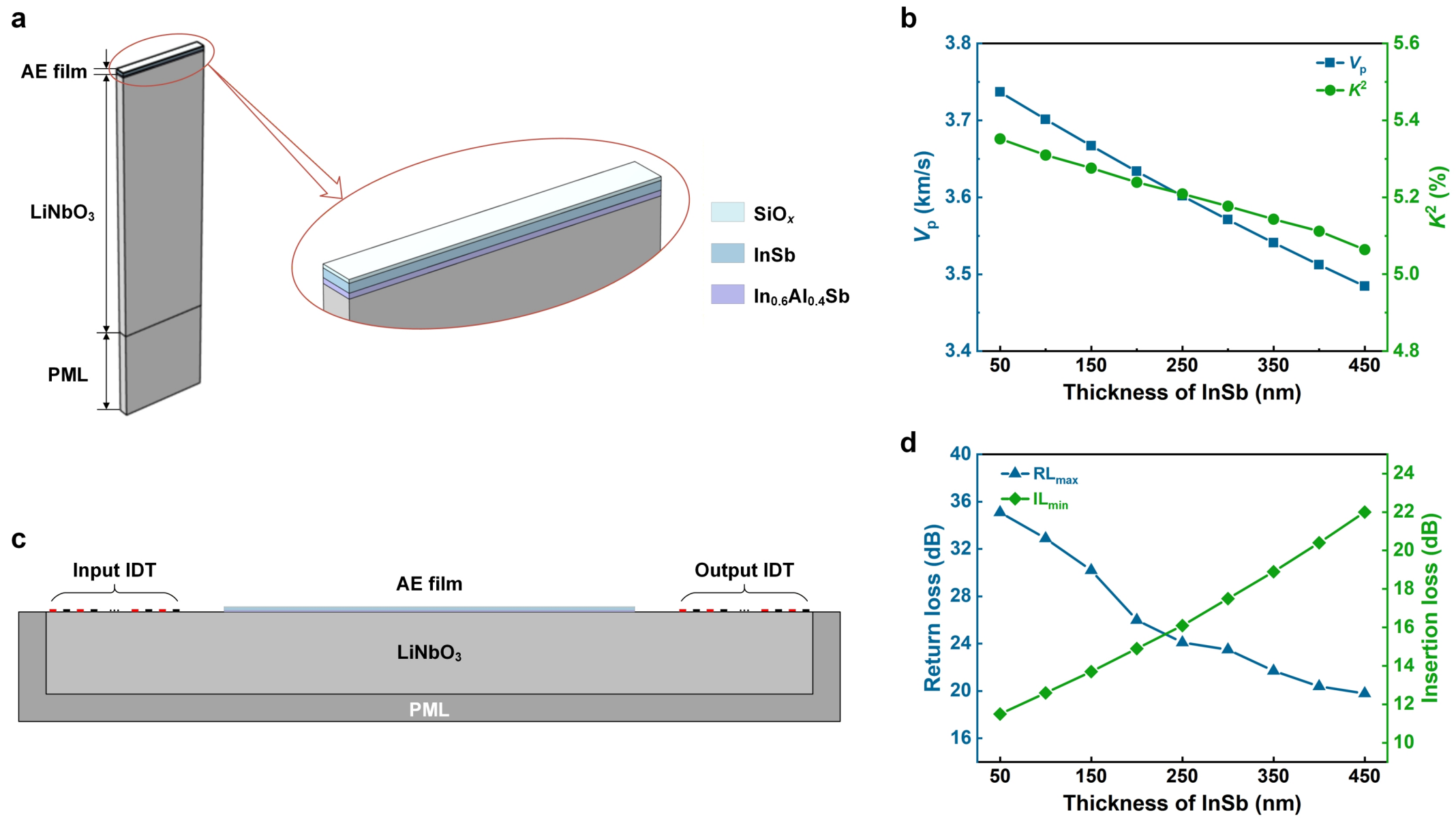

Supplementary Fig. 2. a A periodic unit finite element model of the AE film region. **b** Rayleigh wave velocity V_p and electromechanical coupling coefficient K^2 as functions of InSb thickness. **c** Schematic diagram of the cross-section of the acoustic-driven current source along the acoustic wave propagation direction. **d** Maximum return loss RL_{max} and minimum insertion loss IL_{min} as functions of InSb thickness. Source data are provided as a Source Data file.

The total thickness of the acoustoelectric (AE) film used in the experiments was 500 nm, with the thickness of each layer being SiO_x (100 nm)/InSb (250 nm)/$In_{0.6}Al_{0.4}Sb$ (150 nm). This paper is focused on the active layer InSb, which plays a dominant role in the AE film, and the effects of its layer thickness was characterized on the Rayleigh wave velocity V_p, electromechanical coupling coefficient K^2, and reflection properties. A periodic unit finite element model of the AE film region was established (Supplementary Fig. 2a) to analyze the V_p and K^2 of InSb with different thicknesses (ranging from 50 nm to 450 nm), where the thicknesses of the SiO_x and $In_{0.6}Al_{0.4}Sb$ films were fixed at 100 nm and 150 nm, respectively. In addition, by simulating the cross-sectional model of the acoustic-driven current source along the acoustic wave propagation direction (Supplementary Fig. 2c), the S-parameters of the device were obtained, enabling the calculation of the variations between the maximum

return loss RL_{max}, minimum insertion loss IL_{min}, and the thickness of the InSb film.

Supplementary Fig. 2b and d show the variations between V_p, K^2, RL_{max}, IL_{min} and the thickness of InSb, where the calculation formulas for V_p and K^2 are as follows:

$$V_p = \frac{V_f + V_m}{2} \tag{S1}$$

$$K^2 = \frac{V_f^2 - V_m^2}{V_f^2} \tag{S2}$$

As the thickness of InSb increases from 50 nm to 450 nm, both V_p and K^2 show a decreasing trend (Supplementary Fig. 2b), with V_p varying from 3737 $m \cdot s^{-1}$ to 3484 $m \cdot s^{-1}$ and K^2 ranging from 5.35% to 5.06%. RL_{max} and IL_{min} values exhibit opposite variations (Supplementary Fig. 2d), i.e., the former drops with increasing InSb film thickness (from 35.1 dB to 19.8 dB), whereas the latter grows continuously (from 11.5 dB to 22 dB).

From the perspective of physical mechanism, the Rayleigh wave velocity fundamentally reflects the combined effect of the effective elastic modulus E_{eff} and effective density ρ_{eff} of the propagation medium, and V_p is proportional to $\sqrt{E_{eff}/\rho_{eff}}$. As the thickness of InSb increases, its weight in confining the energy of Rayleigh waves gradually increases. Since the InSb possesses a lower elastic modulus than the other layers (i.e., $LiNbO_3$ substrate, $In_{0.6}Al_{0.4}Sb$ and SiO_x) and a higher density, V_p decreases continuously. Simultaneously, a thicker InSb allows more acoustic energy to transmit from the substrate into the InSb layer, and part of the acoustic energy is absorbed or converted into electrical energy in InSb. This reduces the effective acoustic energy density participating in piezoelectric coupling within the substrate, ultimately leading to a continuous decrease in K^2.

The decrease in RL_{max} stems from the dual negative effects of InSb thickness on electrical impedance and acoustic reflection. On one hand, the equivalent input resistance of the InSb layer decreases with increasing thickness, increasing the

impedance mismatch. On the other hand, as the thickness of InSb increases, constructive superposition of acoustic reflection echoes at multilayer interfaces becomes possible, thus enhancing the total reflected acoustic energy. The increase in IL_{min} results from the dual effects of increased energy dissipation and reflection power encroaching on transmission power. The thicker layer of InSb increases the number of effective carriers, converting more acoustic energy into the kinetic energy of carriers. Meanwhile, the acoustic energy loss caused by the accumulation of lattice defects intensifies. In addition, the increase in reflected power reduces the effective transmitted acoustic energy, further decreasing the power at the output end of the delay line.

In the AE film described in this paper, the thickness of InSb was set to 250 nm, at which point the V_p and K^2 of the AE film region are 3602 $m \cdot s^{-1}$ and 5.21%, respectively. Although the thinner InSb exhibits larger V_p and K^2, its insufficient ability to confine acoustic energy limits the intensity of AE conversion. In contrast, excessively thick InSb increases acoustic wave reflection and energy dissipation. Based on these comprehensive considerations, it was finally determined that 250 nm is a relatively appropriate value.

III. Frequency Response Characteristics of the SAW Device

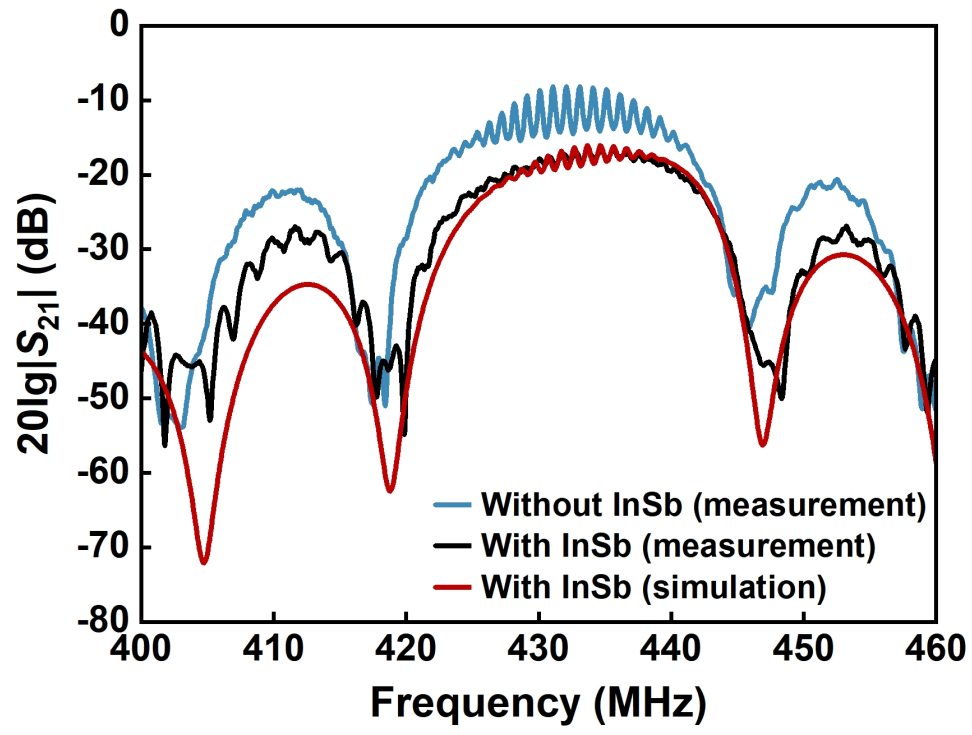

Supplementary Fig. 3. Frequency response characteristics of the SAW device before and after the deposition of $SiO_x/InSb/In_{0.6}Al_{0.4}Sb$ films. Source data are provided as a Source Data file.

Supplementary Table 2. Simulation and measurement results of the center frequencies and insertion loss without and with the $SiO_x/InSb/In_{0.6}Al_{0.4}Sb$ films.

Structures	Methods	Center frequency (MHz)	Insertion loss (dB)
Without film	Measurement	433.09	−8.13
With film	Simulation	434.66	−16.05
With film	Measurement	434.75	−16.74

Frequency response characteristics of the SAW device before and after the deposition of $SiO_x/InSb/In_{0.6}Al_{0.4}Sb$ films are shown in Supplementary Fig. 3. The AE film causes attenuation of the acoustic waves, leading to an increase in insertion loss from 8.13 dB to 16.74 dB. The COM model that includes the AE effect was employed in the simulation of the device's frequency responses[5]. A comprehensive description of the COM model, as well as the modelling of losses in the AE film, can be found in our previous work[6]. The obtained simulation and measurement results for the frequency response characteristics are summarized in Supplementary Table 2. The COM model analyzes the interactions between the acoustic waves and semiconductor carriers, which result in the attenuation and the change in SAW velocity. This model accurately predicted the scattering parameters of the device.

IV. Output Voltage Range Characterization of the Acoustic-Driven Current Source

The output voltage of the acoustic-driven current source was measured using a Keithley 2450 source measurement unit. The measurement was conducted under the same conditions as those used for evaluating the AE current, including the identical RF frequency, input power, and device configuration. As shown in Supplementary Fig. 4, the recorded output voltage ranges from 0 to approximately 335 mV.

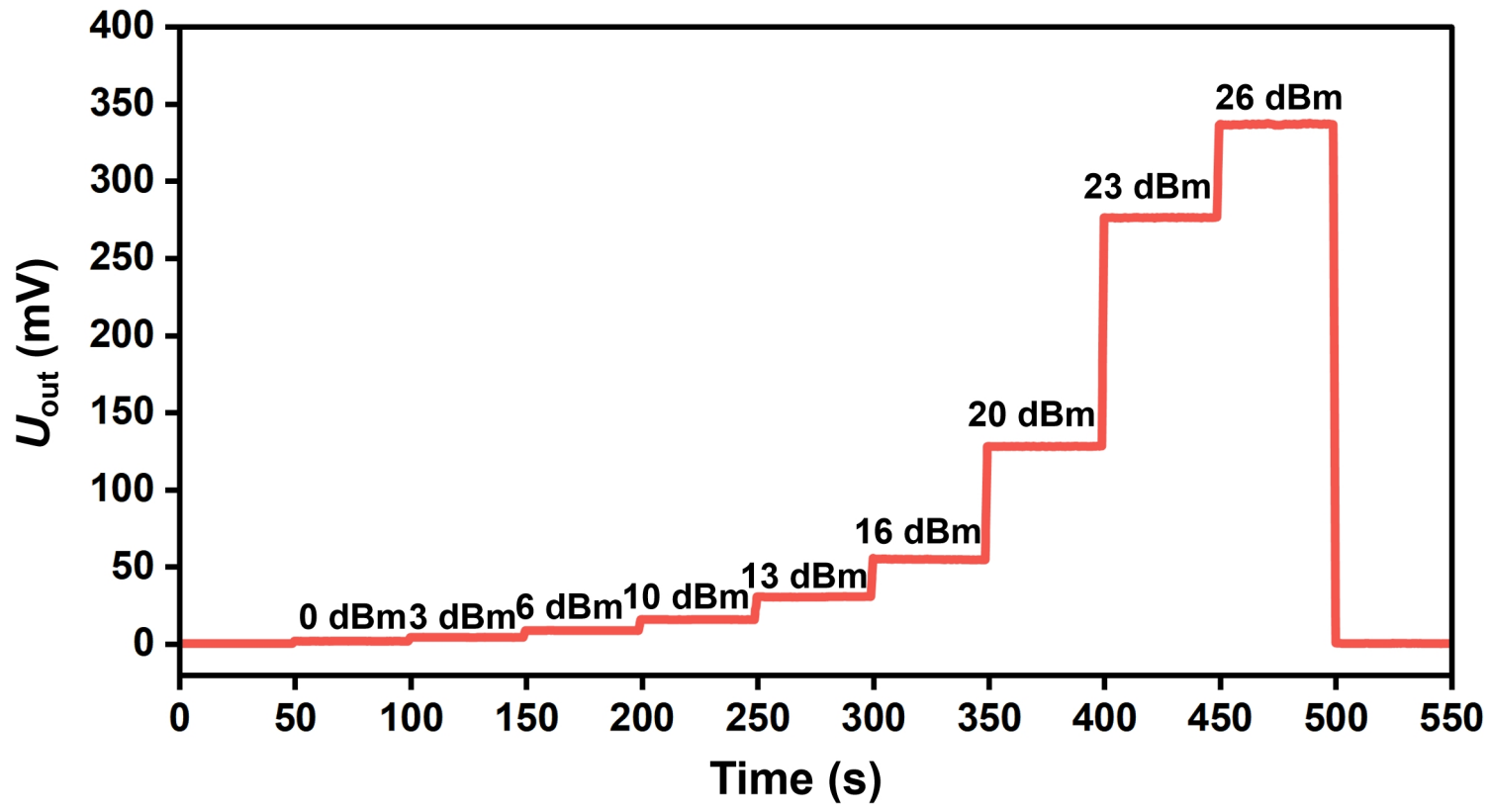

Supplementary Fig. 4. Output voltage range of the acoustic-driven current source. Source data are provided as a Source Data file.

V. Simulation of Power-Dependent Steady-State Temperature Distribution

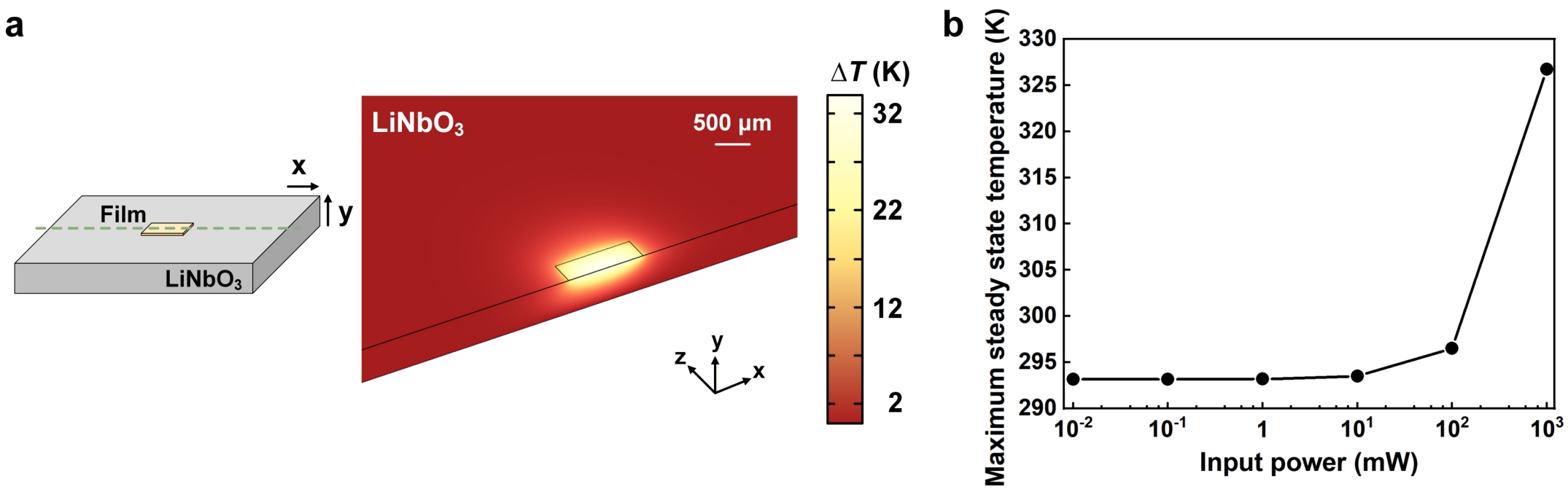

Supplementary Fig. 5. Power-dependent steady-state thermal response of the acoustic-driven current source. **a** The simulated steady-state distribution of the temperature difference (ΔT) is shown at an RF power of 1 W, with the green dashed line indicating the simulated cross-section. **b** The maximum steady-state temperature as a function of input RF power from 10 μW to 1 W. Source data are provided as a Source Data file.

The heat dissipation efficiency of the semiconductor multilayer region was dependent on convection into the air and thermal conduction into the substrate. To evaluate the thermal response of the multilayer film region, the semiconductor multilayer region consisting of InSb and $In_{0.6}Al_{0.4}Sb$ was modeled as a single equivalent thermal layer. The simulated steady-state temperature distributions were obtained using an equivalent thermal layer with dimensions of 1200 μm × 500 μm × 400 nm. The effective thickness of 400 nm was used to approximate the combined thickness of the InSb and $In_{0.6}Al_{0.4}Sb$ layers, rather than the physical thickness of the InSb active layer. This equivalent thermal layer was placed on a $LiNbO_3$ substrate with dimensions of 10 mm × 8 mm × 0.5 mm. The thermal conductivity and constant-pressure heat capacity of InSb (3.28 $W \cdot m^{-1} \cdot K^{-1}$ and 601 $J \cdot kg^{-1} \cdot K^{-1}$, respectively) were used as approximate thermal parameters for this equivalent thermal layer. The simulated steady-state temperature distribution with the input RF power of 1 W is shown in Supplementary Fig. 5a, while those corresponding results as a function of RF power are shown in Supplementary Fig. 5b. A significant increase in the maximum steady-state temperature was evident as the power was increased from 0.1 W to 1 W, inducing changes in the output currents.

VI. The Reasons for Selecting 5 Hz for Noise Density Analysis

1. Reason for selecting 5 Hz

According to the Nyquist–Shannon sampling theorem, the upper limit of frequency analysis is half of the sampling frequency. In the measurements, the sampling frequency of the source measurement unit was set to be 12 Hz, resulting in a current frequency analysis upper limit of 6 Hz. Within this range, 5 Hz was selected as the representative frequency for noise density evaluation because it provides a stable indicator of current fluctuation while minimizing the influence of instrument-induced high-frequency noise. Increasing the sampling frequency would introduce additional parasitic noise from the instrument and cables, thereby reducing measurement accuracy in the low-frequency range.

2. Full noise spectrum under different RF powers

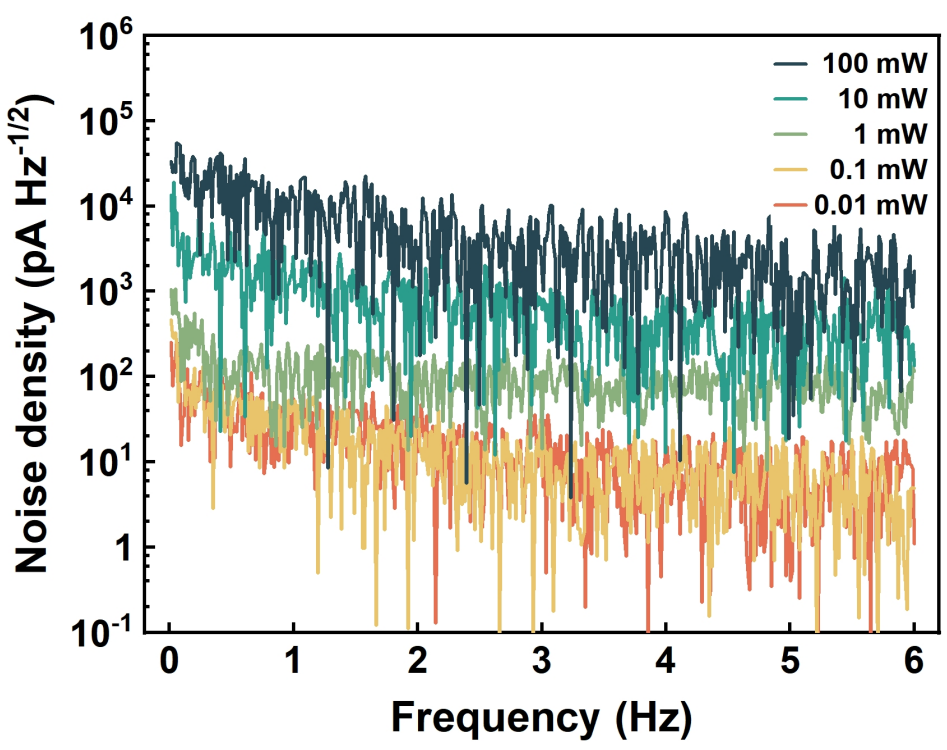

Supplementary Fig. 6. Current noise density spectra of the acoustic-driven current source under different RF input powers. Source data are provided as a Source Data file.

To further investigate the noise characteristics of the acoustic-driven current source, this paper analyzed the current noise spectra under different RF input powers (0.01 mW, 0.1 mW, 1 mW, 10 mW, and 100 mW), as shown in Supplementary Fig. 6. The noise density consistently exhibited a decreasing trend with increasing frequency, and the 5 Hz point was used as a representative reference for quantitative comparison across all input power levels.

3. On the presence of 1/f noise

It is acknowledged that 1/f noise often dominates in the low-frequency regimes. However, due to the limited measurement bandwidth (0.02–6 Hz) and the focus on

overall current stability, the present study did not quantitatively separate 1/f noise from other possible components such as thermal or shot noise. While a general downward trend in noise density with increasing frequency was observed, this alone was insufficient to conclusively confirm 1/f behavior.

In summary, 5 Hz was selected as the representative low-frequency reference for consistent comparisons, and the full noise spectra at different RF input powers were analyzed to ensure the reliability of the results. The potential presence of 1/f noise was acknowledged but was not quantitatively verified within the current measurement range.

VII. DC Output Impedance of the Acoustic-Driven Current Source

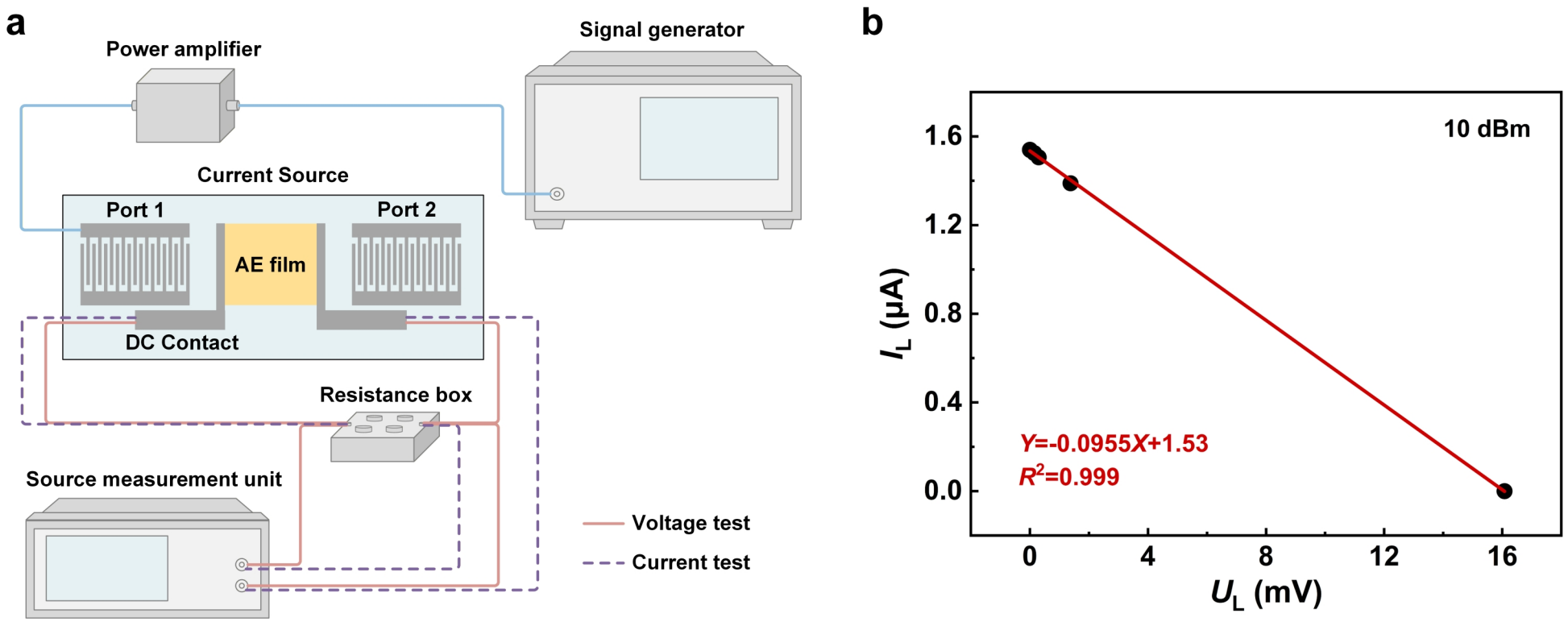

Supplementary Fig. 7. Output impedance of the acoustic-driven current source. **a** Schematic diagram of the test system. **b** IV measurement curve. Source data are provided as a Source Data file.

The DC output impedance R_{out} of the acoustic-driven current source was determined using the load-line method, i.e., by measuring the relationship between the terminal voltage U_L and the output current I_L across different load resistances R_L. Measurements were performed using a Keithley 2450 source measurement unit (Supplementary Fig. 7a).

The input power was fixed at 10 dBm, and R_L was sequentially set to 0 Ω, 10 Ω, 100 Ω, 200 Ω, and 1000 Ω. When $R_L = 0$ Ω, the measured short-circuit current I_{sc} was 1.54 μA. Whereas when R_L approached infinity, the measured open-circuit voltage U_{oc} was 16.1 mV. Fitting the linear U_L–I_L relationship (Supplementary Fig. 7b) yielded an output impedance of approximately 10.5 kΩ.

This load-line method provides a direct evaluation of the internal resistance of the current source under practical operating conditions. Future improvements include optimizing the thin-film growth process, introducing interface passivation layers to reduce defect density at the heterojunction, and lowering the carrier concentration in the semiconductor layer to increase the internal resistance.

VIII. Temperature Stability of the Acoustic-Driven Current Source

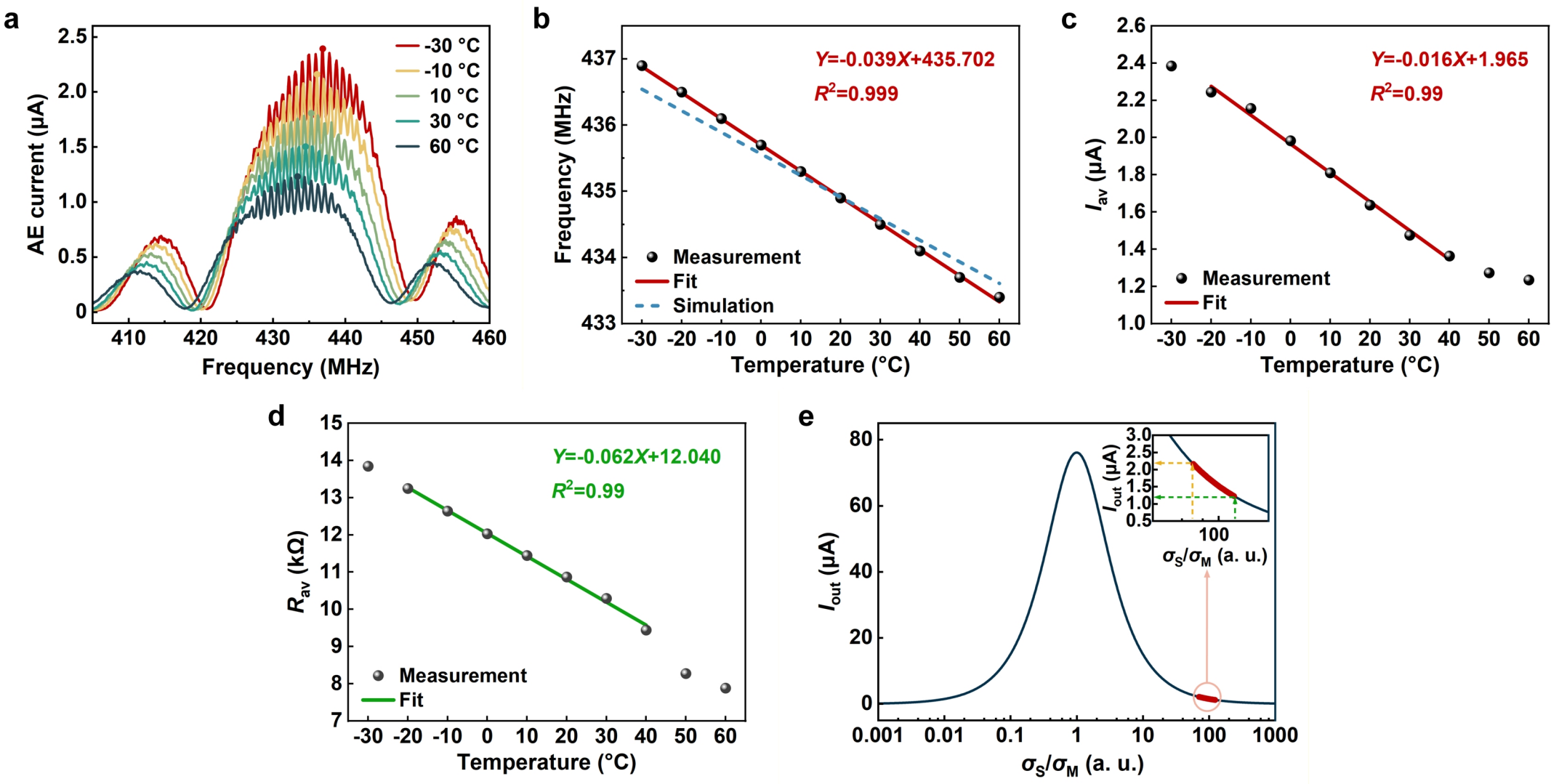

Supplementary Fig. 8. a The AE current as a function of frequency (the current at the center frequency labeled) under a sweeping 10 mW RF signal with temperatures ranging from −30 to 60°C. With a temperature interval set at 10°C, each temperature underwent four cycles, **b** The fitted relationship between frequency shift and temperature, where the blue dashed line indicates the theoretical frequency shift; **c** The fitted relationship between the average current at the center frequency and temperature. **d** The average resistance at different temperatures. **e** The theoretical current as a function of normalized sheet conductivity σ_S/σ_M. The red line indicates the experimentally observed current variation due to changes in film sheet conductivity, while the yellow and green curves indicate the upper and lower limits of current variation, respectively. Source data are provided as a Source Data file.

To investigate the temperature stability of the acoustic-driven current source, acoustoelectric (AE) current curves were recorded under a sweeping 10 mW RF signal with temperatures ranging from −30 to 60 °C, and the obtained results are shown in Supplementary Fig. 8a. The variable temperature conditions were induced within a testing chamber. As the temperature was increased, the AE current curves were shifted towards the lower frequency side, showing a significant decrease in AE current values, which were repeatedly tested for four cycles at each temperature with a 10°C interval. The obtained frequency shifts and average currents at the center frequency are shown in Supplementary Fig. 8b and c, respectively. Supplementary Fig. 8b shows a linear

relationship between frequency shift and temperature with a fitted slope of −39 kHz·°C^{-1} and a high correlation coefficient R^2 of 0.99. Notably, for all the repeated experiments at the same temperature, the center frequency of the device was remained a constant. The primary cause of the frequency shift was attributed to the poor temperature stability of the 128°YX $LiNbO_3$ substrate. The frequency f at different temperatures T can be expressed as follows,

$$\mathrm{TCF} = \frac{f - f_{T_{\mathrm{ref}}}}{f_{T_{\mathrm{ref}}} \times (T - T_{\mathrm{ref}})} \tag{S3}$$

where the temperature coefficient of frequency (TCF) for the 128° YX $LiNbO_3$ is -75×10^{-6} °C^{-1}, the reference temperature T_{ref} is 25 °C, and it corresponds to a frequency $f_{T_{\mathrm{ref}}}$ of 434.75 MHz. The predicted frequency shift due to temperature, based on Eq. (S3), indicates a decrease in the center frequency of the IDTs as the temperature is increased. The observed frequency shift in the experiment was aligned well with the theoretical prediction results, showing a consistently linear relationship. The measured actual temperature coefficient is -90×10^{-6} °C^{-1}, slightly exceeding the theoretical value, mainly attributed to the influences of film and circuitry. In comparison to most substrates used in the fabrication of SAW devices, AlN exhibits a lower temperature sensitivity with a frequency coefficient of -19×10^{-6} °C^{-1}. Depositing the AlN film on a 128°YX $LiNbO_3$ substrate effectively reduced the temperature coefficient while ensuring the propagation of SAWs[7]. The presence of an $In_{0.6}Al_{0.4}Sb$ buffer layer mitigates the influences of the AlN film on the electrical properties of the InSb film. These results indicate that employing a $LiNbO_3$ substrate with an AlN epitaxial film is a promise method to address the frequency shift observed in the acoustic-driven current source under temperature variations.

Supplementary Fig. 8c shows a notable linear relationship between the average current at the center frequency and temperature, obtained within the temperature range of −20 to 40 °C, with a fitted slope of −16 nA·°C^{-1} and a high correlation coefficient R^2 of 0.99. Notably, at lower or higher temperatures, there was an increased current fluctuation, leading to a decreased linearity. To elucidate the factors contributing to

current fluctuations, this paper conducted resistance measurements on the heterostructure films at different temperatures. The average resistance was decreased from 13.8 kΩ to 7.9 kΩ as the temperature was increased from −30°C to 60°C, with negligible fluctuations at the same temperature. The fitting relationship between average resistance and temperature is shown in Supplementary Fig. 8d. Due to the reciprocal relationship between resistance and conductivity, the film sheet conductivity σ_S exhibited a variation range of 1.4×10^{-4} to 2.5×10^{-4} S. According to Eq. (1), a functional correlation between the current at the center frequency and the normalized sheet conductivity σ_S/σ_M can be established at an input RF power of 10 mW, as shown in Supplementary Fig. 8e. This elucidates that the primary factor causing the variation in AE current is the change in film sheet conductivity σ_S. The current is maximized when $\sigma_S = \sigma_M$, resulting in the highest energy conversion efficiency under an identical input power. We further measured the conductivity, carrier concentration, and mobility of InSb single crystals in the temperature range of 1.3 to 700 K, which showed a strong temperature dependence of the electrical properties of InSb[8]. At the temperatures above 100 K, the lattice scattering effects were contributed to the increased sheet conductivity of InSb, accompanied by an increase in carrier concentration and a reduction in mobility.

Temperature drift has detrimental effects on the precision and stability of current sources, limiting their practical utility. Although acoustic-driven current sources exhibit a significant temperature drift for both frequency and current, a linear relationship is observed for both these parameters within the temperature range of −20 to 40°C, indicating its highly predictable and repeatable behaviors with temperature fluctuations. Therefore, temperature compensation techniques such as feedback control circuits can be utilized to detect environmental temperature and promptly adjust input RF signal frequency and power, effectively eliminating temperature effects and ensuring output current stability.

IX. Electrical Stimulation to Facilitate Acute Wound Healing

Electrical stimulation (ES) is a non-pharmacological therapeutic strategy that has received increasing attention in wound healing research[9]. To preliminarily explore whether the developed acoustic-driven current source could serve as a wireless ES platform for biological interfaces, we conducted an in-vivo evaluation using a rat model of acute full-thickness skin defects. As shown in Supplementary Fig. 9, the experiment consisted of three phases: a 7-day acclimatization period, an 11-day ES intervention period, and a subsequent histological examination period. Animals were randomly divided into three groups (*n* = 8 per group): a control group (no stimulation), a low-intensity ES group (LI-ES, 10 μA), and a high-intensity ES group (HI-ES, 20 μA). Stimulations were applied once daily for 20 minutes per session over 11 consecutive days (from Day 0 to Day 10).

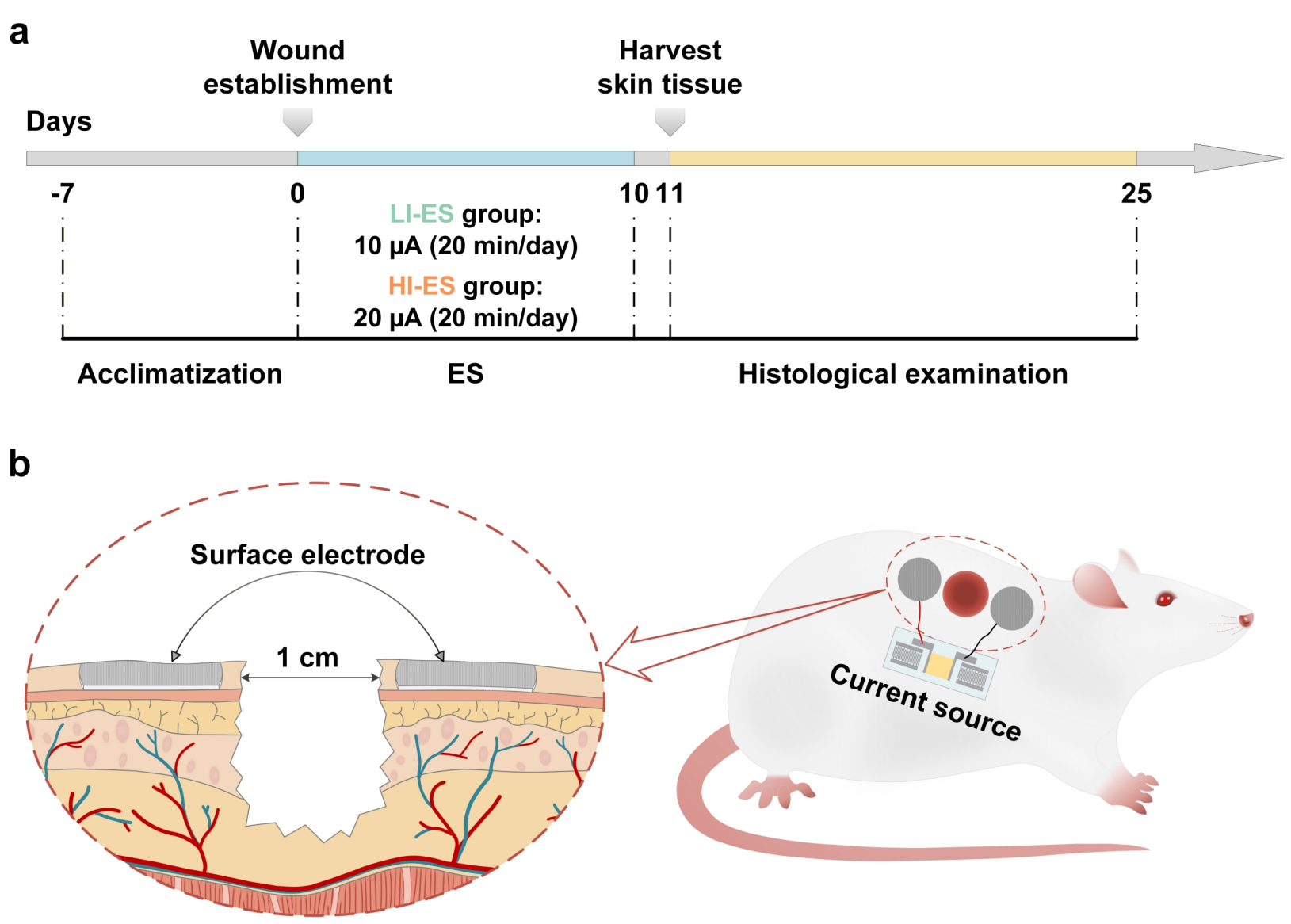

Supplementary Fig. 9. Acoustic-driven current source-based ES facilitates acute wound healing. **a** Experimental timeline. **b** Schematic of wound model and ES setup.

1. Experimental Methods

All animal experimental protocols were approved by the Animal Ethics Committee of Wuhan Myhalic Biotechnology Co., Ltd. (approval No. HLK-20250805-001); for basic animal information and ethics, see the Animal study subsection in the Methods of the main manuscript.

After anesthesia, the hair on both sides of the spinal column on the rat back was shaved. A 1 cm-diameter full-thickness skin wound was created on the right side of the spinal column, extending to the fascial layer. As shown in Supplementary Fig. 10, the LI-ES group was subjected to a current intensity of 10 μA using a wireless RF signal transmission system. A rubber antenna was mounted on the transmitter side, while a flexible printed circuit (FPC) antenna was used on the receiver side. The acoustic-driven current source was placed at the designated area of the FPC antenna and connected to the antenna port via aluminum wires. The HI-ES group was administered a current intensity of 20 μA via a wired RF signal transmission system. The control group received no electrical stimulation and was allowed to undergo spontaneous healing.

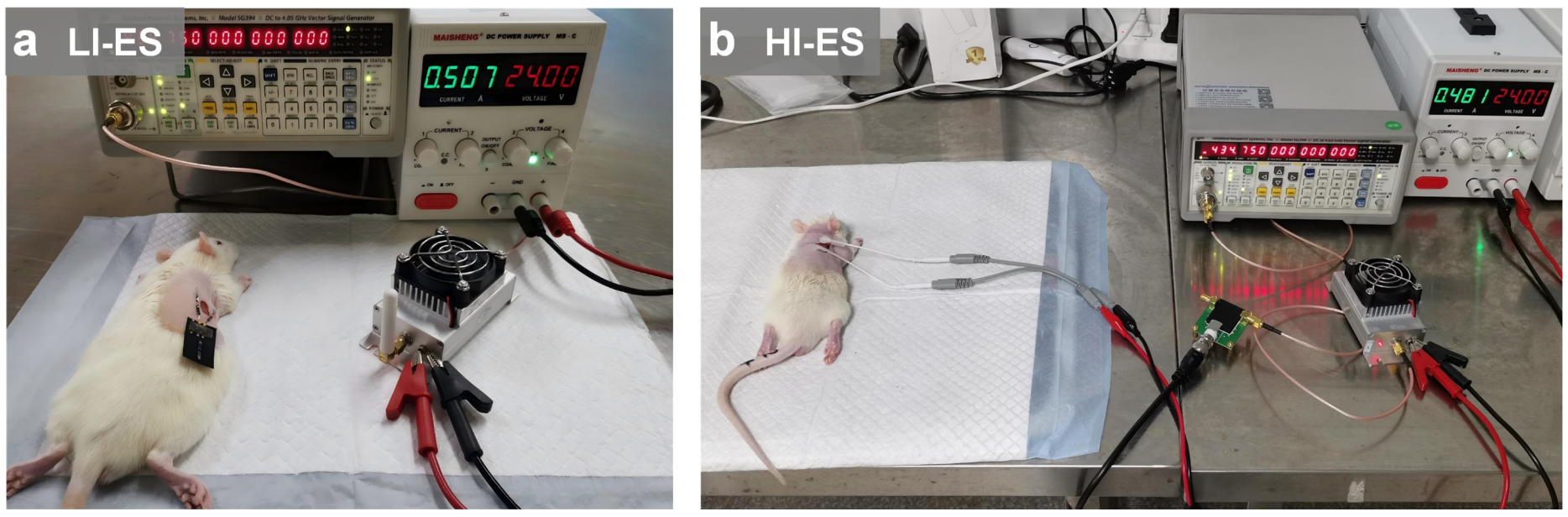

Supplementary Fig. 10. Photographs of the experimental setup. **a** LI-ES group. **b** HI-ES group.

Wound photographs of rats in these three groups were taken at the same time point on Days 0, 2, 4, 6, 9, and 11 under consistent distance and lighting conditions. The wound area at each time point was measured using ImageJ software, and the relative wound area was calculated as (wound area on Day *n* / wound area on Day 0). On Day 11, the rats were euthanized by overdose anesthesia, and intact skin tissue including the wound and 0.5 cm of the surrounding area was harvested. The tissue samples were fixed in formalin, embedded in paraffin, and sectioned. Skin sections from three randomly selected rats per group were subjected to hematoxylin and eosin (H&E) staining and Van Gieson (VG) staining. The epidermal and dermal repair status in the central area of the wound was observed under a light microscope. The provided histological images serve solely to demonstrate representative morphological features. Statistical analyses were performed using GraphPad Prism software. The Kruskal-Wallis H test was used

to compare wound areas among groups, followed by Dunn's multiple comparisons test for pairwise comparisons. A p value < 0.05 was considered statistically significant.

2. Experimental Results

(1) Changes in Wound Areas Over Time

The results of the relative wound area analysis for the three rat groups are presented in Supplementary Fig. 11. During the experimental period, two rats in the HI-ES group died. One representative rat was selected from each group, and the changes in wound appearance over time were recorded (as shown in Supplementary Fig. 12). From Day 0 to Day 4, no significant difference in wound appearance was observed among the three groups. On Day 6, the wound area of the LI-ES group was significantly smaller than that of the control group. Starting from Day 9, significant differences in wound area were observed between the HI-ES group and the control group. At Day 11, the LI-ES and HI-ES groups showed nearly complete wound closure, with significantly lower wound areas than the control group ($p < 0.05$). Although no statistically significant difference in wound area was detected between the LI-ES and HI-ES groups, the SD of the LI-ES group was decreased continuously from 0.154 (Day 2) to 0.011 (Day 11). This suggests that LI-ES treatment may reduce the variability of wound healing among individual rats within the group, contributing to more consistent therapeutic outcomes.

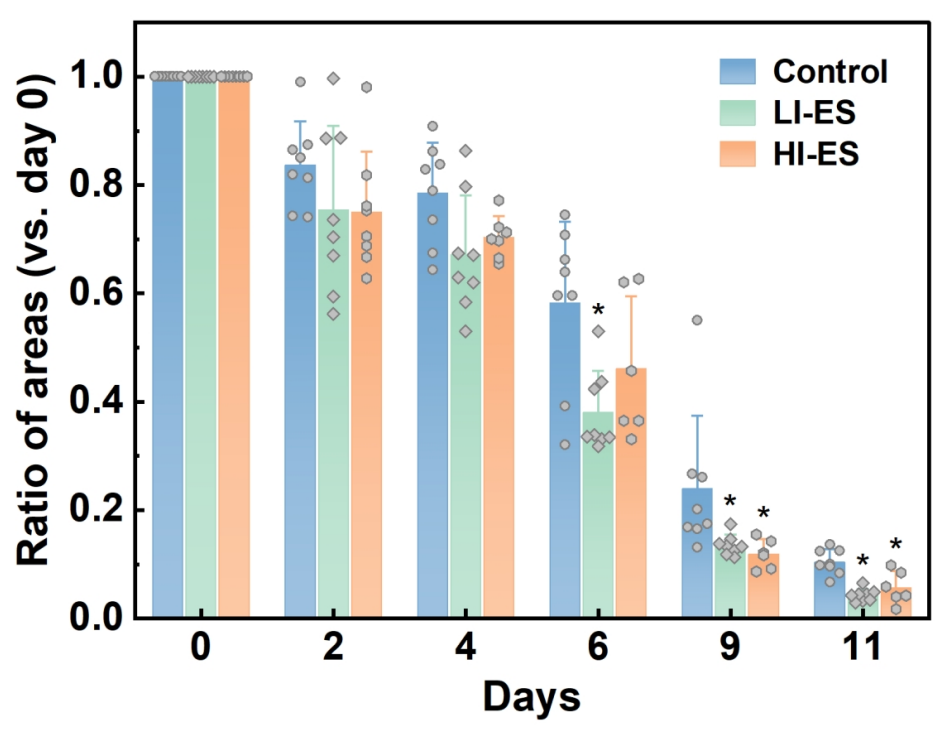

Supplementary Fig. 11. Relative wound areas over time for the three groups. Data are expressed as mean ± SD. *$p < 0.05$ vs. control group. Source data are provided as a Source Data file.

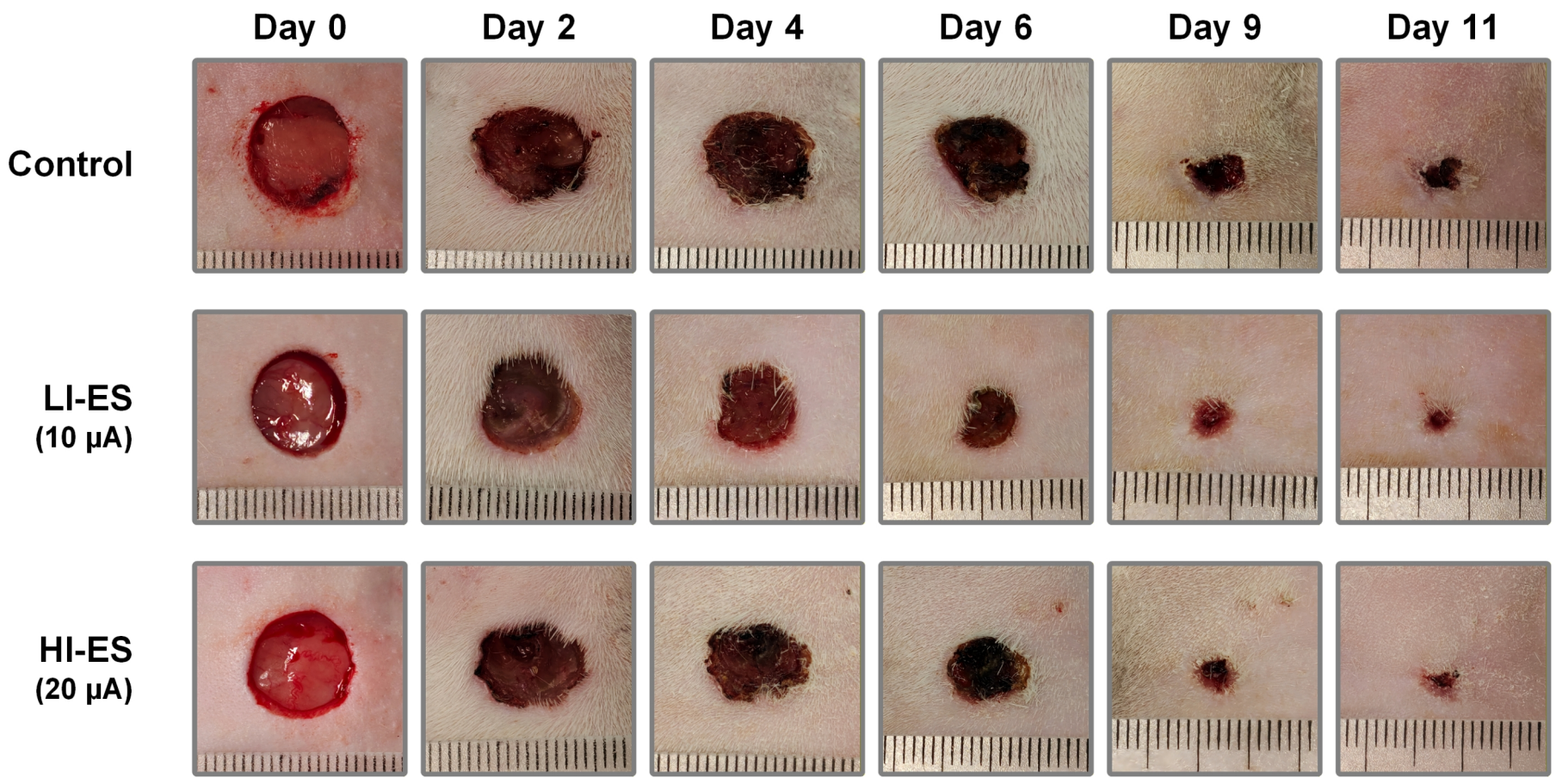

Supplementary Fig. 12. Changes in wound appearance of rats in the three groups over time.

(2) Histological Analysis of Skin Sections (Day 11)

Supplementary Fig. 13 presents representative H&E and VG staining images of skin sections from the three groups on Day 11. The H&E staining results indicated reduced inflammatory cell infiltration in the LI-ES and HI-ES groups compared with the control group, accompanied by enhanced epidermal regeneration. The VG staining results showed that the control group exhibited the least collagen deposition, with loosely arranged and disorganized collagen fibers. In contrast, the LI-ES group showed increased collagen deposition and denser collagen fiber organization compared with the HI-ES group.

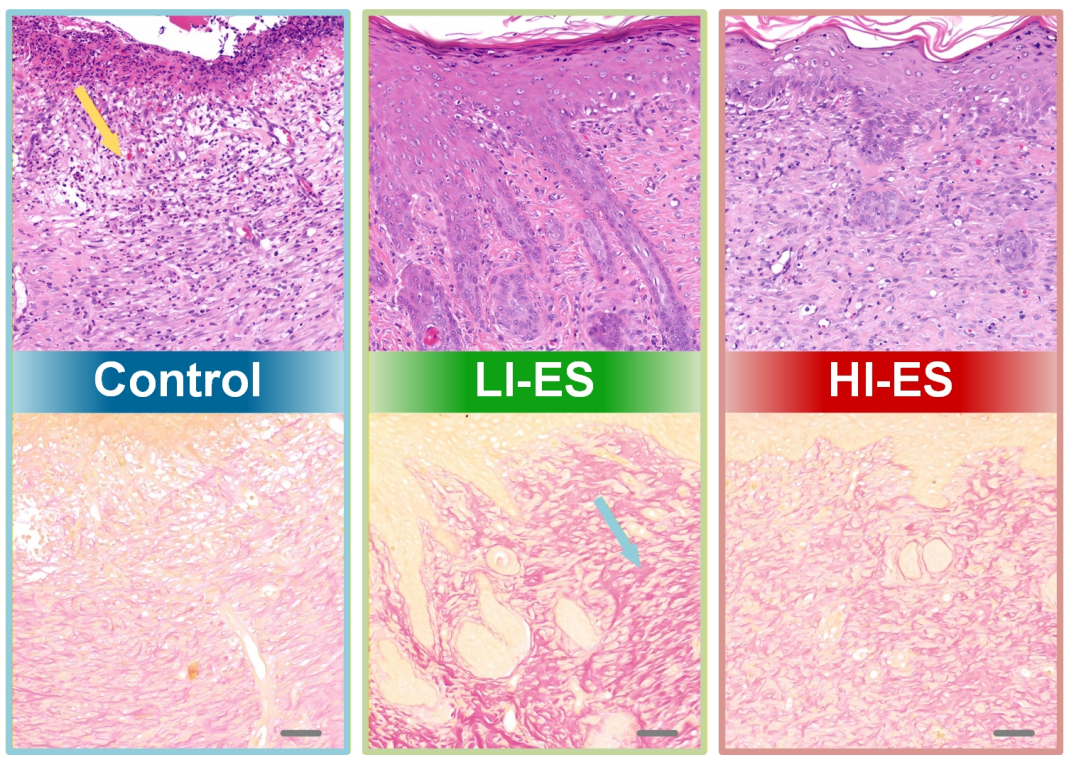

Supplementary Fig. 13. Representative histological images on day 11. Upper panels show H&E staining, and lower panels show Van Gieson staining. Yellow arrow indicates inflammatory cells, and blue arrow indicates collagen fibers. Scale bars = 50 μm.

X. Comparison of RF-to-DC Conversion Efficiency

To provide a quantitative benchmark for the power conversion performance of the acoustic-driven current source (based on the IDT delay line configuration characterized in the manuscript), we compare its output DC power P_{out} versus input RF power P_{in} with state-of-the-art RF-to-DC rectifiers from the literature. Supplementary Fig. 14 presents this comparison on a double-logarithmic scale. It is worth noting that the output power can be further enhanced by employing multi-IDT arrays or SPUDT structures, as demonstrated in the manuscript.

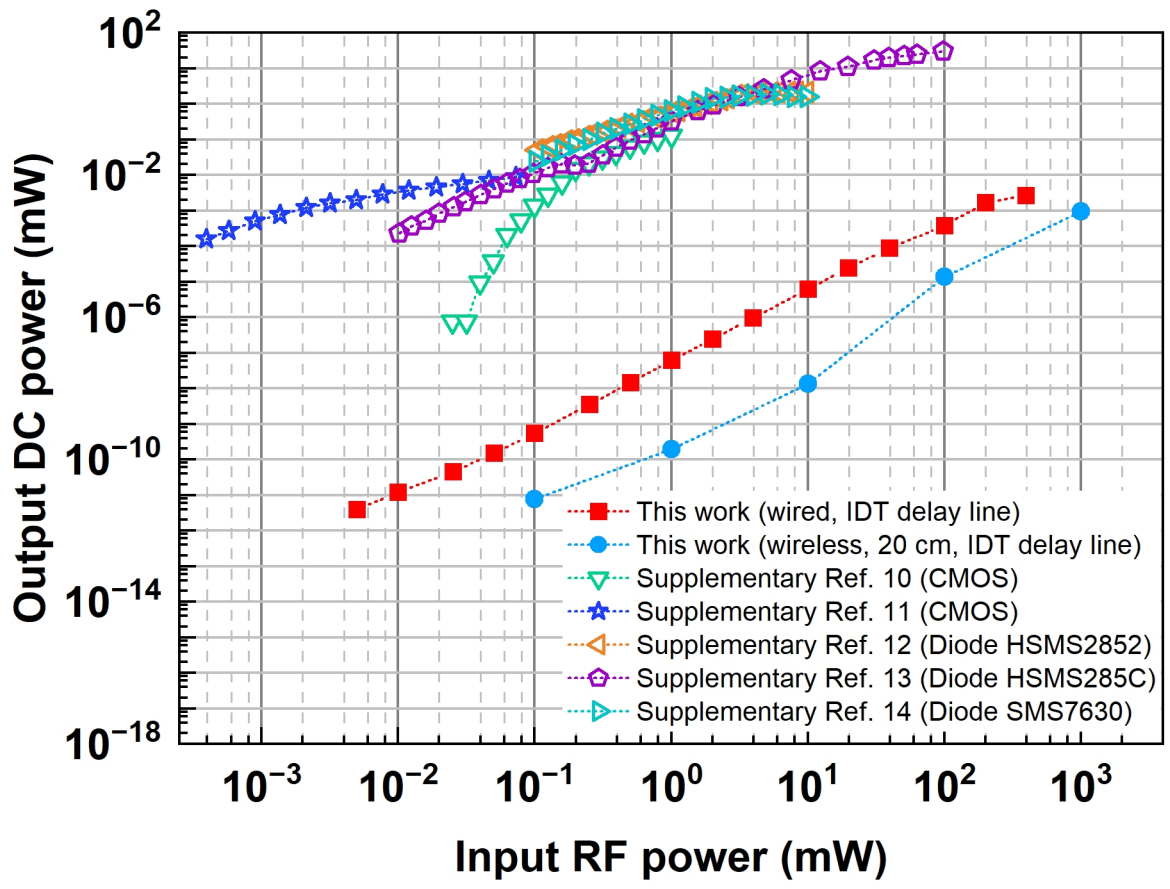

Supplementary Fig. 14. Output DC power P_{out} versus input RF power P_{in}. The acoustic-driven current source (wired operation, and wireless operation at a transmission distance of 20 cm, based on the IDT delay line configuration) is compared with state-of-the-art RF-to-DC rectifiers[10–14]. These include CMOS implementations, such as those by Chouhan[10] and Wang[11]. In addition, discrete Schottky-diode-based implementations include those by Abdelaal[12], Tariq[13], and Saini[14]. For the acoustic-driven current source, P_{out} is calculated under the maximum power transfer condition ($R_L = R_{out}$) with $R_{out} \approx 10.5$ kΩ. Source data are provided as a Source Data file.

For the acoustic-driven current source, the output DC power under maximum power transfer condition is calculated as $P_{out} = I_{AE}^2 R_{out}/4$, where I_{AE} is the measured acoustoelectric current (from wired operation in Fig. 3 and wireless operation at a transmission distance of 20 cm in Fig. 5 of the manuscript) and $R_{out} \approx 10.5$ kΩ is the DC output impedance (Supplementary Fig. 7). For the literature data, $P_{out} = V_{DC}^2/R_L$ is calculated from the reported output DC voltage V_{DC} and load resistance R_L.

The selected references represent state-of-the-art RF-to-DC converters across two primary technology routes: CMOS integrated implementations and discrete Schottky-diode-based implementations. The CMOS route includes a 0.18 μm CMOS differential-drive voltage multiplier at 433 MHz[10] and a 0.18 μm CMOS all-nMOS rectifier with internal threshold-voltage cancellation at 2.4 GHz[11]. The discrete Schottky-diode route includes a voltage-doubler topology using HSMS-2852 diodes at 0.9/2.46 GHz[12], a multi-stage Cockcroft–Walton voltage multiplier using HSMS-285C diodes at 0.87–2.5 GHz[13], and a voltage-doubler configuration with a modified Pi-network using an SMS7630 diode at 1.7–2.7 GHz[14].

As shown in Supplementary Fig. 14, the acoustic-driven current source exhibits a linear $P_{out}-P_{in}$ relationship over approximately four orders of magnitude in wired operation. However, its efficiency is several orders of magnitude lower than that of state-of-the-art RF rectifiers.

These results underscore that, in its current form, the acoustoelectric approach is less efficient than conventional rectifiers, and significant materials and device improvements are required for practical wireless power delivery.

XI. The Effect of Annealing Process on $LiNbO_3$ Substrates

The annealing was carried out with a heating rate of 5 °C·min^{-1}, a target temperature of 500 °C, a soaking time of 1 hour, and furnace cooling. To evaluate its effect on the piezoelectric properties, the longitudinal piezoelectric coefficient d_{33} was measured at five different locations on the 128°YX $LiNbO_3$ substrate using a room-temperature d_{33} meter (ZJ-3, Institute of Acoustics, Chinese Academy of Sciences). The measurement results are shown in Supplementary Table 3. The average d_{33} was increased slightly from 19.92 pC·N^{-1} to 20.42 pC·N^{-1}, a difference of only 2.5%, indicating that the annealing process had a negligible impact on the piezoelectric performance of the substrate.

Supplementary Table 3. Measured d_{33} values of 128°YX $LiNbO_3$ before and after annealing (unit: pC·N^{-1})

Sample	1	2	3	4	5	Average
Before annealing	19.9	19.9	19.9	20.0	19.9	19.92
After annealing	20.4	20.4	20.5	20.4	20.4	20.42

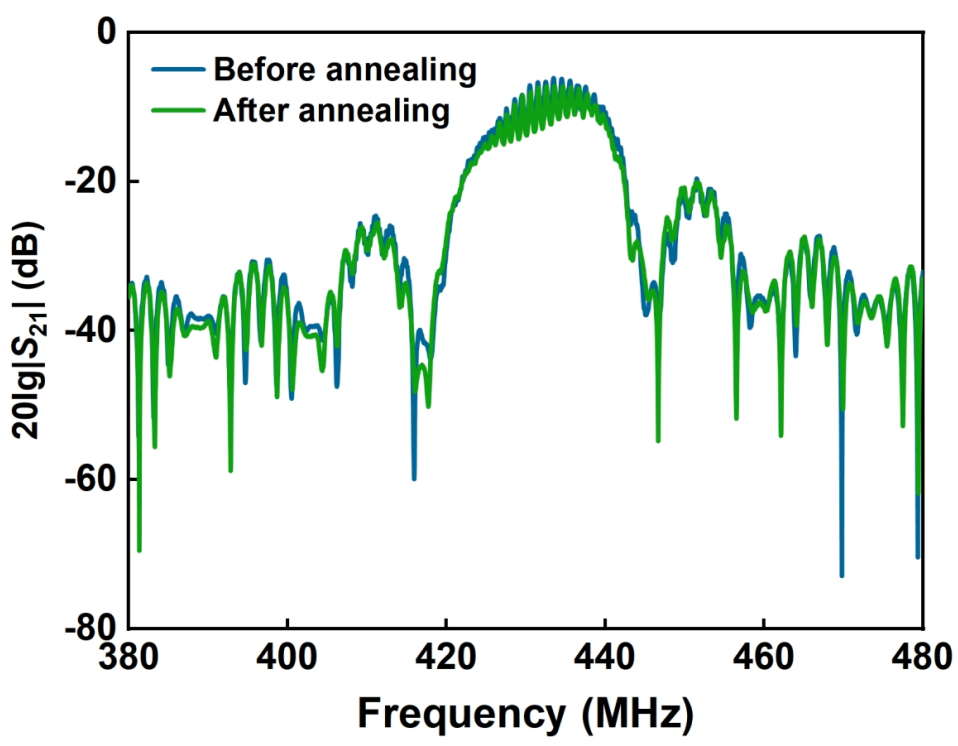

Supplementary Fig. 15. Frequency response characteristics of the SAW device before and after annealing. Source data are provided as a Source Data file.

To evaluate potential changes in the frequency response characteristics of the SAW device, this paper fabricated devices directly on the LN substrate without any thin-film deposition in the acoustic propagation region and measured their performance before and after annealing, as shown in Supplementary Fig. 15. The insertion loss was increased slightly from 6.12 dB to 7.18 dB, and the center frequency was shifted from

433.48 MHz to 433.60 MHz. These minimal changes indicate that the annealing process showed negligible impact on the device's frequency response characteristics.

In summary, the experimental results demonstrated that annealing at 500 °C for 1 hour did not cause any significant changes in the piezoelectric polarization characteristics of the 128°YX $LiNbO_3$ substrate or in the frequency responses of the corresponding SAW device.

XII. The Acoustic-Driven Current Source Measurement System

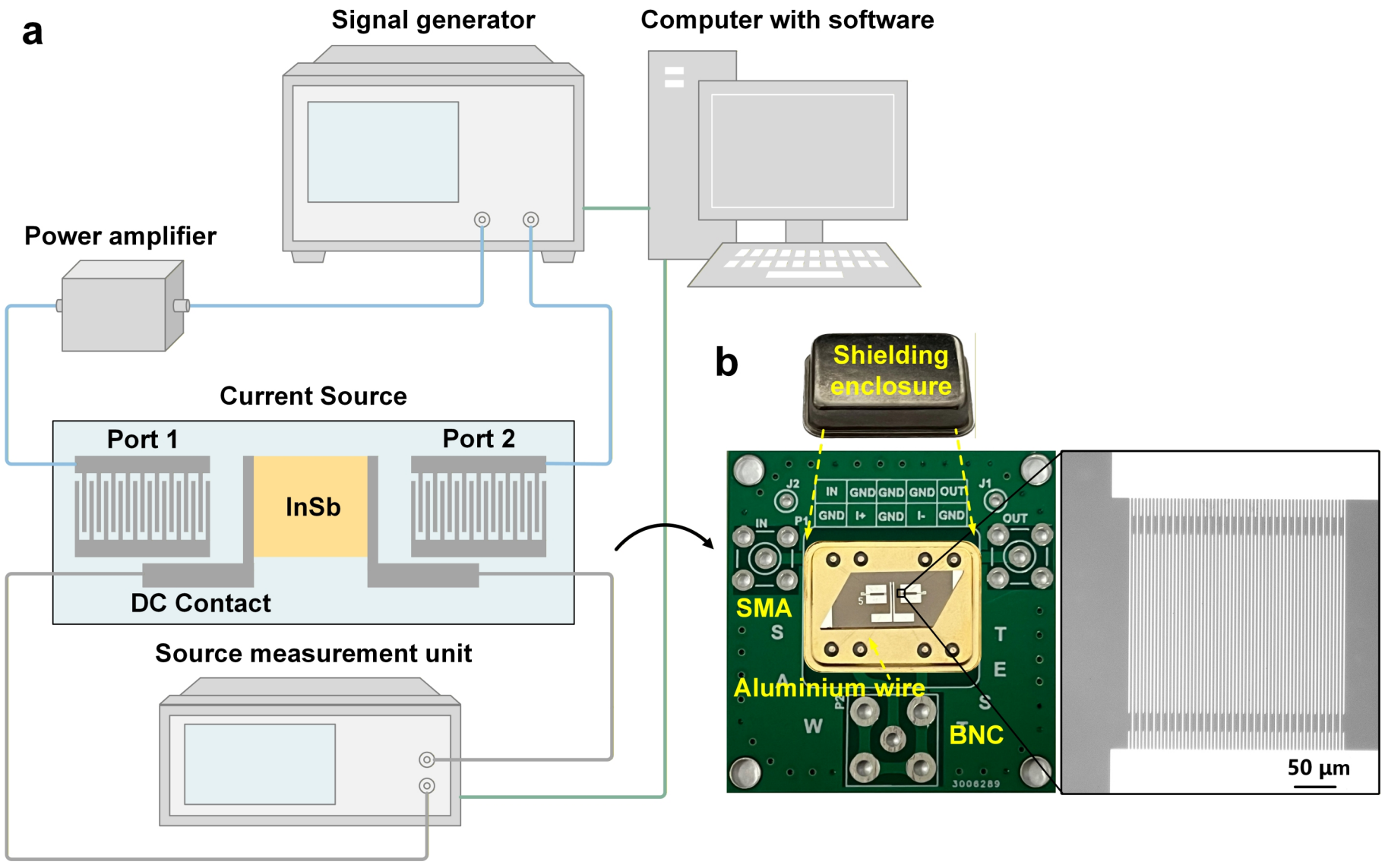

Supplementary Fig. 16. Acoustic-driven current source. **a** A schematic illustration of the measurement system. **b** Optical micrographs of the fabricated device and the specially designed PCB.

XIII. Validation of Isolated Grounding Configuration

To ensure accurate AE current measurements and to eliminate errors caused by ground loops, an isolated grounding scheme was adopted, in which the ground of the source measurement unit was electrically isolated from that of the acoustic-driven current source. The following validations were conducted:

1. Connection Method

All the current measurements employed a four-wire Kelvin connection with a Keithley 2450 source measurement unit, ensuring high precision by minimizing contact resistance effects.

2. Instrument Noise Test

The background current of the source measurement unit (SMU) under open-circuit conditions was measured to be below 5 pA, corresponding to the instrument's noise floor (Supplementary Fig. 17).

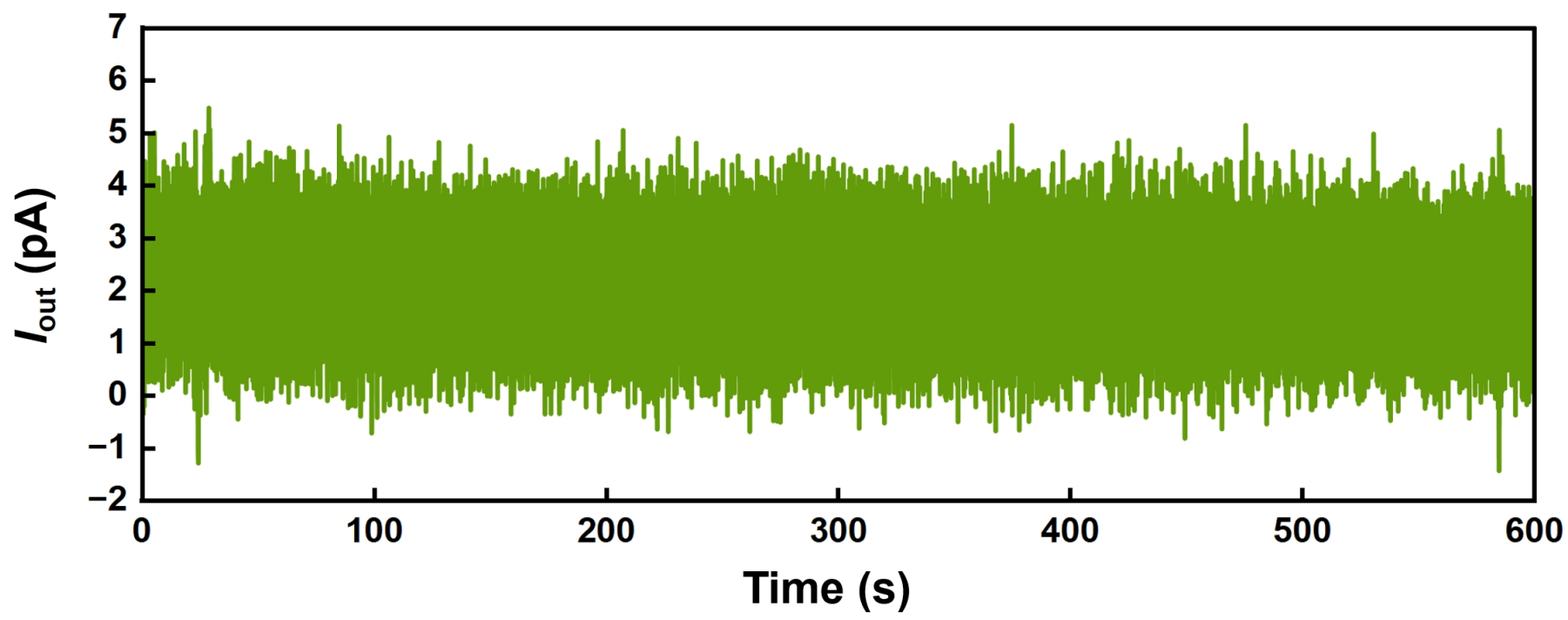

Supplementary Fig. 17. Background current of the SMU under open-circuit conditions. Source data are provided as a Source Data file.

3. Reverse Current Validation

To assess whether the ground potential differences could introduce reverse currents, a second Keithley 2450 source measurement unit was employed. The current between the ground terminal of the source measurement unit in the acoustic-driven current source measurement system (GSMU) and the common ground was first measured. Then, the GSMU was connected to the ground of the acoustic-driven current source (GADCS), and the measurement was repeated. As shown in the Supplementary Fig. 18, the current between GSMU and the common ground was negligible. However,

when the GSMU was connected to the GADCS, a reverse current of approximately 40 pA was detected, caused by a ground potential difference. This reverse current accounts for only 3.28% of the minimum output current of the acoustic-driven current source (1.22 nA), and thus shows a negligible effect on the measurement accuracy.

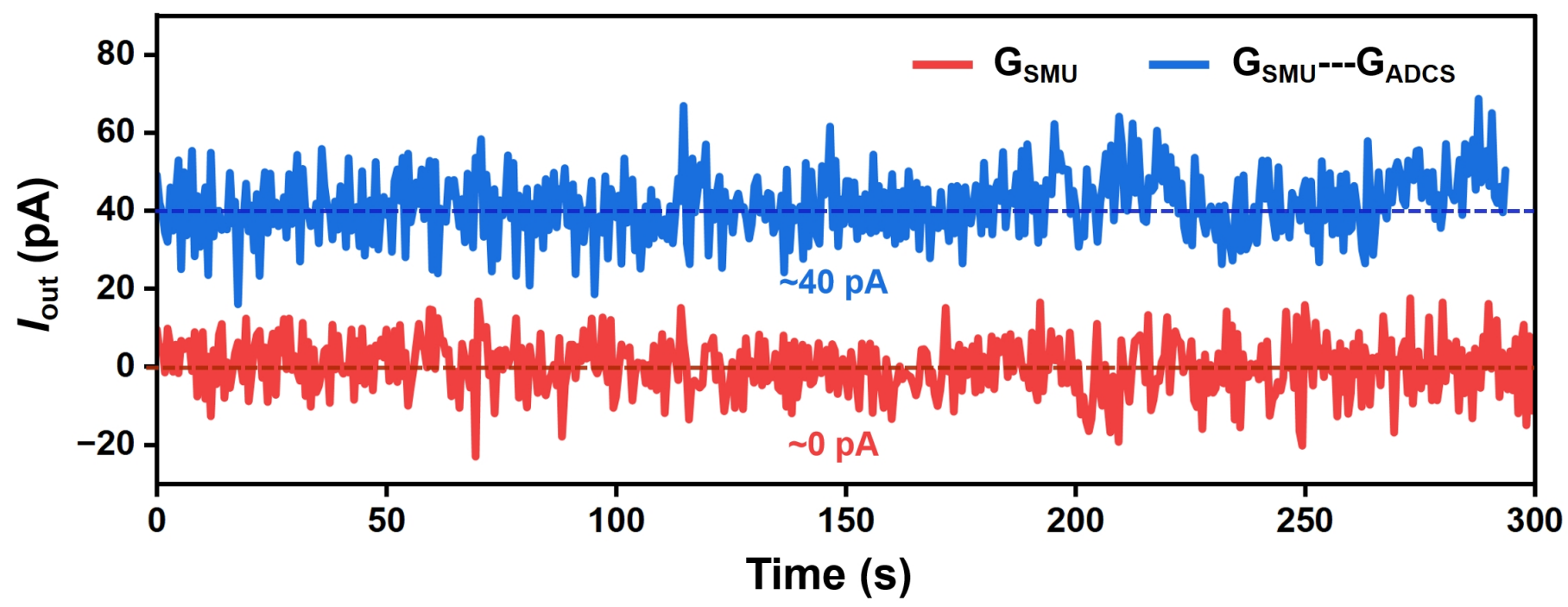

Supplementary Fig. 18. Reverse current induced by ground potential difference. Source data are provided as a Source Data file.

These results confirm that the isolated grounding effectively suppresses ground-loop induced errors and ensures the reliability of the acoustic-driven current source measurements.

XIV. Characterization of Acoustoelectric Current Response Time

The acoustoelectric effect is fundamentally governed by phonon-electron momentum transfer, with a theoretical dynamic response time typically on the order of microseconds or shorter. To characterize the transient responses of the output current, a high-frequency transimpedance amplifier (ADI, LTC6268) and a high-speed, broadband oscilloscope (Siglent, SDS6104 H10 Pro) were employed to capture the device's responses under the pulsed excitation. The RF signal generator (SRS, SG394) was configured in a pulse mode, featuring a pulse period of 500 μs, a pulse width of 5 μs, and an output power of 14 dBm. The transimpedance amplifier provided a gain of 10^4 V·A^{-1}, which was sufficient to effectively convert the μA-level currents into mV-level voltages measurable by the oscilloscope.

The typical time-domain waveform is presented in Supplementary Fig. 19. The measured intrinsic response time is less than 1 μs, defined as the interval from the onset of the RF pulse (C1) to the point where the response signal reaches a steady-state flat top (C2).

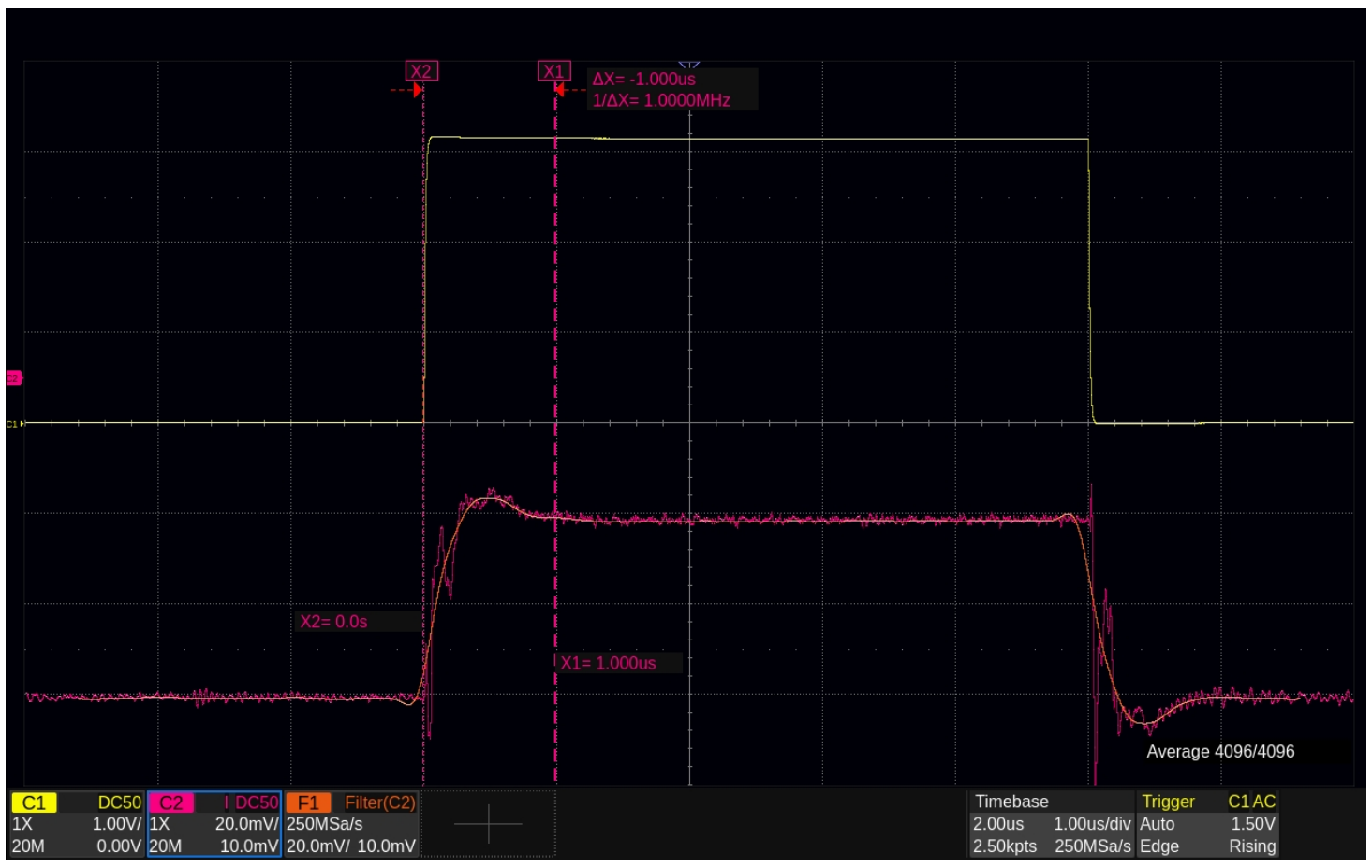

Supplementary Fig. 19. Transient response measurement. The yellow curve (C1) represents the RF pulse excitation, the pink curve (C2) denotes the output response, and the orange curve (F1) shows the filtered signal envelope. Source data are provided as a Source Data file.

It is worth noting that the initial ringing observed on the rising and falling edges of the raw signal (C2) is a known artifact originating from the inherent underdamped response and parasitic LC resonance of the high-frequency transimpedance amplifier circuit, when processing ultra-fast, weak current edges. This oscillation rapidly

attenuates within 1 μs. By applying a 2 MHz digital low-pass filter to the raw data, the resulting envelope (F1) closely resembles a smooth, ideal step response.

XV. AC Current Source Test Results

To evaluate whether the proposed device can be applied as an AC current source, this paper conducted experiments using amplitude-modulated RF signals and examined the resulting output currents I_{out}. The experimental results confirmed that I_{out} exhibits a stable AC signal under amplitude modulation frequencies ranging from 1 Hz to 50 kHz, indicating that the device can be applied as an AC current source with a 3 dB bandwidth significantly greater than 50 kHz.

In the experiments, the RF signal generator (SRS, SG394) was set to have an output frequency of 435 MHz and an output power of 10 dBm. The amplitude modulation functions included sine, sawtooth, triangle, and square waves, with modulation frequencies ranging from 1 Hz to 50 kHz and a modulation depth of 90%.

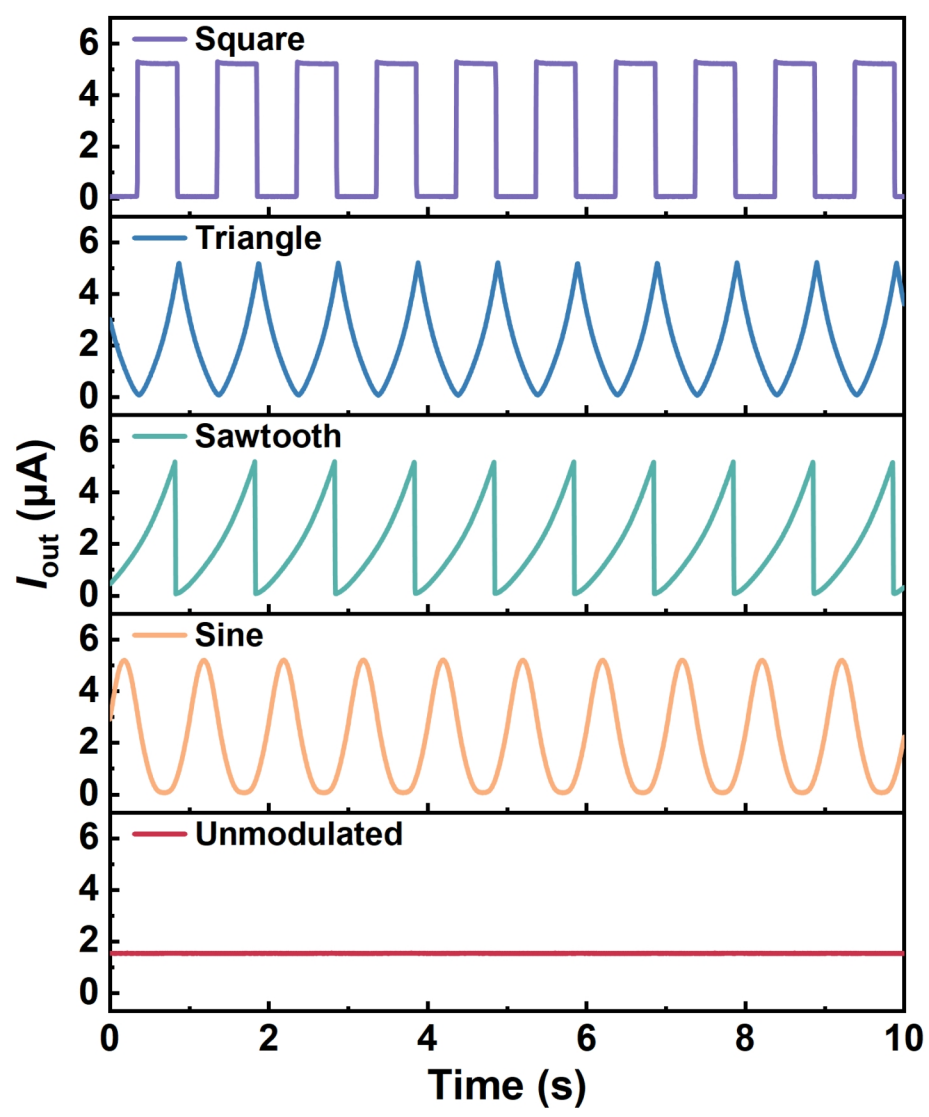

Supplementary Fig. 20. Periodic fluctuation of I_{out} over time under different amplitude modulation functions at a modulation frequency of 1 Hz. Source data are provided as a Source Data file.

When the RF input was amplitude-modulated at 1 Hz (Supplementary Fig. 20), the source measurement unit (Keithley, 2450) revealed a clear periodic AC variation of I_{out} following the modulation signal. For the sinusoidal modulation waveform, the root-mean-square (RMS) value of the AC component of I_{out} was approximately 1.8 μA, calculated from the peak-to-peak amplitude of the corresponding current waveform. In

contrast, the unmodulated DC output current had an average value of approximately 1.5 μA. The approximately 1.8 μA RMS value refers specifically to the sinusoidal modulation waveform and was not calculated for the triangular, sawtooth, or square-wave modulation waveforms.

As shown in Supplementary Fig. 21, with a sine wave as the amplitude modulation function, when the modulation frequency was gradually increased to 1 kHz, the periodic fluctuation of I_{out} could still be observed, but the recorded waveform became severely distorted, limited by the theoretical maximum sampling rate of 5000 samples·s^{-1} of the source measurement unit.

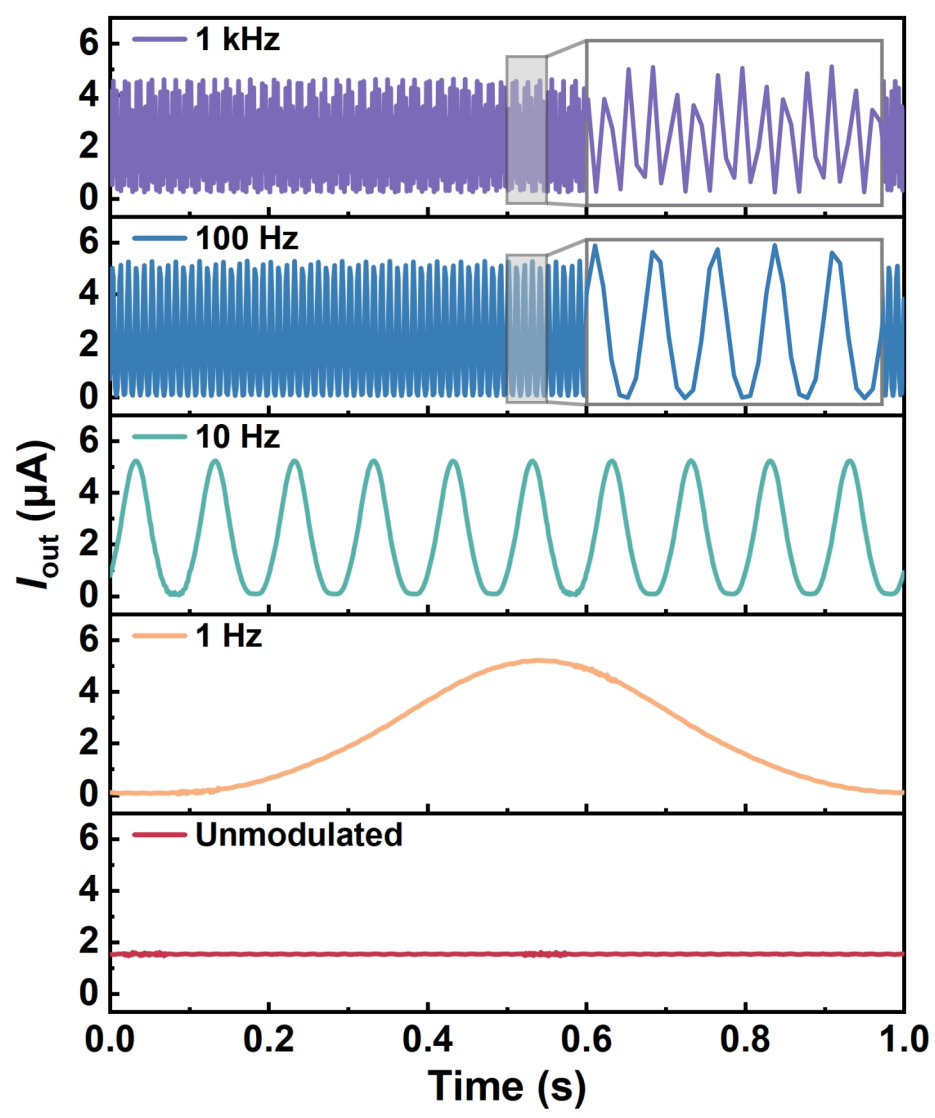

Supplementary Fig. 21. Periodic fluctuation of I_{out} over time under different modulation frequencies with a sine-wave amplitude modulation function. Source data are provided as a Source Data file.

To further verify the AC response characteristics of the device in the high-frequency region and characterize its 3 dB bandwidth, the device output was connected via a 10 dB attenuator to a spectrum analyzer (RIGOL, RSA3030E-TG). As shown in Supplementary Fig. 22, the spectral observation range was set from 9 kHz to 55 kHz, considering the lower frequency limit of the spectrum analyzer is 9 kHz and the maximum amplitude modulation frequency of the RF signal generator (SRS, SG394) is 50 kHz. A sine wave was used as the modulation function, with the modulation

frequency set sequentially to be 10 kHz, 20 kHz, 30 kHz, 40 kHz, and 50 kHz. The response curves exhibited the corresponding peaks at each frequency. Within the range of 10 kHz to 50 kHz, the fluctuation in its peak power was no greater than 0.2 dBm, indicating that the device maintained a good AC response capability throughout this range, and its 3 dB bandwidth is significantly larger than 50 kHz.

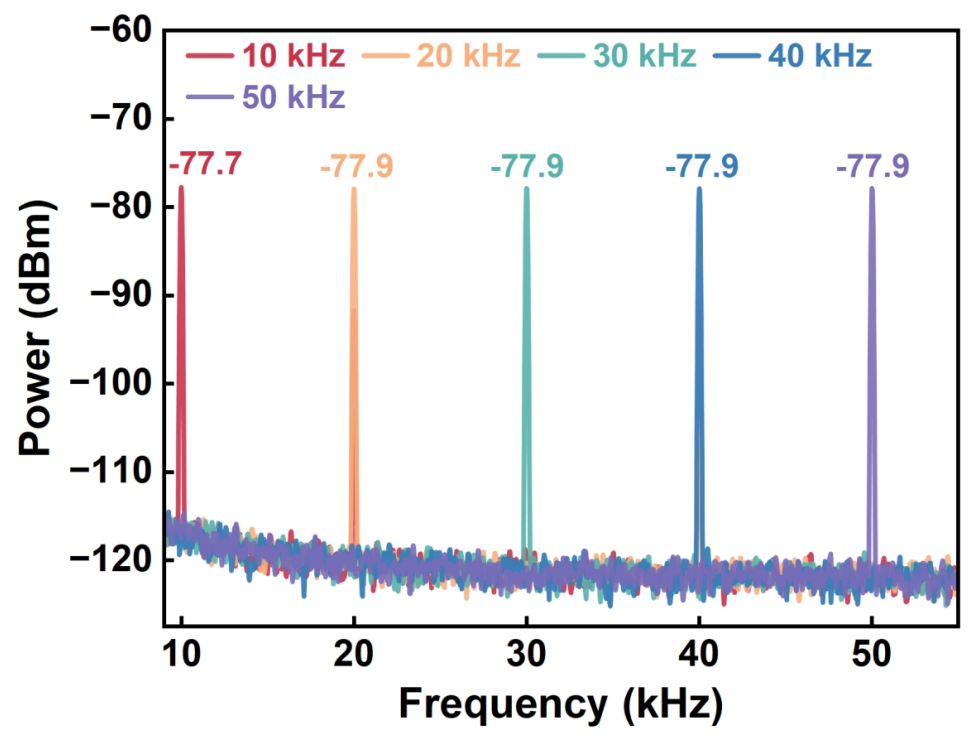

Supplementary Fig. 22. Spectral response characteristics of I_{out} under different modulation frequencies (10 kHz, 20 kHz, 30 kHz, 40 kHz, and 50 kHz). Source data are provided as a Source Data file.

References

1 Schwalbe, C., H. Lars Vegard: key communicator and pioneer crystallographer. *Crystallography Reviews* **20**, 9-24 (2013).

2 Coldren, L., A. Trapping model for InSb thin films. *Applied Physics Letters* **18**, 319-321 (1971).

3 Yamanouchi, K. & Shibayama, K. Monolithic elastic surface wave amplifiers of continuous operation. *Japanese Journal of Applied Physics* **16**, 493-496 (1977).

4 Yamanouchi, K. et al. Elastic surface wave functional device and electronic circuit using the element. European Patent EP0810726 (2005).

5 Abbott, B. P. A coupling-of-modes model for SAW transducers with arbitrary reflectivity weighting, University of Central Florida (1989).

6 Yuan, Y., Yang, T., Chen, X. et al. Analysis of the acoustoelectric response of SAW gas sensors using a COM model. *Microsystems & Nanoengineering* **10**, 69 (2024).

7 Wu, S., Chen, Y. C. & Chang, Y. S. Characterization of AlN films on Y-128 $LiNbO_3$ by surface acoustic wave measurement. *Japanese Journal of Applied Physics* **41**, 4605-4608 (2002).

8 Hrostowski, H. J., Morin, F. J., Geballe, T. H. & Wheatley, G. H. Hall effect and conductivity of InSb. *Physical Review* **100**, 1672-1676 (1955).

9 Wang, Y. *et al*. Electrical Microneedles for Wound Treatment. *Advanced Science* **12**, 2409519 (2025).

10 Chouhan, S. S., Nurmi, M., & Halonen, K. Efficiency enhanced voltage multiplier circuit for RF energy harvesting. *Microelectronics journal* **48**, 95-102 (2016).

11 Wang, G., Li, X. & Chen, Z. A 2.4-GHz −33-dBm sensitivity battery-free RF energy harvesting system with 17-dB input power range. *IEEE Trans. Circuits Syst. II Exp. Briefs* **73**, 1120–1124 (2026).

12 Abdelaal, M. , Elsayed, M. , & Mansour, I. . Design of High-Efficiency RF Energy Harvesting Rectifier Circuit. In *2026 43rd National Radio Science Conference (NRSC)*, IEEE (2026).

13 Tariq, B. et al. Design and analysis of a dual-port multiband RF-to-DC converter for IoT energy harvesting applications. *PLoS One* **20**, e0321729 (2025).

14 Saini, P., Malhotra, S. & Gupta, L. A compact and wideband rectifier for RF energy harvesting with robust performance across varying input power and load range. *Phys. Scr.* **100**, 125026 (2025).
