# Supplementary material for: Wireless acoustic-driven current source achieving nanoampere-level at millimeter-scale
Source: Nat Commun. 2026 Sep 28;17:11181. doi: 10.1038/s41467-026-78004-y (PMC13714251; doi:10.1038/s41467-026-78004-y)
Supplement: Supplementary file 2 [file 41467_2026_78004_MOESM2_ESM.pdf]

Corresponding author(s): Prof. Wei Luo

Last updated by author(s): Jul 27, 2026

Reporting Summary

Nature Portfolio wishes to improve the reproducibility of the work that we publish. This form provides structure for consistency and transparency in reporting. For further information on Nature Portfolio policies, see our Editorial Policies and the Editorial Policy Checklist.

Statistics

For all statistical analyses, confirm that the following items are present in the figure legend, table legend, main text, or Methods section.

n/a	Confirmed	
☐	☒	The exact sample size (n) for each experimental group/condition, given as a discrete number and unit of measurement
☐	☒	A statement on whether measurements were taken from distinct samples or whether the same sample was measured repeatedly
☐	☒	The statistical test(s) used AND whether they are one- or two-sided<br>*Only common tests should be described solely by name; describe more complex techniques in the Methods section.*
☒	☐	A description of all covariates tested
☐	☒	A description of any assumptions or corrections, such as tests of normality and adjustment for multiple comparisons
☐	☒	A full description of the statistical parameters including central tendency (e.g. means) or other basic estimates (e.g. regression coefficient) AND variation (e.g. standard deviation) or associated estimates of uncertainty (e.g. confidence intervals)
☒	☐	For null hypothesis testing, the test statistic (e.g. F, t, r) with confidence intervals, effect sizes, degrees of freedom and P value noted<br>*Give P values as exact values whenever suitable.*
☒	☐	For Bayesian analysis, information on the choice of priors and Markov chain Monte Carlo settings
☒	☐	For hierarchical and complex designs, identification of the appropriate level for tests and full reporting of outcomes
☒	☐	Estimates of effect sizes (e.g. Cohen's d, Pearson's r), indicating how they were calculated

Our web collection on statistics for biologists contains articles on many of the points above.

Software and code

Policy information about availability of computer code

Data collection	EasyZoom 5 3D (digital microscope, for device morphology imaging); Keithley 2450 (SourceMeter, for current/voltage recordings); Agilent E5071C (network analyzer, for S-parameter measurements); ZEISS GeminiSEM 300 (scanning electron microscope, for SEM imaging); ECOPLIA HMS-5500 (Hall effect measurement system, for carrier concentration and mobility measurements); PANalytical B.V. X'Pert PRO (X-ray diffractometer, for XRD patterns); Wayne Kerr 6500B (impedance analyzer, for impedance-frequency measurements); SRS SG394 (RF signal generator, for RF excitation); Siglent SDS6104 H10 Pro (digital oscilloscope, for response measurements); Pannoramic MIDI (high-resolution slide scanning system, 3DHISTECH Ltd., Budapest, Hungary, for brightfield whole-slide imaging of H&E and Van Gieson-stained skin sections); iQOO Neo5 SE (smartphone camera, for rat wound photography).
Data analysis	COMSOL Multiphysics, ImageJ, MATLAB and GraphPad Prism

For manuscripts utilizing custom algorithms or software that are central to the research but not yet described in published literature, software must be made available to editors and reviewers. We strongly encourage code deposition in a community repository (e.g. GitHub). See the Nature Portfolio guidelines for submitting code & software for further information.

Data

Policy information about availability of data

All manuscripts must include a data availability statement. This statement should provide the following information, where applicable:

- Accession codes, unique identifiers, or web links for publicly available datasets
- A description of any restrictions on data availability
- For clinical datasets or third party data, please ensure that the statement adheres to our policy

All data supporting the findings of this study are available within the paper and its Supplementary Information. Source data are provided with this paper as a Source Data file (Excel format). No accession codes or restrictions apply.

Research involving human participants, their data, or biological material

Policy information about studies with human participants or human data. See also policy information about sex, gender (identity/presentation), and sexual orientation and race, ethnicity and racism.

Reporting on sex and gender	n/a
Reporting on race, ethnicity, or other socially relevant groupings	n/a
Population characteristics	n/a
Recruitment	n/a
Ethics oversight	n/a

Note that full information on the approval of the study protocol must also be provided in the manuscript.

Field-specific reporting

Please select the one below that is the best fit for your research. If you are not sure, read the appropriate sections before making your selection.

☒ Life sciences ☐ Behavioural & social sciences ☐ Ecological, evolutionary & environmental sciences

For a reference copy of the document with all sections, see nature.com/documents/nr-reporting-summary-flat.pdf

Life sciences study design

All studies must disclose on these points even when the disclosure is negative.

Sample size	Twenty-four male Sprague-Dawley rats were randomly divided into three groups (n = 8 per group). The sample size was selected based on previous preclinical wound healing studies and ethical considerations to minimize animal use.
Data exclusions	No data were excluded from the analysis.
Replication	The wound healing experiment was performed on distinct biological samples (n = 8 rats per group). Temperature-dependent current measurements were repeated over four cycles at each temperature. All replication attempts were successful
Randomization	Animals were randomly divided into three groups (control, LI-ES, and HI-ES) using a random number table.
Blinding	The investigators performing wound area measurements (ImageJ analysis) and histological evaluations were blinded to the group allocation. However, due to the nature of the electrical stimulation setup, the operators applying the stimulation could not be blinded

Reporting for specific materials, systems and methods

We require information from authors about some types of materials, experimental systems and methods used in many studies. Here, indicate whether each material, system or method listed is relevant to your study. If you are not sure if a list item applies to your research, read the appropriate section before selecting a response.

Materials & experimental systems

n/a	Involved in the study
☒	☐ Antibodies
☒	☐ Eukaryotic cell lines
☒	☐ Palaeontology and archaeology
☐	☒ Animals and other organisms
☒	☐ Clinical data
☒	☐ Dual use research of concern
☒	☐ Plants

Methods

n/a	Involved in the study
☒	☐ ChIP-seq
☒	☐ Flow cytometry
☒	☐ MRI-based neuroimaging

Animals and other research organisms

Policy information about studies involving animals; ARRIVE guidelines recommended for reporting animal research, and Sex and Gender in Research

Laboratory animals	Male Sprague-Dawley rats (6–7 weeks, 180–200 g, n = 24) were used.
Wild animals	No wild animals were used in this study.
Reporting on sex	Only male rats were used.
Field-collected samples	No field-collected samples were involved.
Ethics oversight	All procedures were approved by the Animal Ethics Committee of Wuhan Myhalic Biotechnology Co., Ltd. (approval No. HLK-20250805-001).

Note that full information on the approval of the study protocol must also be provided in the manuscript.

Plants

Seed stocks	n/a
Novel plant genotypes	n/a
Authentication	n/a
