# Supplementary material for: Wireless acoustic-driven current source achieving nanoampere-level at millimeter-scale
Source: Nat Commun. 2026 Sep 28;17:11181. doi: 10.1038/s41467-026-78004-y (PMC13714251; doi:10.1038/s41467-026-78004-y)
Supplement: Supplementary file 3 [file 41467_2026_78004_MOESM3_ESM.pdf]

nature portfolio

Peer Review File

Wireless Acoustic-Driven Current Source Achieving Nanoampere-Level at Millimeter-Scale

Corresponding Author: Professor Wei Luo

This file contains all reviewer reports in order by version, followed by all author rebuttals in order by version.

Version 0:

Reviewer comments:

Reviewer #1

(Remarks to the Author)
This article presents an acoustic-driven current source based on the acoustoelectric effect between surface acoustic waves (SAW) and semiconductors. The study demonstrates a broad output current range from the nanoampere to microampere scale, with detailed performance evaluations. However, the manuscript primarily focuses on showcasing the favorable performance metrics without providing sufficient scientific investigation into the underlying mechanisms responsible for these results. This lack of fundamental analysis diminishes the study's scientific significance. Furthermore, the manuscript does not present any practical applications that align with the reported performance, limiting its impact and broader relevance. Given these shortcomings, I can NOT give the study my recommendation for publication in Nature Communications in its current form. More detailed comments are listed below.
1. On page 5, the authors mentioned that ‘There is a lattice mismatch between LiNbO3 and InSb, and oxygen diffusion from LiNbO3 into InSb leads to a degradation in the performance. Therefore, an In0.6Al0.4Sb buffer layer was inserted between InSb and the substrate”. However, the manuscript lacks a thorough discussion on the role of the buffer layer. Why the buffer layer can solve the problem of lattice mismatch and oxygen diffusion? What are the electrical property differences between the devices with and without the buffer layer?
2. What is the thickness of the buffer layer? How were the thickness values for both the buffer layer and the 250 nm InSb film determined? How was the atomic percentage of In and Al selected? Would variations in layer thickness or atomic composition affect the electrical properties of the device? A discussion or experimental validation is suggested to be added.
3. On page 11, in Fig 4a, the authors claimed “the current accuracy was remained below 3%, exhibiting an increasing trend with the rise of Iset”. However, the data in Figure 4a shows that Iacc achieved its lowest value at Iset=100 nA. Please clarify and provide an explanation for this phenomenon.
4. The manuscript repeatedly highlights the need for miniaturized current sources. However, the exact size of the reported device is not explicitly stated. Please provide quantitative size specifications of this current source.
5. The authors suggest that this device could be useful for sensor networks and implantable medical devices. However, there is no detailed discussion on specific application scenarios that require a current source with an output as low as the nanoampere range. Furthermore, considering that the reported wireless transmission distance is less than 60 cm, is this sufficient for practical use in implantable devices? An experimental demonstration is suggested to be added.
6. The manuscript contains several errors that should be carefully checked. Examples include: on page 8 line 158, “In Figure 3b” should be “In Figure 2b”; on page 11 line 236, sampling rate missed the symbol.

Reviewer #2

(Remarks to the Author)
This work is of good interest and present an original method based on acoustic-driven current source and allowing to achieves nanoamp-level output currents surpassing the most commercially voltage-controlled microcurrent chips limited to microamp-level. Stydied methode a direct measurements of dynamic electrokinetic properties of electrical double layers (EDL) using Surface acoustic wave excitation. The method also has the advantage of enabling wireless control of the current induced by a SAW device via an RF link. The overall quality of the paper and the results presented therein are of a good quality and deserve to be published. Major revisions are therefore required to enhance the quality of the paper to match the standard of Nature communication Journal.
1- Page 6, Line 135: It is stated that The AE current is directly proportional to the $Keff2$ and inversely proportional to the

square of vs. However, in formula (1), the frequency (f=Vs/lamda) appears in the numerator. It is therefore more correct to say that AE current is inversely proportional to Vs and not to the square of Vs. It is more intuitive to talk about the wavelength (Lamda) since the greater Lamda is, the more the wave penetrates the substrate and less the surface, resulting in a weaker current.
2- Figure 2 (b) : It is not clear whether this is a simulation or an experimental measurement.
3- Please explain how the losses in the InSb layer are modelled and what physical parameters are used to carry out the simulation.
4- Figure 5 (a): For the wired configuration, we understand the advantage of using a double port SAW device (delay line) to allow S21 characterization. However, in a wireless configuration this advantage disappears and a single IDT connected to the antenna is sufficient. It may be appropriate to connect both IDTs to the antenna to generate a standing wave, in which case the position of the electrodes on the InSb layer needs to be modified.
5- It should also be pointed out that half of the RF energy converted into an elastic surface wave is lost due to the propagation of the wave in both directions. A unidirectional IDT will be relevant.
6- Authors claim that : “The acoustic-driven current source exhibits an excellent linear relationship between output current and input RF power, with a high correlation”. This is thru in wired configuration. This is of little interest for implanted devices. In wireless configuration, linearity is less good and will depend on several parameters such as distance, noise from surrounding reflections and antenna orientation. In real-life operation, linearity will be even more degraded than shown in Figure 5-e. Thank you for your comments.
7- For wireless configuration, please specify the state of the art of other technologies such as magnetic induction or capacitive coupling allowing control of the electric current.

Reviewer #3

(Remarks to the Author)
The manuscript by Y. Yuan et al titled “Wireless Acoustic-Driven Current Source Achieving Nanoampere-Level at Millimeter-Scale” demonstrates a continuously operated direct current (DC) source by heterogeneously integrating lithium niobate (LN) with semiconducting material InSb. The output current of such current sources can be controlled by modulating their input RF powers, showing a low current limit and a wide dynamic range. The work advances the heterogenous integration of LN with semiconducting materials, and showing interesting results with potential broad and high impacts. However, following technical comments need to be fully addressed before I could make a recommend (or not).
1. The similar device structures (elastic waves propagating in semiconductor and electro-acoustic effect) have been studied in recent publications, [Nature Communications 12, 2769 (2021)], [Nature Materials 23 (10), 1386-1393 (2024)] and [Nature Electronics 6 (1), 76-85 (2023)]. Beyond the previous work studied how current amplify acoustic waves, this work studies how acoustic waves generate current. Could authors highlight the advances by this work compared to previous work to give readers a more comprehensive background. For example, can you compare electron mobility and sheet carrier density with existing works? And what’s the future path towards a larger electron mobility in terms of material stacks?
2. What’s the condition of the current being measured? Is the two ends of semiconductor shorted? i.e. set output voltage to 0 in Keithley, 2450 when measuring the current.
3. Regarding Fig. 4(f), I failed to understand how an impedance analyzer being used to characterize the impedance in this case. It seems the authors are trying to claim the output impedance of their current source. If I understand correctly, impedance analyzer will generate signal at certain frequency and measures the voltage and current at given frequency over the device under test (DUT). Therefore, what measured is the impedance of the semiconductor at RF frequency, not the output impedance of current source. To characterize output impedance, authors should give the current-voltage (IV) curve of different loads (e.g. R=0, 10, 100, 1000 Ohm) connected to the source. Also what’s the open voltage of the source. Alternative, the Keithley, 2450 used by authors could also perform a IV measurement.
4. I feel the demonstrated power supply seems to be a voltage source, as the force on electrons given by acoustic waves will be balanced by the electric field cross the semiconductor. From bandstructure, the acoustic waves will deform the band and create a local electrical potential gradient. For sure, once the authors could provide I-V curve of output, we can easily tell if it is a current source or a voltage source.
5. How is noise floor in Fig. 4c calculated? More details are needed.
6. Why is 5 Hz chosen to analysis your noise density? Can you please also show the reader the full noise spectrum of your current source at different output level? Is there 1/f noise? If so, what’s the cut off frequency of 1/f noise.
7. Is the antenna an off-chip component? Is it possible to have it integrated on chip? Please articulate a technical solution to fully integrated current source using the proposed technique, if possible
8. It’s up to the author, but we are just very interested in if this design is also a good AC current source. For example, if you apply an amplitude modulation (e.g., 10Hz) on the RF signal (434.75MHz), how is the current output? Would you get a 10Hz current output at a similar level to the DC output if RF signal power level is the same? In another word, what is 3dB bandwidth of this AC current source?

Reviewer #4

(Remarks to the Author)
This manuscript presents the DC current induced at both ends of a 250 nm thick InSb film deposited on the cavity region of a two-port SAW delay line. The novelty lies in the observation of DC current through an Ag electrode on a previously well-known structure. Although the paper demonstrates the feasibility of a DC current source in a simple format, it lacks a

thorough explanation of the core aspects of the research.

- The RF sputtering of 250 nm thick InSb results in significant R-G center formation, surface defects, and interdiffusion, particularly in lightly-doped semiconductors. The manuscript should provide a more detailed explanation of the generation of electron-hole pairs, their movement induced by acoustic forces, their creation and recombination, and their flow toward metal barriers (need a schematic for explanation). Additionally, the paper should clarify why vibrational energy at RF 433 MHz is converted into DC current.

- The induced current between the electrodes is also influenced by the piezoelectric properties of the $LiNbO_3$ substrate. The manuscript should explain why current is generated only in the InSb layer (current will also be induced in the LN substrate) and describe the mechanisms involved.

- The current flow generates a new E-field at both ends of the InSb. The manuscript should explain how this newly generated E-field, which depends on the current magnitude, affects both the piezoelectric properties of the material and the DC current.

- More detailed information is needed about the type of InSb, its carrier concentration, carrier generation due to the acoustic wave, and the flow of carriers.

- The 500°C, 1-hour annealing process has a significant effect on the piezoelectric polarization characteristics of the LN substrate. A thorough explanation of this impact is required.

- DC current measurements are being performed with a current meter. The manuscript should clarify whether the experiment uses a common ground in the existing setup or a separate ground configuration. This clarification is essential for ensuring experimental accuracy. Additionally, it should confirm whether any reverse current is caused by the grounding of the test equipment.

- The XRD measurements appear to have been taken on a separate InSb film. Please clarify whether these measurements were taken on the multilayer structure on the actual LN substrate.

- Deposition of films thicker than 250 nm significantly affects the properties of the piezoelectric substrate, including SAW velocity, reflection, and electromechanical coefficients (K^2). It is crucial to explain how these effects were controlled during the experiment.

Reviewer #5

(Remarks to the Author)
I co-reviewed this manuscript with one of the reviewers who provided the listed reports. This is part of the Nature Communications initiative to facilitate training in peer review and to provide appropriate recognition for Early Career Researchers who co-review manuscripts.

Reviewer #6

(Remarks to the Author)
The authors present a variable amplitude current source based on the acoustoelectric (AE) effect. The current source takes in an RF input power at the 430 MHz input frequency of a Rayleigh SAW IDT and outputs a DC current whose amplitude is proportional to the RF input power. The AE device is constructed from a LiNbO3 SAW wafer with an InSb layer sputter deposited on top. An InAlSb buffer layer is included between the LiNbO3 and InSb layer to improve the InSb film quality. A high mobility of 1300 cm2/Vs is claimed for the InSb but it is unclear how it's measurement is isolated from the InAlSb layer. As is common in AE devices, the carrier concentration in the InSb layer is much too high, leading to a sheet conductivity that is two orders of magnitude above the optimum for peak AE efficiency. This contributes to a very poor RF to DC power conversion efficiency for the AE current source of 0.0001%. With such a poor conversion efficiency, it is no wonder that small controllable DC currents can be produced by rather large changes in RF power.
The authors insinuate that current sources in the nA to µA range are somehow difficult to realize using transistor circuits and that their device offers a unique ability to generate such currents precisely. However, such current sources are realized routinely in analog integrated circuits. Common examples include current source D/A converters and PTAT (proportional to absolute temperature) current sources, among many other. Typically these currents are used on chip to bias amplifier and other circuit blocks rather than made available as chip based current sources. However, there is nothing prohibiting the routing of such currents off of an integrated circuit.
A few specific comments for the authors to address to improve the manuscript are included below.

1) I'm not sure I agree that nano-amp level current sources cannot be generated using standard CMOS transistors techniques. This is done all the time in nanoWatt level integrated circuits highly published in the circuits research community. While such current sources may not be available commercially, I suspect this is due to the lack of a market for current sources with such small output levels as opposed to the inability of CMOS transistors to produce nA level current sources.
2) What is the sheet resistance or resistivity of the InAlSb layer? If this layer is conductive, what is the impact on the AE effect of placing it between the InSb and the LiNbO3?
3) What is the cause of the passband ripples in the AE current in figures 2 b and c? I would have thought this was triple

transit in (a) without the InSb but shouldn't the AE effect reduce the triple transit since it damps the SAW wave in this case?
4) Can the authors explicitly state that the output is a DC current from the input RF signal. The reviewer is afraid this point is not made explicitly clear and the RF plots in Fig. 2 may confuse some readers unfamiliar with the AE effect.
5) The current source has a very low RC filter characteristic that appears at 100 kHz. What is the source of the large capacitance causing such a low corner frequency?
6) At 10 mW RF input power, a current of approximately 2 microA is produced with a source impedance of approximately 10.5 kOhms. Under a maximum power transfer condition to the load, the amount of delivered power will be approximately 10 nW, making the efficiency 0.0001%. Can the authors comment on the utility of such an inefficient current source?
7) The output impedance of the current source of 10.5 kohms is quite low compared to transistor based cascode current sources. Can the authors comment on the errors induced by the low output resistance?
8) What is output voltage range over which the current source can operate?
9) The authors state "The output impedance of the acoustic-driven current source is slightly lower than that of voltage-controlled current sources, which typically exhibit impedance in the mega-ohm range." Two orders of magnitude lower is not slightly lower. It is substantially lower.

Version 1:

Reviewer comments:

Reviewer #1

(Remarks to the Author)
This manuscript has improved substantially following revision. The authors have carefully and thoroughly addressed the reviewers' comments, and the scientific significance of the work is now clearly articulated. I recommend acceptance of the manuscript.

Reviewer #2

(Remarks to the Author)
All my comments and suggestions were taken into consideration by the authors. I really appreciate all the changes made to the manuscript and the new experiments that have contributed to a real improvement in this paper.

Reviewer #3

(Remarks to the Author)
Thank authors for the impressively-detailed response and additional experiments addressing all previous comments and improving the manuscript. Overall, I find this revised manuscript is scientifically rigor and worthy publication in Nature Communications for broad interests.
I have still one confusion about the response to Reviewer 3 Comment 8, regarding modulated surface acoustic waves (SAWs) for AC current sources. I understand the new experiments shows no response when the SAW is modulated at 1 kHz. However it seems to be quite counterintuitive to me. The active length of the device is ~ 2 mm, the acoustic wave propagation speed is at a few km/s: thus when the input SAW is off, after microseconds (μs), all the SAW should have passed the active region, and thus the output current should be off. For careful consideration and avoid potential misleading to future readers, I have following comments:
(1) Does and load resistor used in the AC measurement? If the load is high resistance, it could be possible that the output resistor-capacitor (RC) limits the observation of AC current component. Especially in the Fig. 5 in Author Response, there is a large DC bias current, indicating there might be a large capacitor holding the output current/voltage.
(2) If the current response is so slow, maybe a current rise/fall time when turning on/off SAW could be measured?
(3) In Fig. 4c of previous paper [Hackett, L., Miller, M., Brimigion, F. et al. Towards single-chip radiofrequency signal processing via acoustoelectric electron–phonon interactions. Nat Commun 12, 2769 (2021). https://doi.org/10.1038/s41467-021-22935-1], the acousto-electric interaction shows μs level response time. Are there any significant difference in devices that lead to no AC response in this work? Do authors have any insights / comments explaining this no AC response?

Reviewer #5

(Remarks to the Author)
I co-reviewed this manuscript with one of the reviewers who provided the listed reports. This is part of the Nature Communications initiative to facilitate training in peer review and to provide appropriate recognition for Early Career Researchers who co-review manuscripts.

Reviewer #6

(Remarks to the Author)
The authors present a variable amplitude current source based on the acoustoelectric (AE) effect. The current source takes in an RF input power at the 430 MHz input frequency of a Rayleigh SAW IDT and outputs a DC current whose amplitude is proportional to the RF input power.

Unfortunately, the revisions have not alleviated my deep concerns with the significance of the technology demonstrated in this paper. Briefly, with the extremely poor properties of the current source demonstrated in this paper (e.g. efficiency of 0.0001% and output resistance of 10 kOhm) there are far more power and area efficient methods of constructing wireless current sources with much higher performance previously demonstrated in the literature. Since the authors have only responded to enumerated items from the previous review, I outline my detailed reasons for this assessment below.

1. This concept is not new and the reported work is not an advancement over previously published work. A superior performance device using LN (piezo) on Si (semiconductor) was reported by Mansoorzare in DOI: 10.1109/MEMS58180.2024.10439505 where an RF input power of 10 mW (at 661 MHz) produced a DC output current of 10 μA via the acoustoelectric effect compared to the approximately 2 μA current reported here for the same RF input power.

2. The current source still has an extremely poor RF-to-DC power conversion efficiency of 0.0001%. Despite claims to the contrary in the paper, this will result in significantly degraded performance when compared to traditional approaches of forming wireless current sources such as the use of rectifiers combined with transistor circuit based current sources. More on this below.

3. While acknowledging that transistor circuits can readily achieve nA level current sources, the authors still claim in the introduction "which makes stable, continuous operation at the nanoampere scale a significant challenge." Making nA level current sources with sustained operation is not at all challenging, especially ones with such poor conversion efficiency and output resistance.

4. The authors acknowledge that transistors can make nA scale current sources with much higher output resistance, but the claim is that these are not or cannot be wireless. To quote the response letter "In summary, while CMOS circuits can produce nanoampere currents, the acousticdriven current source offers unique advantages in terms of wireless operation, miniaturization, and energy harvesting, which are not achievable with standard transistor-based approaches." I could not disagree more. At the RF power levels in this paper, diode-based rectifiers have many orders of magnitude (~ 10^6) higher RF-to-DC conversion efficiency, meaning that a rectifier followed by a transistor current source easily realizes a much more efficient wireless current source and such current sources have been reported countless times in the literature. DOI 10.1186/s40486-017-0051-0 is a semi recent (2017) review article on RF power harvesting. Table 3 reviews rectifier technologies, where for example, http://dx.doi.org/10.1016/j.mejo.2015.11.012 achieves much higher RF-to-DC conversion efficiencies of greater than 50% across a wide range of load conditions. Again, there are many such rectifiers in the literature, some of which are listed in the review article. These rectifiers are small and implemented in transistor circuit technologies. A rectifier followed by a current source has been implemented on a single IC chip countless times to realize vastly superior wireless current sources.

5. In wirelessly powered applications, such as the one proposed by the authors, having a high output resistance is critical for the current source and it will need to be much higher than 10 kΩ. Let's explore why. The authors state in the paper, "Overall, while the output impedance of the acoustic driven current source is lower than that of conventional voltage-controlled current sources, it remains sufficiently high for intended low-power wireless applications, particularly when the load resistance is much smaller than the source impedance (e.g., < 1 kΩ), ensuring that current errors due to loading remain below 10%." Even at the highest RF input power levels input to the SAW in this paper of 200 mW (23 dBm), the current source only produces about 20 μA. If we source 20 μA into a 1 kΩ load as the authors suggest, the voltage supplied to the load will be 20 mV, far too low of a supply voltage to operate any known transistor or semiconductor logic technology. Perhaps the authors can cite what useful they could power at such a low voltage. Given the extremely poor RF-to-DC conversion efficiency of this device it can only power extremely low power circuits. Those circuits must have a supply volage of at least a few hundred mV which means, by definition, the load impedance must be large. Let's assume an ultralow supply voltage of 300 mV and the maximum current that can be supplied by this device of 20 μA at the ridiculously high received RF power level of 23 dBm for a wirelessly powered device. In such a case, the load impedance is 15 kΩ, above the output impedance of the current source, which means that the 300 mV won't even be achievable. If the RF input power were to drop to a still very large 10 mW (10 dBm), then the load impedance will need to increase to 150 kΩ, well above the output resistance of this current source. And the authors are claiming this current source can be used down to the nA level. I really can't see how this device can wirelessly power anything useful unless the conversion efficiency and output resistance are significantly improved.

Version 2:

Reviewer comments:

Reviewer #3

(Remarks to the Author)
In my opinion, the authors have successfully addressed all technical comments from both Reviewers by this revision. I recommend publication of this work in Nature Communications.

Reviewer #5

(Remarks to the Author)
I co-reviewed this manuscript with one of the reviewers who provided the listed reports. This is part of the Nature Communications initiative to facilitate training in peer review and to provide appropriate recognition for Early Career

Researchers who co-review manuscripts.

Reviewer #6

(Remarks to the Author)
The authors present a variable amplitude current source based on the acoustoelectric (AE) effect. The current source takes in an RF input power at the 430 MHz input frequency of a Rayleigh SAW IDT and outputs a DC current whose amplitude is proportional to the RF input power.

I thank the authors for more directly addressing my review of their paper, and specifically the limitations of the reported technology, in this round of revision. I have grave reservations about the impact of this paper and its appropriateness for publication in Nature Communications. If it is to be published in such a widely read journal that targets very high impact scientific findings to a broad audience, in my view the context provided by the authors needs a presentation where a non-expert can clearly see the significant limitations of the work. My comments below should not be interpreted as an endorsement to publish this paper in Nature Communications, it should be taken as feedback to further improve the paper should the editors ultimately decide to publish it.

1. It is well understood in energy harvesting that highly efficient RF-to-DC conversion is achievable at the large input powers reported in this paper. It is also quite common to show a plot of Pout (DC) on the y-axis vs. Pin (RF) on the x-axis. I understand the authors are presenting a current source, but ultimately the current source is to deliver wireless power, and it does so with a very poor efficiency (0.0001% and output resistance of 10 kOhm) whose value is not explicitly reported in the paper. The authors should include a plot of Pout(DC) vs. Pin(RF) of their current source (simply match it for maximum power transfer) and on the same graph include points or curves from state-of-the art literature. The Nature Comms readers should be able to clearly see this technology in its current form is 6 orders of magnitude lower in conversion efficiency when compared to the state-of-the-art and will require significant advancements before it exhibits practical utility. I see no path where increases in electromechanical coupling (at most 10x for known piezo materials and modes) and arraying close a gap of 6 orders of magnitude in performance.

2. In response to my prior feedback the authors point to the fact that traditional WPT systems include a cascade of stages: “Wireless power transfer (WPT) offers a viable pathway toward autonomous and untethered operation. In the conventional WPT systems, a stable DC output is typically obtained through a cascade of functional stages, including an antenna for RF reception, impedance matching networks, nonlinear rectification elements, and filtering components. While effective, such multi-stage architectures inevitably increase system-level complexity and introduce inherent trade-offs among device size, transmission distance, and output stability, particularly under high-loss or harsh operating environments.” (Page 3). The authors should acknowledge that at the input powers where their technology produces useful output currents of more than 1nA, that the traditional WPT systems become significantly simplified. No impedance matching circuits will be required at these input powers since the voltages are well in excess of the diode turn on voltage. As such, rectifiers can be extremely small (simply 4 tiny diodes on a chip) and realized in extremely inexpensive Si technology. The only possible advantage I can see is the removal of the smoothing capacitor, as both traditional and acousto-electric WPT will require an antenna.

3. The authors provide a biomedical interface application where their current source would have functional utility, and generally I agree the DC currents produced, and the frequency response demonstrated would be applicable to biological stimulation. However, the poor efficiency is also quite limiting in this application. The IEEE Standard for Safety Levels with Respect to Human Exposure to Radio Frequency Electromagnetic Fields, 3kHz to 300GHz”, IEEE C95.1-1991 limits the maximum continuous power density on the body at 430 MHz (the frequency of the reported device) to approximately 0.3 mW/cm2. According to the authors’ response letter, “In clinical microcurrent therapy and studies promoting cellular galvanotaxis, the recognized effective therapeutic window for physiological modulation is typically in the range of microamperes (e.g., 10 μA to 20 μA), without the need for high driving voltages.” To produce just 1 μA of stimulation current, the reported device requires 10 mW of received power. This will require at a minimum a 33 cm2 receive antenna to remain within the safe exposure limits and this analysis doesn’t even account for any loss between the transmit and receive antennas in the proposed biomedical application. If 10 μA of current is needed as identified by the authors, 100 mW of received power will be needed requiring a 333 cm2 receive antenna (18 x 18 cm!), again with no loss between the transmit and receive antennas which will make this situation much worse. A simple inductive loop, diode, and smoothing capacitor could do this over a 1 cm distance while occupying less than 1 mm on a side. Go to 1 cm on a side, and 5 cm distances with 100 mW level power delivery is commonly reported, from which any biologically relevant current can be delivered. The proposed biomedical application simply highlights the premium placed on efficiency in WPT applications and why no device that is 1 million times less efficient than the state-of-the-art can claim to simply the system. I go back to another comment from the authors about traditional WPT “While effective, such multi-stage architectures inevitably increase system-level complexity and introduce inherent trade-offs among device size, transmission distance, and output stability, particularly under high-loss or harsh operating environments.” The poor efficiency of the reported device greatly exasperates the tradeoffs between size and transmission distance. It cannot be reasonably claimed via a quantitative analysis that it will simplify these tradeoffs.

Open Access This Peer Review File is licensed under a Creative Commons Attribution 4.0 International License, which permits use, sharing, adaptation, distribution and reproduction in any medium or format, as long as you give appropriate credit to the original author(s) and the source, provide a link to the Creative Commons license, and indicate if changes were made.
In cases where reviewers are anonymous, credit should be given to 'Anonymous Referee' and the source.
The images or other third party material in this Peer Review File are included in the article's Creative Commons license, unless indicated otherwise in a credit line to the material. If material is not included in the article's Creative Commons license and your intended use is not permitted by statutory regulation or exceeds the permitted use, you will need to obtain permission directly from the copyright holder.
To view a copy of this license, visit https://creativecommons.org/licenses/by/4.0/

Reviewer: 1

Comment

1. On page 5, the authors mentioned that 'There is a lattice mismatch between $LiNbO_3$ and InSb, and oxygen diffusion from $LiNbO_3$ into InSb leads to a degradation in the performance. Therefore, an $In_{0.6}Al_{0.4}Sb$ buffer layer was inserted between InSb and the substrate". However, the manuscript lacks a thorough discussion on the role of the buffer layer. Why the buffer layer can solve the problem of lattice mismatch and oxygen diffusion? What are the electrical property differences between the devices with and without the buffer layer?

Response:

We sincerely appreciate the reviewer's valuable comments. In response to your suggestions, we have systematically investigated and discussed the specific roles of the $In_{0.6}Al_{0.4}Sb$ buffer layer in mitigating lattice mismatch and suppressing oxygen diffusion in the revised manuscript (see Supplementary Note I). Our responses are structured in the following three parts:

1. Mechanism of Lattice Mismatch Mitigation by the Buffer Layer

As predicted by the Vegard's Law[1], the selected buffer layer, $In_{0.6}Al_{0.4}Sb$, has a lattice constant between those of AlSb (6.135 Å) and InSb (6.479 Å). However, there exists a significant lattice mismatch between $LiNbO_3$ and InSb, which would lead to a high density of crystal defects if the InSb is directly deposited. The lattice parameter of $In_{0.6}Al_{0.4}Sb$ is quite compatible with that of InSb, facilitating the epitaxial growth of high-quality InSb films on top of the buffer layer and significantly reducing dislocations and structural defects.

Besides, we also verified the structural improvement experimentally by conducting grazing incidence X-ray diffraction (GIXRD) on the prepared samples with and without using the buffer layer. The introduction of $In_{0.6}Al_{0.4}Sb$ leads to much stronger diffraction peak intensities and narrower full width at half maximum (FWHM) values for InSb, confirming the enhanced crystalline quality (see Fig. 1).

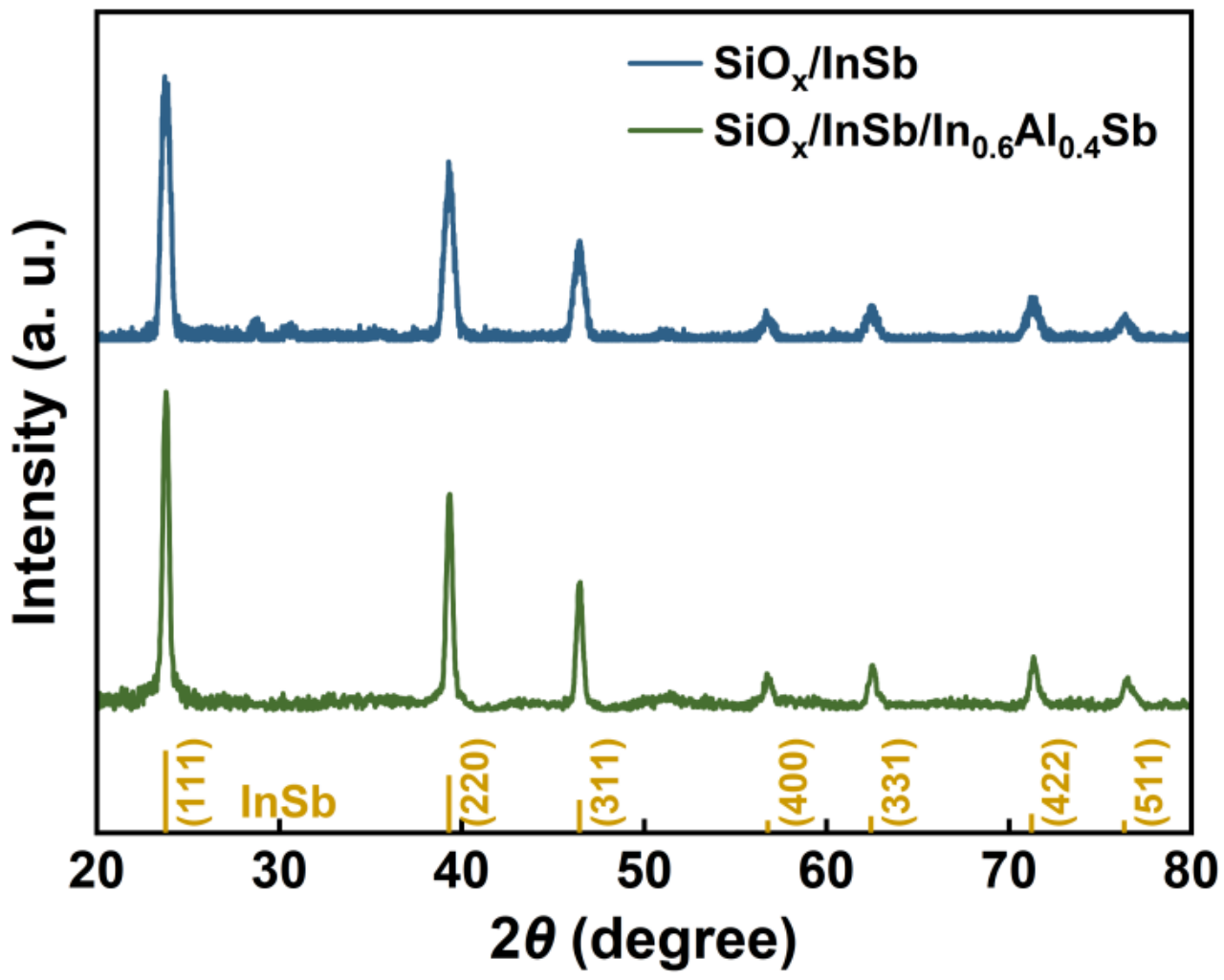

Fig. 1 GIXRD pattern of the InSb layer in SiO_x/InSb and SiO_x/InSb/$In_{0.6}Al_{0.4}Sb$ films.

2. Mechanism of Oxygen Diffusion Suppression by Using the Buffer Layer

Oxygen diffusion from $LiNbO_3$ into the overlying InSb layer can seriously deteriorate its electrical performance[2].

From the structural aspect, although the earlier studies have used dielectric layers such as SiO_x for partial isolation[3], their loose structure, poor thermal stability, and lack of effective oxygen-trapping mechanisms limit their blocking effectiveness. In contrast, $In_{0.6}Al_{0.4}Sb$, being as a III-V compound semiconductor material, possesses high crystallinity and thermal stability, mainly attributed to its continuous barrier interface.

From the chemical aspect, the presence of Al atoms in the $In_{0.6}Al_{0.4}Sb$ enables the formation of strong Al-O bonds, providing its superior ability to suppress oxygen migration from $LiNbO_3$. This facilitates the chemical trapping of diffusing oxygen species, further enhancing the blocking efficiency of such a buffer layer.

3. Impact on Electrical Properties

We fabricated two sets of multilayer structures on $LiNbO_3$ substrates: SiO_x/InSb and SiO_x/InSb/$In_{0.6}Al_{0.4}Sb$, and performed the Hall effect measurements. The measurement results showed that the sample with the buffer layer exhibited an increased mobility of 1700 $cm^2/V\cdot s$ (compared to 520 $cm^2/V\cdot s$ without the buffer), along with more stable carrier concentrations (about 7×10^{11} cm^{-2}). These results indicate the improved crystal quality and interface properties (see Fig. 2).

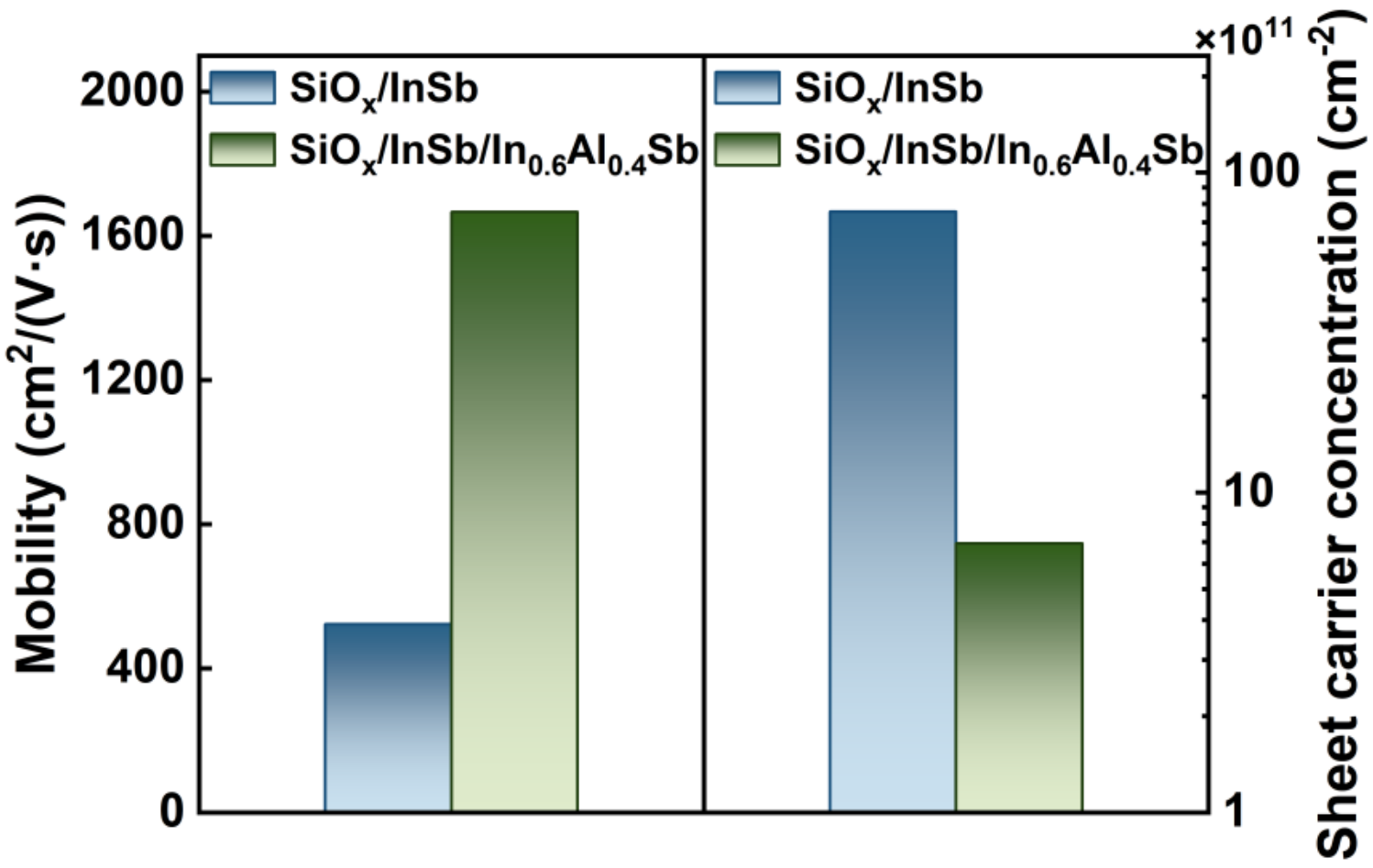

Fig. 2 Carrier mobility and sheet carrier concentration of SiO_x/InSb and SiO_x/InSb/$In_{0.6}Al_{0.4}Sb$ films.

These revisions, including additional experimental data and the added relevant literature, have been incorporated into the **Results section ("Material platform overview", Page 4)**. Once again, we thank you for pointing out the needs for more detailed explanations in this section.

References:

1. Schwalbe, C., H. Lars Vegard: key communicator and pioneer crystallographer. *Crystallography Reviews* **20**, 9–24 (2013).

2. Coldren, L., A. Trapping model for InSb thin films. *Applied Physics Letters* **18**, 319–321 (1971).

3. Yamanouchi, K. & Shibayama, K. Monolithic elastic surface wave amplifiers of continuous operation. *Japanese Journal of Applied Physics* **16**, 493–496 (1977).

Revised:

1. "**Fig. 2 Structural and electrical characterization of SiO_x/InSb/$In_{0.6}Al_{0.4}Sb$ heterostructure films. a** GIXRD patterns of the InSb layer, and **b** mobility and sheet carrier concentration of SiO_x/InSb and SiO_x/InSb/$In_{0.6}Al_{0.4}Sb$ films." (Page 6)

Comment

2. What is the thickness of the buffer layer? How were the thickness values for both the buffer layer and the 250 nm InSb film determined? How was the atomic percentage of In and Al selected? Would variations in layer thickness or atomic composition affect

the electrical properties of the device? A discussion or experimental validation is suggested to be added.

Response:

We sincerely thank the reviewer for raising these insightful questions. In response, we have added detailed discussions and experimental validations in the revised manuscript. Our responses are summarized as follows:

1. Thickness of the Buffer and Active Layers, and Measurement Methods

In this study, the $In_{0.6}Al_{0.4}Sb$ buffer layer was designed with a thickness of 150 nm, and the active InSb layer was 250 nm thick. Both thicknesses were confirmed by using the cross-sectional scanning electron microscope (SEM). The selection of these values is mainly based on the following considerations (Yamanouchi, K. et al. EP0810726, 2005):

(1) For the **buffer layer**, a moderate thickness is essential to reduce the defect density and enhance the crystalline quality of the overgrown InSb film. However, an overly thick buffer could short-circuit the accompanying electric field of surface acoustic waves (SAWs), limiting its ability to penetrate and interact with carriers in the active layer. A thickness of 150 nm was selected to balance good crystalline quality with acoustic-electric interaction efficiency.

(2) For the **InSb active layer**, a too-thin film results in poor crystallinity and low mobility, while an excessively thick layer leads to increased surface conductivity, thereby reducing the SAW-electron interaction. By controlling the sputtering time, a thickness of 250 nm for the InSb layer was selected to ensure both high carrier mobility and effective acoustic-electric coupling.

2. Atomic Composition of the Buffer Layer and Its Impact on Electrical Properties

The $In_yAl_{1-y}Sb$ buffer layer was fabricated using a co-sputtering process involving three targets of InSb, Al, and Sb, with specific deposition parameters provided in Table 1.

Table 1 Process parameters of the buffer layer in the study on the atomic composition of $In_yAl_{1-y}Sb$

InSb sputtering power (W)	Al sputtering power (W)	Sb sputtering power (W)	Sputtering pressure (Pa)	Deposition temperature (°C)	Sputtering time (min)	In-situ annealing time (min)
60/80/100/120/140	43	30	0.5	450	10	60

By adjusting the sputtering power of the InSb target, we systematically investigated the influence of In/Al atomic ratios on the electrical properties of the multilayer structure (see Fig. 3). The elemental ratios were determined via energy-dispersive X-ray spectroscopy (EDS), and the results are shown in Table 2:

(1) **Carrier mobility** initially increases and then decreases with increasing In/Al ratio, peaking at a value of 630 $cm^2/(V \cdot s)$ when In/Al ≈ 0.56/0.44.

(2) **Sheet carrier concentration** exhibits an inverse trend with the above, reaching a minimum of 1.6×10^{12} cm^{-2} at the same composition.

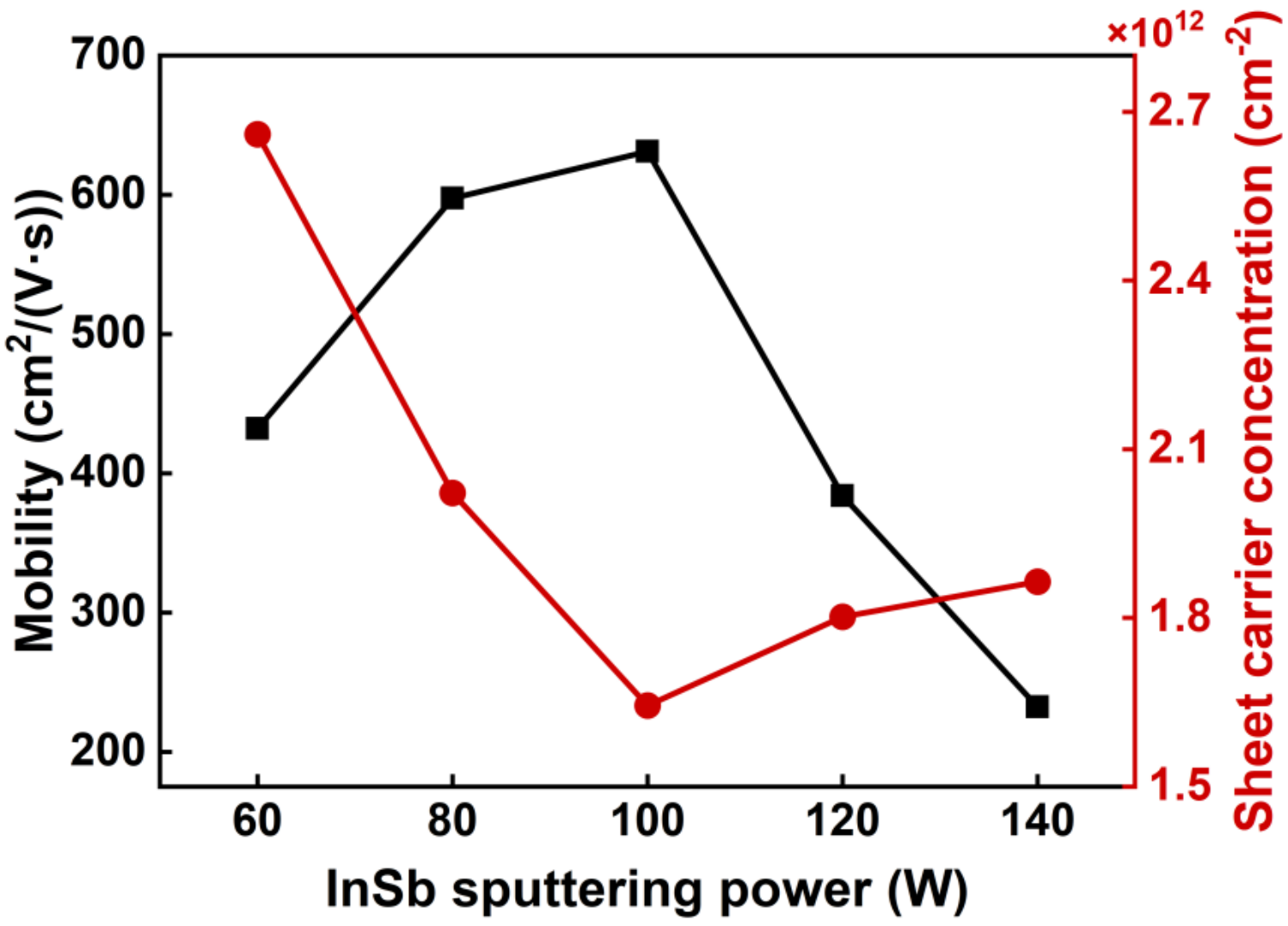

Fig. 3 Carrier mobility and sheet carrier concentration of $SiO_x/InSb/In_yAl_{1-y}Sb$ films as a function of InSb sputtering power (60-140 W).

Table 2 Atomic percentages of each element in $In_yAl_{1-y}Sb$ films fabricated at different InSb sputtering powers

InSb sputtering power (W)	In (at%)	Al (at%)	Sb (at%)	In/Al	$In_yAl_{1-y}Sb$
60	18.29	32.00	49.71	0.57	$In_{0.36}Al_{0.64}Sb$
80	24.56	25.45	49.99	0.97	$In_{0.49}Al_{0.51}Sb$
100	27.98	22.26	49.76	1.26	$In_{0.56}Al_{0.44}Sb$
120	30.60	19.60	49.81	1.56	$In_{0.61}Al_{0.39}Sb$
140	32.64	17.14	50.22	1.90	$In_{0.66}Al_{0.34}Sb$

These trends are attributed to the interplays among lattice matching, crystalline quality, and surface roughness. It should be noted that this experiment is a preliminary study of this work, and some process parameters (such as the sputtering power and deposition

temperature of the active layer) have not been optimized. Therefore, the carrier mobility and sheet carrier concentration at this stage exhibit certain differences from the optimized test data described in the manuscript.

In summary, we have added detailed discussions and experimental results on the effects of the thicknesses of the buffer and active layers, as well as the atomic composition of the buffer layer, on the electrical properties of the multilayer structure. These revisions are intended to provide a comprehensive understanding of the design rationale and performance optimization strategies. We greatly appreciate the reviewer's constructive suggestions, which have helped us significantly improve the quality and completeness of the manuscript.

Comment

3. On page 11, in Fig 4a, the authors claimed "the current accuracy was remained below 3%, exhibiting an increasing trend with the rise of I_{set}". However, the data in Figure 4a shows that I_{acc} achieved its lowest value at I_{set}=100 nA. Please clarify and provide an explanation for this phenomenon.

Response:

We sincerely appreciate the reviewer's valuable feedback regarding to the presentation inaccuracies in Fig. 4a. We intended to show that the current accuracy demonstrates an improving trend (i.e., decreasing error) with increasing I_{set}. We apologize for any misunderstanding caused by our wording in the previous version. We confirm that all data represent raw experimental results without selective presentation. The corrections have been made in the revised manuscript, as highlighted in red.

Revised:

2. "As shown in Fig. 4a, the current accuracy was maintained below 3% and exhibited the improvement with increasing I_{set}, reaching a local minimum at I_{set}=100 nA." (Page 11)

Comment

4. The manuscript repeatedly highlights the need for miniaturized current sources. However, the exact size of the reported device is not explicitly stated. Please provide

quantitative size specifications of this current source.

Response:

We appreciate the reviewer's valuable comment. The structural dimensions of the acoustic-driven current source have been added to the revised manuscript. The active region, comprising the IDTs and the multilayer film, occupies a footprint of approximately 2.3 mm × 0.5 mm × 0.5 mm (length × width × height), demonstrating the device's miniaturized form factor. An optical micrograph of the fabricated acoustic-driven current source is shown in Fig. .

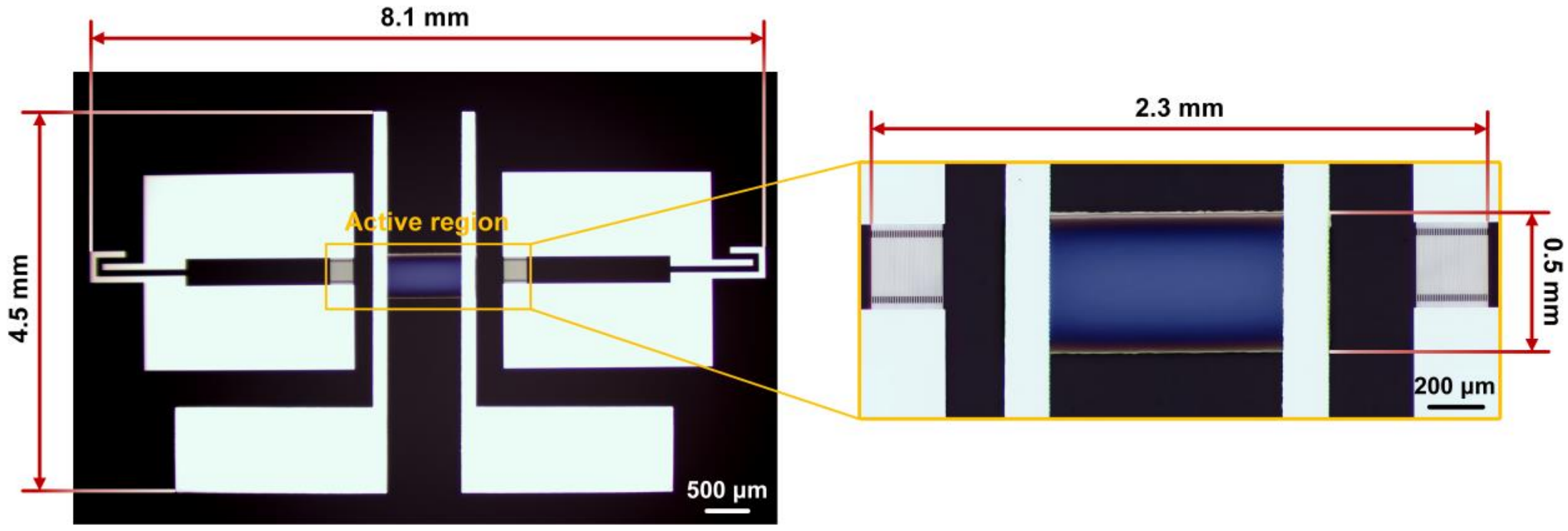

Fig. 4 Optical micrograph of the fabricated acoustic-driven current source.

Revised:

1. "The active region of the current source, which includes the IDTs and the multilayer film, has a compact dimension of approximately 2.3 mm × 0.5 mm × 0.5 mm (length × width × height), reflecting the device's miniaturized design." (Page 21)

Comment

5. The authors suggest that this device could be useful for sensor networks and implantable medical devices. However, there is no detailed discussion on specific application scenarios that require a current source with an output as low as the nanoampere range. Furthermore, considering that the reported wireless transmission distance is less than 60 cm, is this sufficient for practical use in implantable devices? An experimental demonstration is suggested to be added.

Response:

We sincerely thank the reviewer for the insightful suggestion. In response, we have expanded the revised manuscript to include a more detailed discussion of potential

application scenarios for the proposed nanoampere-level current source and have also conducted additional experiments to demonstrate its feasibility in practical biomedical use.

To illustrate this point, there is a growing demand for miniaturized and low-power current sources in several emerging fields, particularly in the following representative scenarios:

1. Implantable Medical Devices

Microcurrent stimulators have been applied in neural modulation, tissue regeneration, and pain relief therapy[1-3]. Previous studies have shown that constant or pulsed microcurrents in the nanoampere to microampere range can effectively promote cell repair and tissue growth. These systems are typically implanted in shallow subcutaneous layers, where conventional power supplies face challenges such as bulky wiring and heat generation. Therefore, battery-free and RF-activated current sources are highly desirable for such biomedical applications.

2. Wireless Sensor Network Nodes

Many low-power sensors—for example, those used in gas sensing, biosensing, or structural strain monitoring—operate under average power levels ranging from nanowatts to microwatts. These systems can benefit from a compact and remotely activated DC bias current source capable of maintaining basic sensing functionality or enabling periodic wake-up[4]. The proposed acoustoelectric current source, with its high integration and wireless activation, offers distinct advantages for such applications.

3. Acoustic RF Signal Processing Systems

Furthermore, with the recent advancement of fully acoustic RF signal processing systems, a new class of platforms based on piezoelectric and SAW devices for filtering, amplification, and non-reciprocity has emerged[5-7]. These systems use acoustic waves as the sole information carriers and demonstrate strong resilience to electromagnetic interference, making them suitable for extreme environments. A critical bottleneck, however, lies in the absence of acoustically compatible microscale power sources. The proposed acoustoelectric current source could serve as an ideal companion module by providing a stable DC output under acoustic excitation, thereby enabling a truly low-power, all-acoustic, single-chip RF signal processing platform.

In response to the reviewer's question on whether the demonstrated wireless transmission distance (<60 cm) is sufficient for implantable applications, it should be noted that most existing implantable systems (e.g., neural stimulators and wireless therapeutic chips) are typically positioned at depths of 5–20 mm beneath the skin and are powered via near-field coupling[8]. Thus, the demonstrated transmission range is well within the practical requirement for such subcutaneous applications.

To further demonstrate the potential biomedical applicability, an in vivo wound-healing experiment was conducted using the acoustic-driven current source. The results confirmed that low-intensity (10 μA) current stimulation effectively accelerated wound closure and improved healing stability, demonstrating the feasibility of employing the proposed current source for bioelectronic therapy. These findings represent an important step toward translating the acoustic-driven current source into practical biomedical applications.

These revisions have been incorporated into the revised manuscript, specifically in the **Results section ("Wireless acoustic-driven current source", Page 13)**. Relevant references have also been added to strengthen the discussion and highlight the novelty and practical significance of our work.

References:

1. Famm, K. *et al*. A jump-start for electroceuticals. *Nature* **496**, 159-161 (2013).
2. Lee, J. *et al*. Neural recording and stimulation using wireless networks of microimplants. *Nature Electronics* **4**, 604-614 (2021).
3. Huang, Y. *et al*. Bioelectronics for electrical stimulation: materials, devices and biomedical applications. *Chemical Society Reviews* **53**, 8632-8712 (2024).
4. Zahid Kausar, A. S. M., Reza, A. W., Saleh, M. U. & Ramiah, H. Energizing wireless sensor networks by energy harvesting systems: Scopes, challenges and approaches. *Renewable and Sustainable Energy Reviews* **38**, 973-989 (2014).
5. Hackett, L. *et al*. Giant electron-mediated phononic nonlinearity in semiconductor–piezoelectric heterostructures. *Nature Materials* **23**, 1386-1393 (2024).
6. Hackett, L. *et al*. Non-reciprocal acoustoelectric microwave amplifiers with net gain and low noise in continuous operation. *Nature Electronics* **6**, 76-85 (2023).
7. Hackett, L. *et al*. Towards single-chip radiofrequency signal processing via acoustoelectric electron-phonon interactions. *Nature Communications* **12**, 2769 (2021).
8. Singer, A. & Robinson, J. T. Wireless power delivery techniques for miniature

implantable bioelectronics. *Advanced Healthcare Materials* **10**, 2100664 (2021).

Comment

6. The manuscript contains several errors that should be carefully checked. Examples include: on page 8 line 158, "In Figure 3b" should be "In Figure 2b"; on page 11 line 236, sampling rate missed the symbol.

Response:

We thank the reviewer for identifying these issues. Comprehensive revisions have been made throughout the manuscript to ensure the writing, grammar and precise wording. The corrections have been made in the revised manuscript, as highlighted in red.

Revised:

1. "In Figure 3a, a frequency sweeping signal (400-460 MHz) with various input powers (0 dBm, 5 dBm, 10 dBm) was applied to the IDT. The AE current, which is a DC current output generated from the input RF signal via the AE effect, was then measured without any applied DC bias." (Page 8)

2. "The electrical characteristics of the current, with input RF powers of 0.01, 0.1, 1, 10, and 100 mW, were analyzed over a sampling time of T=50 s and a sampling rate F_s=12.5 Hz." (Page 11)

Reviewer: 2

Comment

1. Page 6, Line 135: It is stated that The AE current is directly proportional to the K_{eff}^2 and inversely proportional to the square of v_S. However, in formula (1), the frequency (f=v_S/lamda) appears in the numerator. It is therefore more correct to say that AE current is inversely proportional to v_S and not to the square of v_S. It is more intuitive to talk about the wavelength (Lamda) since the greater Lamda is, the more the wave penetrates the substrate and less the surface, resulting in a weaker current.

Response:

We sincerely appreciate the reviewer's careful reading and insightful comment regarding the relationship between AE current and acoustic velocity v_S. The reviewer is absolutely correct in pointing out the inconsistency. We have modified thorough corrections in the revised manuscript, as highlighted in red.

Revised:

1. "where μ is the mobility of the AE film, P represents the power of SAW at the center of the film, calculated from the input RF power and the transmission coefficient S_{21} of the device. f=v_S/λ is the frequency of the SAW, with v_S and λ denoting the acoustic velocity and wavelength, respectively." (Page 5)
2. "(1) The piezoelectric substrate. The AE current is directly proportional to the K_{eff}^2 and inversely proportional to the v_S. (2) The IDT structure. The AE current reaches its maximum value at the resonant frequency defined by the IDT period (i.e., wavelength λ), where the acoustic energy transfer to carriers is maximized. Notably, longer wavelengths enhance bulk wave penetration at the expense of surface energy density, thereby reducing the AE current amplitude." (Page 5)

Comment

2. Figure 2 (b): It is not clear whether this is a simulation or an experimental measurement.

Response:

We confirm that Figure 2(b) presents the experimental measurement results, not the simulation results. We have made thorough corrections in the revised manuscript, as highlighted in red.

Revised:

1. "**a** Measured AE current (left axis) and S_{21} (right axis) versus frequency at different input RF powers." (Page 8)
2. "In Figure 3a, a frequency sweeping signal (400-460 MHz) with various input powers (0 dBm, 5 dBm, 10 dBm) was applied to the IDT. The AE current, which is a DC current output generated from the input RF signal via the AE effect, was then measured without any applied DC bias. The contour of the AE currents closely resembled the measured transmission coefficient S_{21}, demonstrating a significant correlation between them (see Supplementary Note 3 for frequency response characteristics of the device)." (Page 8)

Comment

3. Please explain how the losses in the InSb layer are modelled and what physical parameters are used to carry out the simulation.

Response:

We thank the reviewer for this valuable comment. A coupling of modes (COM) model was established to simulate the SAW transmission. The model consists of three basic units—input IDT, acoustic propagation path, and output IDT—each represented by a *P*-matrix, and the frequency response characteristics of the SAW device were obtained by cascading these matrices. To account for the losses in the InSb layer, a complex propagation parameter, $\gamma=\alpha+j\beta$, was introduced, where α and β describe the SAW attenuation and velocity change, respectively. The key physical parameters used in the simulation include the carrier mobility, carrier concentration, and sheet conductivity of the InSb-based multilayer film, as well as the electromechanical coupling coefficient, acoustic velocity, and effective dielectric constant of the 128°YX $LiNbO_3$ substrate. For the acoustic propagation path covered by the InSb-based multilayer film, γ was incorporated into the corresponding *P*-matrix to model the AE effect. A more detailed description of this modelling approach and parameter selection has been provided in our previous work[1].

References:

1. Yuan, Y. *et al.* Analysis of the acoustoelectric response of SAW gas sensors using a COM model. *Microsystems & Nanoengineering* **10**, 69 (2024).

Comment

4. Figure 5 (a): For the wired configuration, we understand the advantage of using a double port SAW device (delay line) to allow S_{21} characterization. However, in a wireless configuration this advantage disappears and a single IDT connected to the antenna is sufficient. It may be appropriate to connect both IDTs to the antenna to generate a standing wave, in which case the position of the electrodes on the InSb layer needs to be modified.

Response:

We sincerely thank the reviewer for this valuable suggestion, which provides important guidance for improving the device performance. As recommended, in the wireless configuration of the acoustic-driven current source, connecting a single IDT to the antenna is sufficient to meet the application requirements. To verify this, we have conducted additional comparative experiments as suggested, which further confirm the feasibility and effectiveness of the single IDT solution.

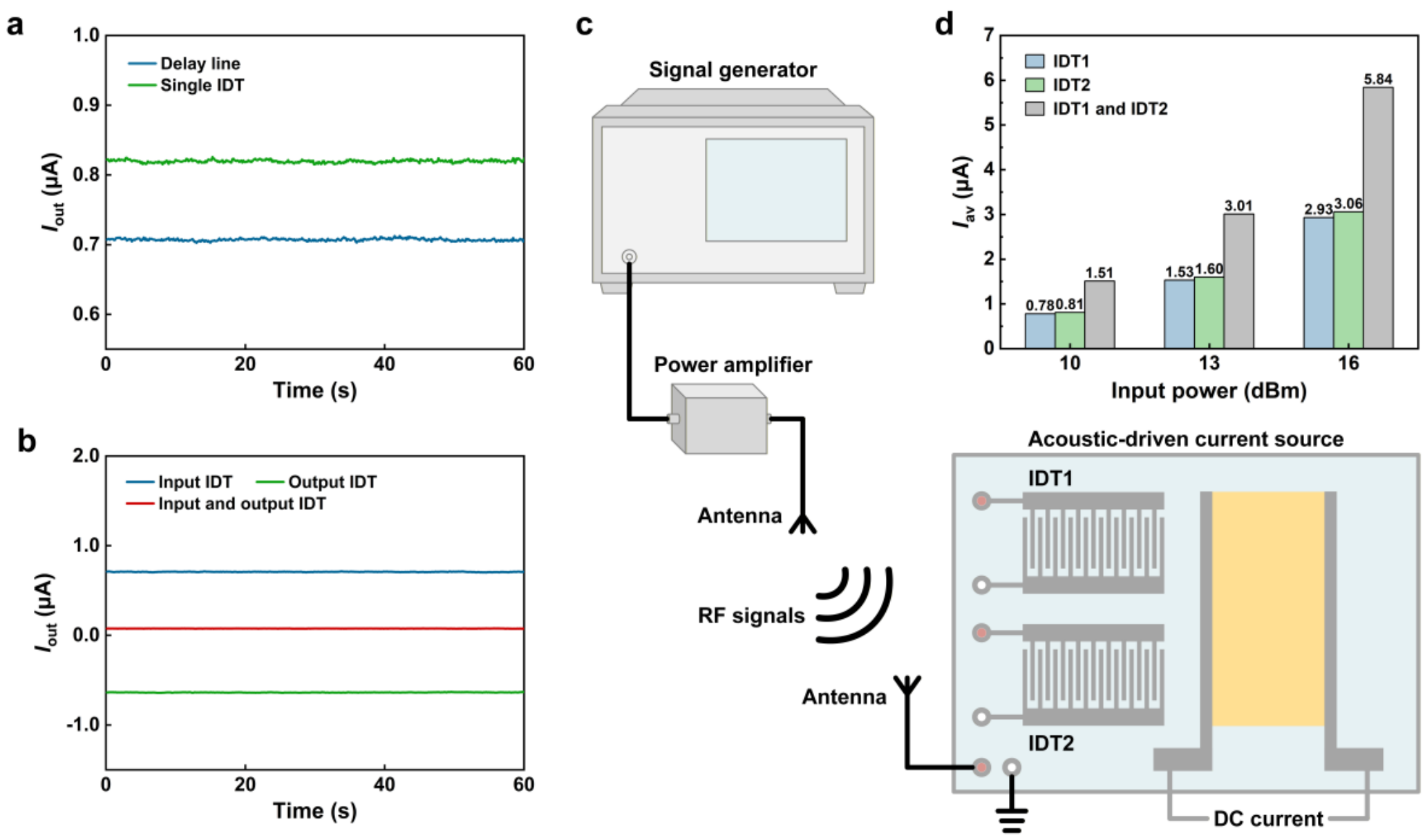

Fig. 5 At a RF power of 10 dBm and a wireless transmission distance of 10 cm, **a** comparison of I_{out} between the delay-line-based and single-IDT-based structures, and **b** comparison of I_{out} when the input and output IDTs in the delay-line-based structure are connected to signals separately and simultaneously. **c** Schematic diagram of the test system for the acoustic-driven current source array. **d**

I_{out} of the acoustic-driven current source array at different RF powers with a wireless transmission distance of 10 cm.

As shown in Fig. a, with a RF power of 10 dBm and a wireless transmission distance of 10 cm, the comparison between the acoustic delay line and the single input IDT structure (with the output IDT disabled) reveals the following: the acoustic-driven current source based on the delay line exhibits an average output current I_{av} of 0.71 μA, whereas the single input IDT scheme achieves an I_{av} of 0.82 μA, representing an approximately 15.5% improvement over the former Fig. b further illustrates that, for the acoustic-driven current source based on the delay line: when only the input IDT is connected to the signal, I_{av} = 0.71 μA; when only the output IDT is connected, I_{av} = -0.64 μA; and when both the input and output IDTs are connected, I_{av} = 0.08 μA, which approximates the algebraic sum of the output currents in the first two scenarios.

To investigate the scalability and stability of the single IDT architecture, we constructed an acoustic-driven current source array composed of multiple IDTs and acoustoelectric film structures. As shown in Fig. c, two IDTs (IDT1 and IDT2) fully consistent with the previously detailed structural parameters in the revised manuscript are arranged in parallel, with two antenna configurations of identical type employed for wireless excitation. The blue squares in Fig. d denote I_{av} of IDT1, the green squares denote I_{av} of IDT2, and the gray squares denote the total I_{av} when the two are connected in parallel. Under three RF power levels (10 dBm, 13 dBm, and 16 dBm) and a wireless transmission distance of 10 cm, I_{av} values of IDT1 in individual operation are 0.78 μA, 1.53 μA, and 2.93 μA, respectively; those of IDT2 in individual operation are 0.81 μA, 1.60 μA, and 3.06 μA, respectively; and the total I_{av} values when both IDTs operate simultaneously are 1.51 μA, 3.01 μA, and 5.84 μA, respectively. The output difference between IDT1 and IDT2 primarily stems from fabrication errors and differences in signal transmission path losses, while the total acoustoelectric current induced by the two rows of traveling wave excitation approximates the linear sum of the output currents of the two IDTs in individual operation. Experimental results confirm that the single IDT architecture is feasible and demonstrates current summation characteristics in a wirelessly extended configuration.

From a physical mechanism standpoint: In a complete delay line, the interdigitated electrodes of the output IDT act as strong reflective interfaces for surface acoustic waves. When acoustic waves excited by the input IDT propagate to the output IDT, they are reflected by its interdigitated electrodes, generating reverse acoustic waves. These reverse waves induce reverse currents in the acoustoelectric film; when superimposed

with forward currents, these reverse currents partially cancel the output. Disabling the output IDT eliminates the reflective interface, significantly weakens reverse acoustic waves and their associated reverse currents, and thereby enhances the total output current. Experimental results are consistent with theoretical predictions.

In the acoustoelectric film, forward waves excited by the input IDT and reverse waves excited by the output IDT induce opposite carrier drift directions, resulting in opposite current polarities. The difference in the absolute value of the converted currents when the two waves act individually primarily arises from imperfect symmetry due to fabrication errors (e.g., IDT structural deviations, thin-film defects, among others). When both the input and output IDTs are driven by signals, surface acoustic waves excited by each IDT exhibit stable interference within the acoustoelectric film.

Ideally, in a device with perfectly symmetric structures, the two traveling waves exhibit identical frequencies, equal amplitudes, and opposite propagation directions. Upon superposition, they form a standing wave, wherein acoustic energy oscillates solely between antinodes and nodes without directional propagation—and thus fail to generate a macroscopically directed acoustoelectric current—such that the total output current is theoretically zero. However, in practice, achieving perfect structural symmetry in devices is challenging, leading to subtle differences in the propagation characteristics of the two traveling waves. After superposition, the standing wave is impure, retaining partial traveling-wave components. These traveling-wave components can drive charge carriers to form a directed current, resulting in a total output current approximately equal to the algebraic sum of the currents generated by the input and output IDTs operating independently.

This result validates the linearity of acoustoelectric conversion in this acoustic-driven current source: the total current when multiple IDTs operate can be achieved by superimposing the currents from each IDT operating individually.

We thank the reviewer for pointing out the need for more detailed explanations in this section. In response, we have supplemented additional experimental data and included the relevant literature to strengthen the discussion. These revisions have been incorporated into the revised manuscript, specifically in the **Results section (“Different Excitation IDT Schemes”, Page 15)** and in the **Methods section (“Device fabrication”, Page 20)**.

Comment

5. It should also be pointed out that half of the RF energy converted into an elastic surface wave is lost due to the propagation of the wave in both directions. A unidirectional IDT will be relevant.

Response:

We appreciate the reviewer's identification of a critical issue: following the conversion of RF energy into surface acoustic waves, half of the energy is lost due to their bidirectional propagation. To address this problem, we have incorporated a single-phase unidirectional transducer (SPUDT) to enable unidirectional focusing of acoustic wave energy, thereby improving energy transfer efficiency. Furthermore, we have supplemented the corresponding experimental validation results.

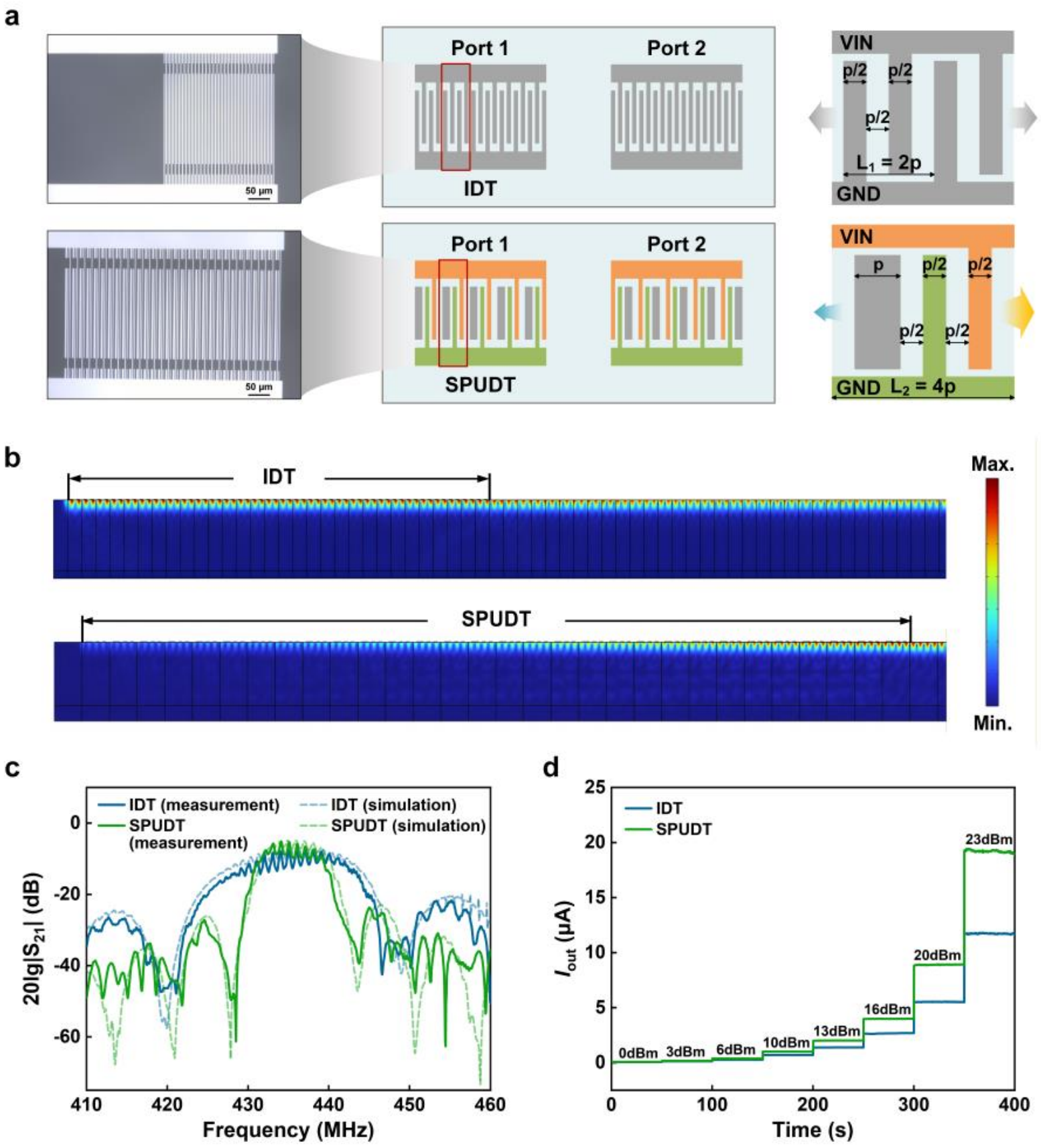

Fig. 6 **A** Schematic diagrams and optical micrographs of IDT and SPUDT delay lines. **b** Displacement field simulations of the IDT and SPUDT. **c** Measured and simulated S_{21} parameters of the IDT and SPUDT delay lines: blue and green lines correspond to the S_{21} curves of the two delay lines, respectively, with solid lines representing measured data and dashed lines representing simulated data. **d** Power-current responses of the two types of delay line-based acoustic-driven current sources (power range: 0-23 dBm; wireless transmission distance: 10 cm).

Specifically, we designed an acoustic-driven current source featuring a SPUDT delay line structure. The core differences between the SPUDT and IDT delay lines are illustrated in Fig. a:

1. In the IDT delay line, the IDT unit period is $L_1 = 2p = 8.92$ μm, and each period contains two electrodes, which are connected to the signal terminal and ground terminal, respectively;

2. In the SPUDT, the unit period is extended to $L_2 = 4p = 17.84$ μm, with each period containing three electrodes: two electrodes (each with a width of $p/2$) connected to the signal terminal and ground terminal, respectively, and one floating electrode (with a width of p) for acoustic wave reflection. In the SPUDT unit period shown in the lower right of Fig. a, right-propagating acoustic waves are enhanced, while left-propagating ones are significantly attenuated, thereby achieving unidirectional energy transmission. The SPUDT delay line comprises two symmetrically distributed SPUDTs.

The number of periods (30), aperture width (223 μm), electrode thickness (275 nm), and delay distance (1784 μm) are kept consistent for both structures to ensure the validity of the comparison. Finite element method (FEM) simulation results (Fig. b) clearly show the acoustic wave displacement distribution in the input IDT and SPUDT regions, directly verifying the unidirectional modulation effect of the SPUDT on the propagation direction of acoustic waves. In the simulation and measurement of the S_{21} parameter (Fig. c), the SPUDT delay line has a bandwidth reduced by nearly half compared to the IDT delay line, but its insertion loss is decreased by approximately 2 dB. Lower insertion loss indicates reduced propagation loss of acoustic waves, enabling more input energy to be converted into effective acoustic energy that participates in the acoustoelectric effect. In the comparison of acoustic-driven current output characteristics (Fig. d), at a 10 cm wireless transmission distance and input power ranging from 0 to 23 dBm, the output current of the SPUDT delay line structure is consistently higher than that of the IDT delay line structure, with the difference increasing significantly as input power rises. At an input power of 10 dBm, the output current of the IDT delay line-based acoustic-driven current source is 0.71 μA, while that of the SPUDT delay line-based counterpart is 1.01 μA, corresponding to an approximate 42% increase relative to the former.

By introducing the SPUDT structure, the energy loss induced by bidirectional acoustic wave propagation is effectively mitigated. Experimental results demonstrate that the SPUDT structure significantly enhances the output current of the acoustic-driven

current source, thus validating the effectiveness of this structural optimization. The relevant experimental data and analyses have been simultaneously updated in the revised manuscript, specifically in the **Results section ("Different Excitation IDT Schemes", Page 15)** and in the **Methods section ("Device fabrication", Page 20)**.

Comment

6. Authors claim that: "The acoustic-driven current source exhibits an excellent linear relationship between output current and input RF power, with a high correlation".This is thru in wired configuration. This is of little interest for implanted devices.

In wireless configuration, linearity is less good and will depend on several parameters such as distance, noise from surrounding reflections and antenna orientation. In real-life operation, linearity will be even more degraded than shown in Figure 5-e.

Response:

We sincerely thank the reviewer for the valuable comment and suggestion. We fully understand and agree with the concern regarding the linearity of the acoustic-driven current source in wireless configurations, particularly due to RF signal transmission. Wireless transmission can be affected by several external factors, including antenna characteristics, environmental noise, signal reflections, and changes in antenna orientation. These effects may lead to attenuation or distortion of the transmitted signal, thereby reducing the linearity of the output current, which in practical scenarios may be more pronounced than what is illustrated in Fig 5e of the manuscript..

It is important to note that such challenges are common to all wireless current sources and energy harvesting systems, not unique to the proposed acoustoelectric current source. In practice, these issues are typically addressed through rectification circuits and optimized antenna configurations, which compensate for transmission-induced nonlinearity, improve overall signal quality, and enhance the linearity of the output current.

In this study, we experimentally validated the linearity of the current source under wired conditions and provided initial evidence of stable performance in a wireless configuration. These results provide a solid foundation for future optimization of wireless operation, including enhanced antenna design and signal processing strategies to further improve linearity in real-world applications.

Comment

7. For wireless configuration, please specify the state of the art of other technologies such as magnetic induction or capacitive coupling allowing control of the electric current.

Response:

We sincerely thank the reviewer for this insightful suggestion. We understand that the reviewer wishes to compare the proposed acoustic-driven current source with other wireless power transfer technologies that enable electric current control. In the revised manuscript, we have expanded the discussion in the Discussion section to better contextualize our work within the existing technological landscape and to highlight its unique advantages.

Conventional wireless power transfer techniques primarily rely on electromagnetic or mechanical coupling mechanisms, including near-field inductive coupling, mid-field inductive coupling, ultrasound transmission, magnetoelectric coupling, and optical transmission. Each of these methods offers distinct trade-offs in terms of transmission distance, power efficiency, miniaturization capability, and alignment sensitivity.

Near-field inductive coupling: Suitable for larger devices, offering higher power transfer and a transmission range up to approximately 10 cm. This method is robust to misalignment but less suitable for miniaturized systems[1].

Mid-field inductive coupling: Combines the efficiency of short-range transmission with moderate distance capability (a few centimeters), achieving a balance between power and compactness[2].

Ultrasound transmission: Appropriate for small devices implanted at shallow or moderate depths, though typically requiring direct skin contact and offering moderate power efficiency[3].

Magnetoelectric effect: Effective for shallow implants and capable of transmitting energy through air or bone, supporting relatively higher power output[4].

Optical transmission: Suitable for ultra-miniaturized and shallow implants, but limited by the strict alignment requirement between transmitter and receiver[5].

In contrast, the acoustic-driven current source proposed in this study introduces a fundamentally different mechanism based on the acoustoelectric effect, where SAWs directly drive charge carriers within a semiconductor layer to generate direct current. This enables a rectifier-free, compact, and easily integrable current source structure. Compared with the aforementioned techniques, it offers better compatibility with semiconductor integration and potential miniaturization advantages.

While the present work does not yet address deep implantation or long-range transmission, it focuses on demonstrating the feasibility and stability of the acoustoelectric current generation mechanism and exploring its biomedical potential. To this end, we have also conducted preliminary in vivo wound healing experiments, where low-intensity (10 μA) acoustically driven stimulation effectively accelerated tissue repair and enhanced healing outcomes, confirming the feasibility of the proposed concept for bioelectronic applications.

These revisions and discussions have been incorporated into the revised manuscript, specifically in the **Discussion section (Page 20)**. Relevant references have been added to strengthen the discussion and highlight the novelty and practical significance of our work.

References:

1. Shin, G. *et al.* Flexible near-field wireless optoelectronics as subdermal implants for broad applications in optogenetics. *Neuron* **93**, 509-521. e503 (2017).
2. Agrawal, D. R. *et al.* Conformal phased surfaces for wireless powering of bioelectronic microdevices. *Nature biomedical engineering* **1**, 0043 (2017).
3. Piech, D. K. *et al.* A wireless millimetre-scale implantable neural stimulator with ultrasonically powered bidirectional communication. *Nature biomedical engineering* **4**, 207-222 (2020).
4. Singer, A. *et al.* Magnetoelectric materials for miniature, wireless neural stimulation at therapeutic frequencies. *Neuron* **107**, 631-643. e635 (2020).
5. Cortese, A. J. *et al.* Microscopic sensors using optical wireless integrated circuits. **117**, 9173-9179 (2020).

Revised:

1. "Compared with conventional wireless power transfer technologies such as inductive[29,30], magnetoelectric[31], and optical coupling[32], the proposed acoustic-driven current source introduces a fundamentally different mechanism based on the acoustoelectric effect. In this approach, SAWs directly drive charge carriers within

a semiconductor layer to generate direct current, without requiring rectification circuits. This not only simplifies the device structure but also facilitates integration with semiconductor components, offering new possibilities for miniaturized and multifunctional wireless systems." (Page 15)

Reviewer: 3

Comment

1. The similar device structures (elastic waves propagating in semiconductor and electro-acoustic effect) have been studied in recent publications, [Nature Communications 12, 2769 (2021)], [Nature Materials 23 (10), 1386-1393 (2024)] and [Nature Electronics 6 (1), 76-85 (2023)]. Beyond the previous work studied how current amplify acoustic waves, this work studies how acoustic waves generate current. Could authors highlight the advances by this work compared to previous work to give readers a more comprehensive background. For example, can you compare electron mobility and sheet carrier density with existing works? And what's the future path towards a larger electron mobility in terms of material stacks?

Response:

1. Novel Perspective on Acoustoelectric Effects

In recent years, the acoustoelectric effect has attracted considerable attention in semiconductor device research. Most existing studies have been focused on how electrical currents can amplify acoustic waves in literature, e.g., Nature Communications 12, 2769 (2021); Nature Materials 23(10), 1386–1393 (2024); and Nature Electronics 6(1), 76–85 (2023). In contrast, our study adopts a distinct and novel perspective by investigating how SAWs can be applied to generate electrical currents. This shift in focus from signal processing and amplification to the creation of a functional current source opens up distinct application avenues in micro-power sources and bioelectronics. This shift in focus represents an innovative contribution to the field.

By examining the interaction between SAWs and semiconductor materials, we explore in depth on how the acoustoelectric effect can drive directional carrier motions, thereby producing a net current. Inspired by the suggestions from the reviewers, the optimized design of acoustic-driven current sources proposed in this work can also significantly enhance the acoustoelectric current, which represents a key distinction from the earlier studies. This work provides new perspectives and experimental evidence for current generation via acoustoelectric coupling and opens up new avenues for application in microelectronics, sensors, and related technologies.

2. Advancement in Carrier Mobility and Surface Carrier Density

Our study employs InSb thin films, which exhibit significantly higher carrier mobilities

than many commonly used semiconductors such as silicon and GaAs. Furthermore, we have adopted a SiO_x/InSb/InAlSb multilayer structure to optimize the electrical properties of the film. It is noteworthy that this level of performance was achieved using magnetron sputtering, a cost-effective and scalable fabrication method, contrasting with the high-cost epitaxial techniques often employed in high-performance RF device studies[1-3]. Compared with the previous study[4], where InSb was directly sputtered onto $LiNbO_3$ (with a carrier mobility of 1000 $cm^2V^{-1}s^{-1}$), our study demonstrates that there is a notable improvement in carrier mobility, reaching 1700 $cm^2V^{-1}s^{-1}$, which directly enhances the acoustoelectric current generation. These results provide the stronger support for the practical use of acoustoelectric devices.

3. Future Directions in Material Engineering

To further improve electron mobility, future studies may explore the use of two-dimensional materials, such as graphene or transition metal dichalcogenides, in place of conventional 3D semiconductors. These materials possess superior electronic transport properties and can be more easily integrated into thin-film structures, potentially enhancing the performance of acoustoelectric devices. Additionally, optimizing the multilayer stack by refining interfacial quality and crystallographic alignment between layers may also improve carrier mobility and overall device performance. For instance, in the Si_3N_4/graphene/InGaAs multilayer structure, the InGaAs transition layer alleviates the lattice mismatch between $LiNbO_3$ and graphene; charge carriers achieve high-speed transport in the graphene layer; the topmost Si_3N_4 serves as a passivation layer, which not only isolates environmental impurities to protect graphene, but also allows partial nitrogen atoms to slightly penetrate into the interface between graphene and InGaAs during its fabrication process to neutralize the dangling bonds at the interface, thereby indirectly improving the interfacial charge carrier transport efficiency. Future optimization will focus on such practical material stacks that can be compatible with cost-effective fabrication processes, thereby enhancing device performance without compromising applicability.

We have incorporated these comparative analysis and discussions in the revised manuscript to better highlight the innovation of our work in material selection, structural design, and the novel application of the acoustoelectric effect in the **Results section ("Material platform overview", Page 4)**. We appreciate the reviewer's insightful suggestions, and we will further refine the study with both simulation and experimental improvements.

References:

1. Hackett, L. *et al*. Towards single-chip radiofrequency signal processing via acoustoelectric electron–phonon interactions. *Nature Communications* **12**, 2769 (2021).
2. Hackett, L. *et al*. Giant electron-mediated phononic nonlinearity in semiconductor–piezoelectric heterostructures. *Nature Materials* **23**, 1386-1393 (2024).
3. Hackett, L. *et al*. Non-reciprocal acoustoelectric microwave amplifiers with net gain and low noise in continuous operation. *Nature Electronics* **6**, 76-85 (2023).
4. Yamanouchi, K. & Shibayama, K. Monolithic elastic surface wave amplifiers of continuous operation. *Japanese Journal of Applied Physics* **16**, 493-496 (1977).

Comment

2. What's the condition of the current being measured? Is the two ends of semiconductor shorted? i.e. set output voltage to 0 in Keithley, 2450 when measuring the current.

Response:

Thank you for your insightful comment. In our study, the measurement conditions were clearly defined to ensure that the observed current originated exclusively from the acoustoelectric effect. Specifically, the two terminals of the semiconductor were not short-circuited, and the output voltage was set to 0 V during the measurements. These conditions were carefully controlled to eliminate any influence from externally applied bias. We have clarified this setup more explicitly in the revised manuscript to ensure that the measurement configuration is fully transparent to readers. We sincerely appreciate the reviewer's valuable suggestion.

Revised:

1. "The AE current was measured using the source measurement unit (Keithley, 2450), configured with the output voltage set to 0 V." (Page 22)

Comment

3. Regarding Fig. 4(f), I failed to understand how an impedance analyzer being used to characterize the impedance in this case. It seems the authors are trying to claim the

output impedance of their current source. If I understand correctly, impedance analyzer will generate signal at certain frequency and measures the voltage and current at given frequency over the device under test (DUT). Therefore, what measured is the impedance of the semiconductor at RF frequency, not the output impedance of current source. To characterize output impedance, authors should give the current-voltage (IV) curve of different loads (e.g. R=0, 10, 100, 1000 Ohm) connected to the source. Also what's the open voltage of the source. Alternative, the Keithley, 2450 used by authors could also perform a IV measurement.

Response:

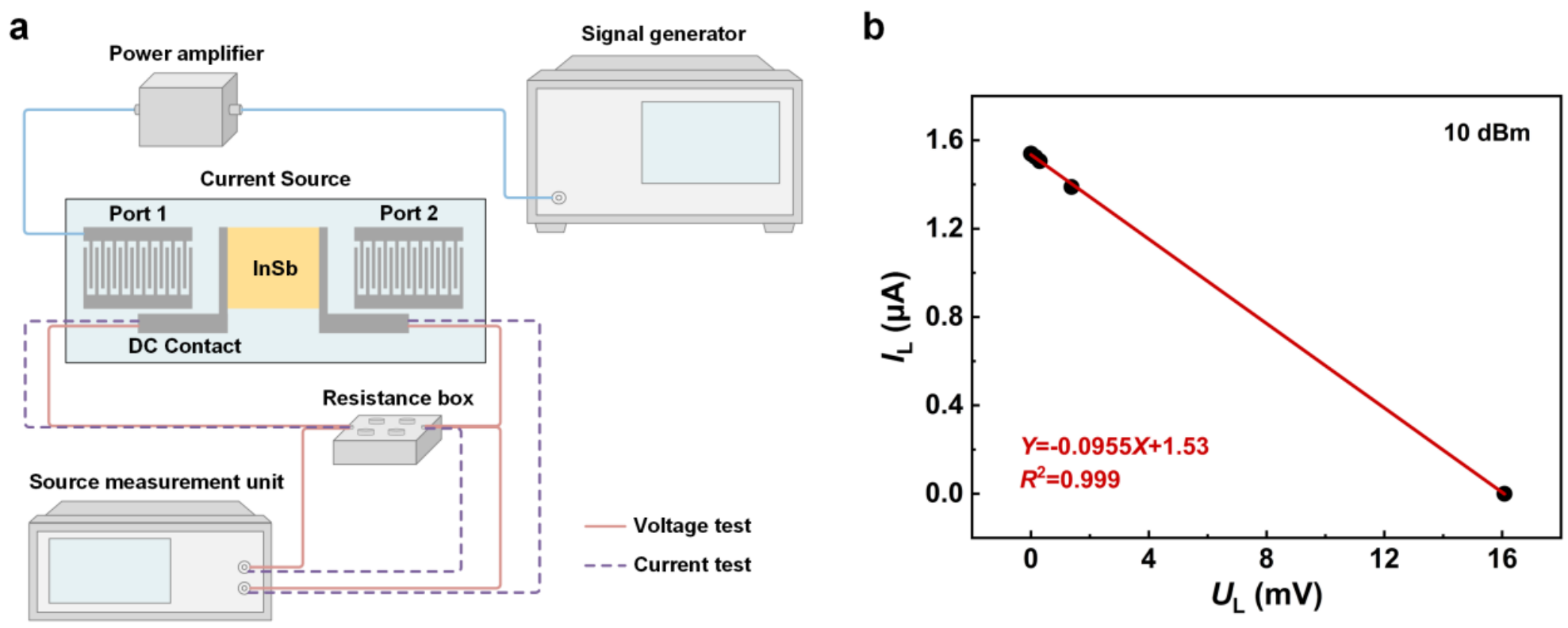

Fig. 7 Output impedance of the acoustic-driven current source. **a** Schematic diagram of the test system. **b** IV measurement curve

We appreciate your careful review of the experimental methods and your professional suggestions, which are of great significance for enhancing the rigor of this paper. Regarding the impedance characterization issue in manuscript Fig. 4f, we have provided the following explanations and improvements based on your comments:

You correctly pointed out that the impedance measured by the impedance analyzer in the original manuscript represents the intrinsic impedance characteristics of the semiconductor thin film in the RF band, rather than the DC output impedance of the current source. By definition, the output impedance R_{out} a current source should be characterized using the load characteristic method, i.e., measuring the relationship between the terminal voltage U_L and the output current I_L under different load resistances R_L. To this end, we conducted supplementary tests using a Keithley 2450 source meter (Fig. 7a), with the specific method detailed as follows:

The input power was fixed at 10 dBm, and R_L was set to 0 Ω, 10 Ω, 100 Ω, 200 Ω, and 1000 Ω in sequence. When R_L = 0 Ω, the measured short-circuit current I_{sc} was 1.54

μA; when R_L approaches infinity, the measured open-circuit voltage U_{oc} was 16.1 mV. By fitting the linear relationship (Fig. 7b), the slope of the fitted curve was determined to be -0.0955, corresponding to an output impedance of 10.5 kΩ.

Compared with RF impedance measurement, this method more directly reflects the internal resistance characteristics of the current source in practical application scenarios. However, we note that there is still room for improvement in the internal resistance of 10.5 kΩ. Future research will focus on optimizing the thin film growth process, using interface passivation layers to reduce the defect density at the heterojunction interface, and lowering the carrier concentration of the semiconductor thin film to achieve an increase in internal resistance.

We have added the above-mentioned test methods, data, and analyses to the **Results section ("Electrical performance of the current source", Page 10)** of the manuscript, and corrected the ambiguous description of the original impedance measurement to clearly distinguish the different physical meanings of "semiconductor RF impedance" and "current source output impedance". The relevant experimental data have also been updated to **Supplementary Note VII** and **Supplementary Fig. 7** for readers' reference.

Once again, we thank you for your accurate suggestions, which have deepened our understanding of the characterization methods for current sources. If there are any other points that need clarification, we are ready to provide further explanation.

Comment

4. I feel the demonstrated power supply seems to be a voltage source, as the force on electrons given by acoustic waves will be balanced by the electric field cross the semiconductor. From band structure, the acoustic waves will deform the band and create a local electrical potential gradient. For sure, once the authors could provide I-V curve of output, we can easily tell if it is a current source or a voltage source.

Response:

We appreciate your thoughtful consideration of this manuscript and your valuable suggestions. Regarding the issue of "identifying the power source type", we provide the following explanations based on physical mechanisms and experimental data:

1. Theoretical Analysis of the Power Source Type

The mechanism of the semiconductor thin-film current source driven by surface acoustic waves can be distinguished from that of a voltage source by the following two aspects:

(1) Carrier transport theory: SAWs propagate along the surface of piezoelectric substrates in the form of elastic vibrations. The strain field induced by these elastic vibrations polarizes the substrate material, thereby generating a moving piezoelectric potential field. Since the piezoelectric potential field migrates synchronously with the SAWs, the charge carriers in the semiconductor thin film, while being accelerated by the potential gradient, are trapped and dragged by the migrating potential wells, which results in a continuous directional current. This process differs from directly utilizing the piezoelectric potential field as an output voltage source[1].

(2) High internal resistance and constant-current, variable-voltage characteristics: The semiconductor thin film is made of high-resistivity materials. Under normal circumstances, the internal resistance of the device R_I is much larger than the external load resistance R_L. In this case, the output current is approximately equal to the short-circuit current and barely affected by R_L, while the voltage varies with the load. This is completely opposite to the characteristics of an ideal voltage source, which has zero internal impedance and exhibits "constant voltage with variable current".

2. Experimental Evidence

Fig. 7b has provided the results of IV characteristic measurements, and the data indicate the following:

(1) Constant output current: When R_L increases from 0 Ω to 10 Ω, the output current I_{out} remains basically stable at 1.54 μA, with a fluctuation of less than 1%. This is highly consistent with the definition of a current source as having a "constant output current". According to circuit theory, I_{out} of an ideal current source is independent of R_L; for a practical current source, since R_I is much greater than R_L, I_{out} is approximately equal to the short-circuit current I_{sc}.

(2) High internal resistance: The internal resistance of an ideal current source tends to infinity to ensure that the output current does not change with the load. Based on the linear fitting of the relationship $U_L=U_{oc}-I_L R_{out}$, the output impedance of the device is calculated to be 10.5 kΩ, which meets the requirement that the internal resistance is much larger than the load resistance.

(3) Load-dependent voltage: When R_L increases from 100 Ω to 200 Ω, the output voltage linearly increases from 150 μV to 297 μV. This is a typical characteristic of a current source, where the output voltage linearly varies with load resistance, whereas the output voltage of a voltage source remains essentially constant.

In conclusion, whether from the perspective of physical mechanisms or experimental data, this device conforms to the definition of a current source. We have added a detailed analysis of the IV curve to the **Results section ("Electrical performance of the current source", Page 10)** of the manuscript and clearly emphasized the current source characteristics in the abstract and conclusion sections to avoid misunderstandings among readers. Thank you for your suggestions, which have helped us further clarify this scientific issue!

References:

1. Carmichael, C. P., Smith, M. S., Weeks, A. R. & Malocha, D. C. Experimental investigation of surface acoustic wave acoustoelectric effect using a graphene film on lithium niobate. *IEEE Transactions on Ultrasonics, Ferroelectrics, and Frequency Control* **65**, 2205-2207 (2018).

Comment

5. How is noise floor in Fig. 4c calculated? More details are needed.

Response:

We sincerely thank the reviewer for this valuable comment. The following provides a detailed explanation of how the noise floor shown in manuscript Fig. 4c was calculated:

1. Data Preparation and Filtering

The acquired current signal was first processed using a 10 Hz low-pass filter to remove high-frequency noise components and preserve the relevant low-frequency noise.

2. Fourier Transform and Frequency-Domain Analysis

A Fast Fourier Transform (FFT) was applied to convert the filtered time-domain signal into the frequency domain. The resulting frequency spectrum represents the signal amplitude as a function of frequency, allowing us to identify noise characteristics across the spectrum.

3. Noise Power Spectral Density (PSD) Calculation

The noise power spectral density (PSD, in A^2/Hz) was estimated using the periodogram method applied to the AC component of the current signal (after DC offset removal). This yields the distribution of noise power over frequency.

4. Conversion to Noise Density

The square root of the PSD was taken to obtain the current noise density (in $A/Hz^{-1/2}$), which represents the noise amplitude per unit bandwidth and provides a more intuitive measure of current noise.

5. Noise Density Extraction at a Specific Frequency

For quantitative analysis, the noise density value at 5 Hz was extracted from the frequency spectrum. The index corresponding to the 5 Hz bin was determined based on the FFT frequency resolution.

6. Visualization and Definition of the Noise Floor

The noise density spectrum was plotted on a logarithmic scale to clearly display its frequency-dependent behavior. The value near 5 Hz was highlighted as the representative indicator of the system's noise floor.

In summary, the noise floor was determined by:

(1) transforming the time-domain signal to the frequency domain via FFT;

(2) estimating the PSD using a periodogram;

(3) taking the square root of the PSD to obtain the noise density; and

(4) extracting the noise density at 5 Hz for quantitative comparison.

This procedure enables a precise evaluation of the current source's noise characteristics and its frequency-dependent stability. We hope this clarification adequately addresses the reviewer's concern.

Comment

6. Why is 5 Hz chosen to analysis your noise density? Can you please also show the reader the full noise spectrum of your current source at different output level? Is there 1/f noise? If so, what’s the cut off frequency of 1/f noise.

Response:

We sincerely thank the reviewer for these constructive comments. The reasons for selecting 5 Hz for noise density analysis and the discussion regarding 1/f noise are clarified as follows.

1. Reason for selecting 5 Hz

According to the Nyquist–Shannon sampling theorem, the upper limit of frequency analysis is half of the sampling frequency. In our measurements, the sampling frequency of the source measurement unit was set to 12.5 Hz, resulting in a current frequency analysis upper limit of 6.25 Hz. Within this range, 5 Hz was selected as the representative frequency for noise density evaluation because it provides a stable indicator of current fluctuation while minimizing the influence of instrument-induced high-frequency noise. Increasing the sampling frequency would introduce additional parasitic noise from the instrument and cables, thereby reducing measurement accuracy in the low-frequency range.

2. Full noise spectrum under different RF powers

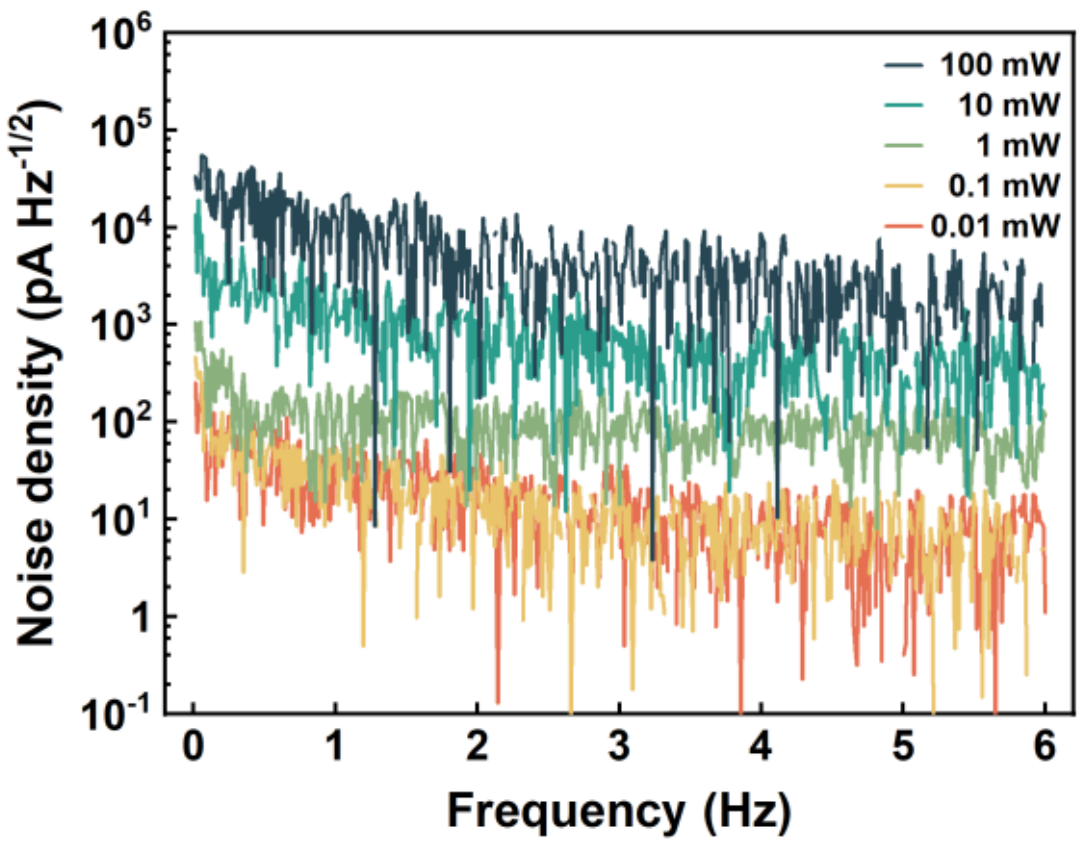

Fig. 8 Current noise density spectra of the acoustic-driven current source under different RF input powers.

To further investigate the noise characteristics of the acoustic-driven current source, we analyzed the current noise spectra under different RF input powers (0.01 mW, 0.1 mW, 1 mW, 10 mW, and 100 mW), as shown in Fig. . The noise density consistently exhibited a decreasing trend with increasing frequency, and the 5 Hz point was used as a representative reference for quantitative comparison across all input power levels.

3. On the presence of 1/f noise

We acknowledge that 1/f noise often dominates in low-frequency regimes. However,

due to the limited measurement bandwidth (0.02-6.25 Hz) and the focus on overall current stability, the present study did not quantitatively separate 1/f noise from other possible components such as thermal or shot noise. While a general downward trend in noise density with increasing frequency was observed, this alone was insufficient to conclusively confirm 1/f behavior. Further investigation over an extended frequency range will be considered in future work.

In summary, 5 Hz was selected as a representative low-frequency reference for consistent comparison, and the full noise spectra at different RF input powers were analyzed to ensure the reliability of the results. The potential presence of 1/f noise is acknowledged but was not quantitatively verified within the current measurement range.

Comment

7. Is the antenna an off-chip component? Is it possible to have it integrated on chip? Please articulate a technical solution to fully integrated current source using the proposed technique, if possible.

Response:

In the current design, the antenna is indeed implemented as an external component which was connected to the current source and enabled wireless power transmission. However, in light of the increasing demands for system miniaturization and higher integration, we have explored the feasibility of integrating the antenna directly onto the chip. Below is an overview of the proposed integration scheme and its technical feasibility discussions.

1. Challenges in Antenna Integration

(1) Size-Frequency Matching: One of the primary challenges in the antenna integration is aligning the antenna's physical dimensions within the limited chip area while ensuring effective radiation performance at the desired operational frequency.

(2) Material Constraints: Conventional antennas typically use metals such as copper or silver, whereas integrated circuits mainly involve semiconductors or conductive thin films. This material mismatch can affect both radiation efficiency and electromagnetic wave propagation.

To address the aforementioned core challenges, existing technologies have developed multi-dimensional solutions: metamaterial technology enables precise manipulation of electromagnetic waves by means of artificially designed subwavelength unit arrays, which can break the inherent proportional constraint between the size of traditional antennas and their operating wavelengths;[1] microstrip antenna technology has improved compatibility with radio frequency integrated circuits by optimizing feed structures and substrate dielectric properties;[2] in terms of material system compatibility, low-loss dielectric substrates (e.g., thin-film $LiNbO_3$, quartz) can effectively reduce the confinement effect of electromagnetic waves within the substrate, while conductive functional alternatives such as the transparent conductive film ITO (indium tin oxide) can significantly mitigate process compatibility conflicts between metals and semiconductors.[3-5]

2. Feasible Integration Strategy: Combining the Material of Lithium Niobate with Magnetic Films

In recent years, emerging acoustic wave antenna technologies have significantly overcome the limitations of traditional electromagnetic resonance mechanisms. Among them, SAW-based magnetoacoustic coupling (MAC) devices integrate magnetostrictive thin films with SAW devices, and thereby achieve on-chip electromagnetic radiation through the multi-stage "acoustic-magnetic-electromagnetic" energy conversion pathway. Such devices can realize efficient radiation in the GHz frequency band on the typical $LiNbO_3/SiO_2/Si$ substrate, while maintaining excellent compatibility with CMOS processes.[6]

(1) Advantages of Lithium Niobate Substrate: Lithium niobate ($LiNbO_3$) exhibits excellent piezoelectric properties and is widely used in SAW devices. By employing $LiNbO_3$ as the substrate and leveraging the acoustoelectric effect, it is possible to generate current efficiently within the thin film. Therefore, it is not only served as the active substrate for driving the current source but may also partially replace conventional antennas, facilitating the integration of the current source and antenna.

(2) Role of Magnetic Thin Films: To achieve wireless signal transmission, we propose depositing magnetic thin films (e.g., ferrite films) onto the $LiNbO_3$ substrate. These magnetic films can effectively couple with acoustic waves and exhibit favorable electromagnetic radiation properties, allowing them to function as integrated antennas. This approach enables both the antenna and current source to co-exist on a single chip, eliminating the need for external antenna components.

(3) Integration Method: Magnetic films can be deposited onto the $LiNbO_3$ substrate using techniques such as evaporation, sputtering or chemical vapor deposition (CVD). This ensures close coupling between the current source and antenna while simplifying the system structure by sharing material layers.

3. Technical Advantages

By integrating the antenna and current source on a single chip, the system's integration level can be significantly enhanced, reducing the critical needs for external interconnections and components. This in turn improves the overall system reliability and operational stability.

In summary, integrating the antenna with the current source using the $LiNbO_3$ substrate and magnetic thin films is technically feasible. This approach not only increases integration density but also reduces reliance on external components while improving the efficiency of wireless power transfer and signal reception. We believe this integration strategy holds a great potential for applications in highly integrated, low-power microelectronic systems.

References:

1 Ghasri, H., Nourinia, J., Ghobadi, C., Lalbakhsh, A. & Mohammadi, B. Compact Triple-band Metamaterial Antenna. *In 2021 Photonics & Electromagnetics Research Symposium (PIERS)*, 675-680 (2021).

2 Basavaraju, D. R. & Sukumar, R. Design and Analysis of Microstrip Patch Antennas for Sub-6GHz 5G: A Comparative Study of Substrates and Feeding Techniques. *SSRG International Journal of Electronics and Communication Engineering* **11**, 206-218 (2024).

3 Tao, Z., Wang, H., Feng, H. *et al*. Ultrabroadband on-chip photonics for full-spectrum wireless communications. *Nature* **645**, 80-87 (2025).

4 Moller de Freitas, M., Zhu, X., Ullah, M.S. *et al*. Monolithically integrated ultra-wideband photonic receiver on thin film lithium niobate. *Communications Engineering* **4**, 55 (2025).

5 Yasuda Y., Yamada, Y., Koshiji, F. *et al*. Effect of annealing treatment of indium tin oxide thin films on film properties and transparent antenna properties. *Thin Solid Films* **794**, 140295 (2024).

6 Fu, Y., Li, J., Hu, R. *et al*. Enhancing Radiation in Magnetoacoustic Coupling SAW Devices via FeGaB Thin-Film and Focused Interdigital Transducers on a $LiNbO_3/SiO_2/Si$ Substrate. *IEEE Transactions on Microwave Theory and Techniques* **73**, 8500-8511 (2025).

Comment

8. It's up to the author, but we are just very interested in if this design is also a good AC current source. For example, if you apply an amplitude modulation (e.g., 10Hz) on the RF signal (434.75MHz), how is the current output? Would you get a 10Hz current output at a similar level to the DC output if RF signal power level is the same? In another word, what is 3dB bandwidth of this AC current source?

Response:

We thank the reviewer for the valuable suggestion. To evaluate whether the proposed device can function as an AC current source, we conducted experiments using amplitude-modulated RF signals and examined the resulting output current (I_{out}). The results confirm that under all tested modulation conditions, the output remains a steady DC signal with no detectable AC component, indicating that the device does not operate as an AC current source. Consequently, the concept of a 3 dB bandwidth is not applicable in this context.

In our experiments, a 433 MHz RF signal was generated using a Stanford Research Systems SG394 source which supports amplitude modulation down to a minimum frequency of 1 kHz. Although the reviewer suggested using a 10 Hz modulation frequency, this limitation does not affect the validity of our conclusions.

To comprehensively investigate this question, we applied 1 kHz amplitude-modulated signals with various waveforms—including sine, sawtooth, triangle, square, and noise—to the RF input. The modulated signals were amplified to 20 dBm and applied to the device. The corresponding I_{out} was measured using a source measurement unit (Keithley 2450). Representative waveforms of the modulated input signals are shown in Fig. 4, and the measured I_{out} values are presented in Fig. 5.

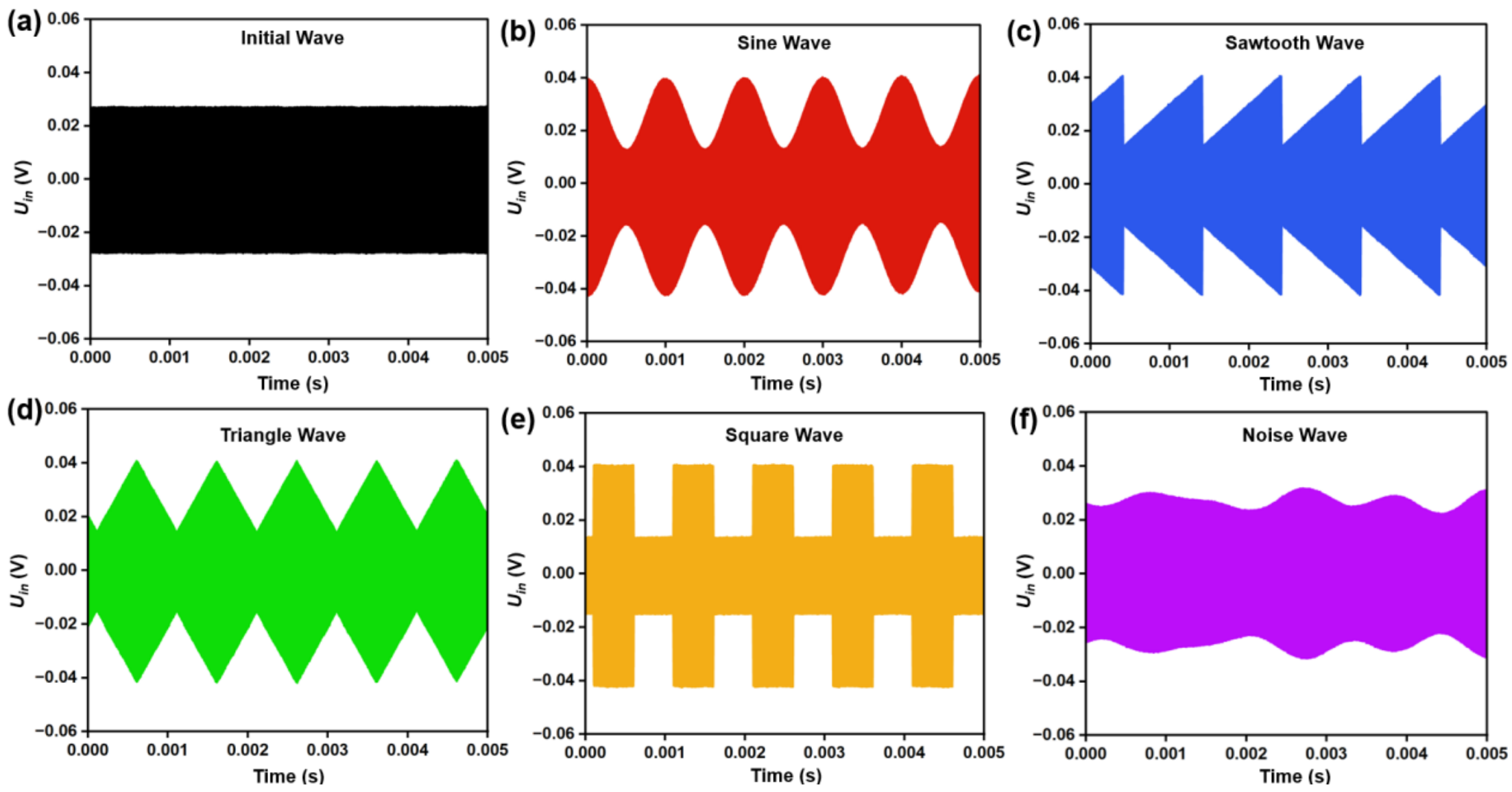

Fig. 4 Input RF signal waveforms under different amplitude modulation conditions: **a** unmodulated, **b** sine, **c** sawtooth, **d** triangle, **e** square, and **f** noise.

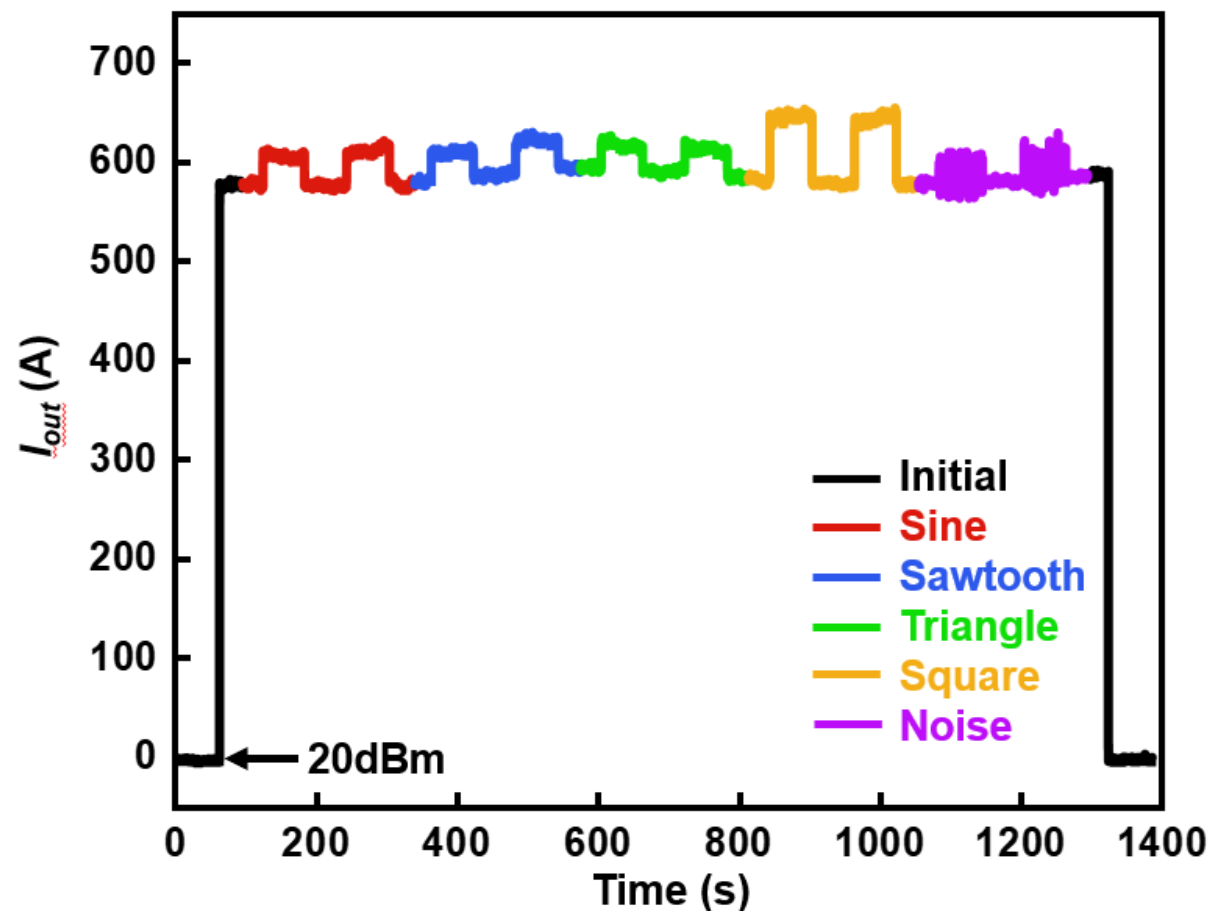

Fig. 5 Measured I_{out} under different amplitude-modulated input signals.

As shown in Fig. 5, the output current exhibits a slight increase in magnitude under amplitude-modulated excitation, which can be attributed to the higher peak amplitude of the modulated RF input. However, no oscillatory behavior or polarity reversal was observed in I_{out}—indicating that the output current remains unidirectional and DC in nature.

To further confirm this, we analyzed the frequency spectrum of I_{out} using a frequency analyzer (Fig. 6). Across all tested modulation types, no spectral peak appears at the modulation frequency (1 kHz), indicating that the output remains purely DC with no detectable AC component.

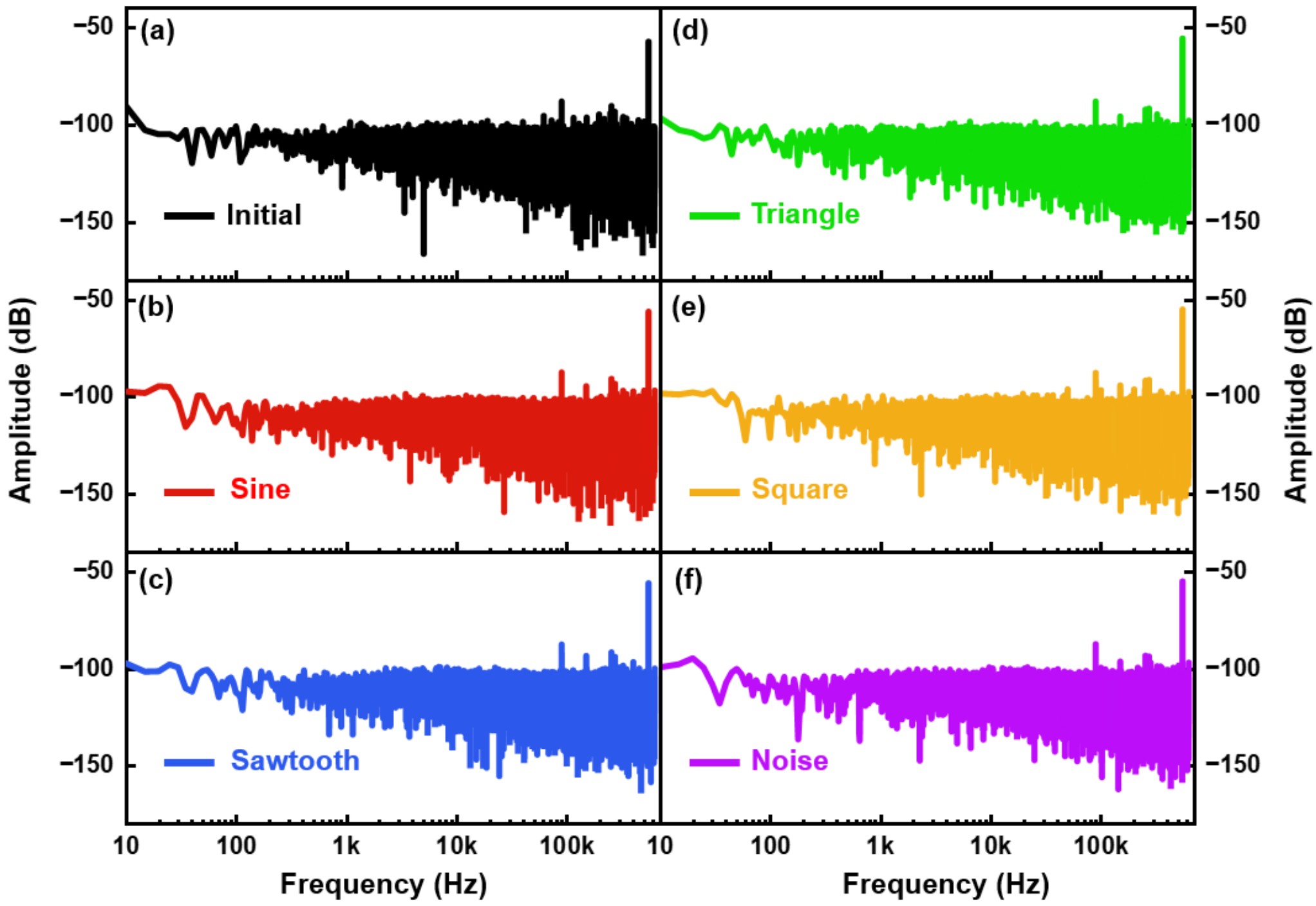

Fig. 6 Frequency spectra of I_{out} under different amplitude modulation conditions: **a** unmodulated, **b** sine, **c** sawtooth, **d** triangle, **e** square, and **f** noise.

In summary, amplitude modulation of the RF input does not produce an AC-modulated output current. The output current remains a stable DC signal, and thus, a 3 dB bandwidth for AC operation cannot be defined for this device.

Reviewer: 4

Comment

1. The RF sputtering of 250 nm thick InSb results in significant R-G center formation, surface defects, and interdiffusion, particularly in lightly-doped semiconductors. The manuscript should provide a more detailed explanation of the generation of electron-hole pairs, their movement induced by acoustic forces, their creation and recombination, and their flow toward metal barriers (need a schematic for explanation). Additionally, the paper should clarify why vibrational energy at RF 433 MHz is converted into DC current.

Response:

We sincerely appreciate your valuable comments, which are of great significance for enhancing the academic rigor of the manuscript. The core function of the acoustic-driven current source is to realize the conversion from radio frequency (RF) electrical energy to mechanical energy and subsequently to direct current (DC) electrical energy. In response to your questions, we have systematically organized the working mechanism of the acoustic-driven current source based on the research content[1-3], as illustrated in Fig. 7.

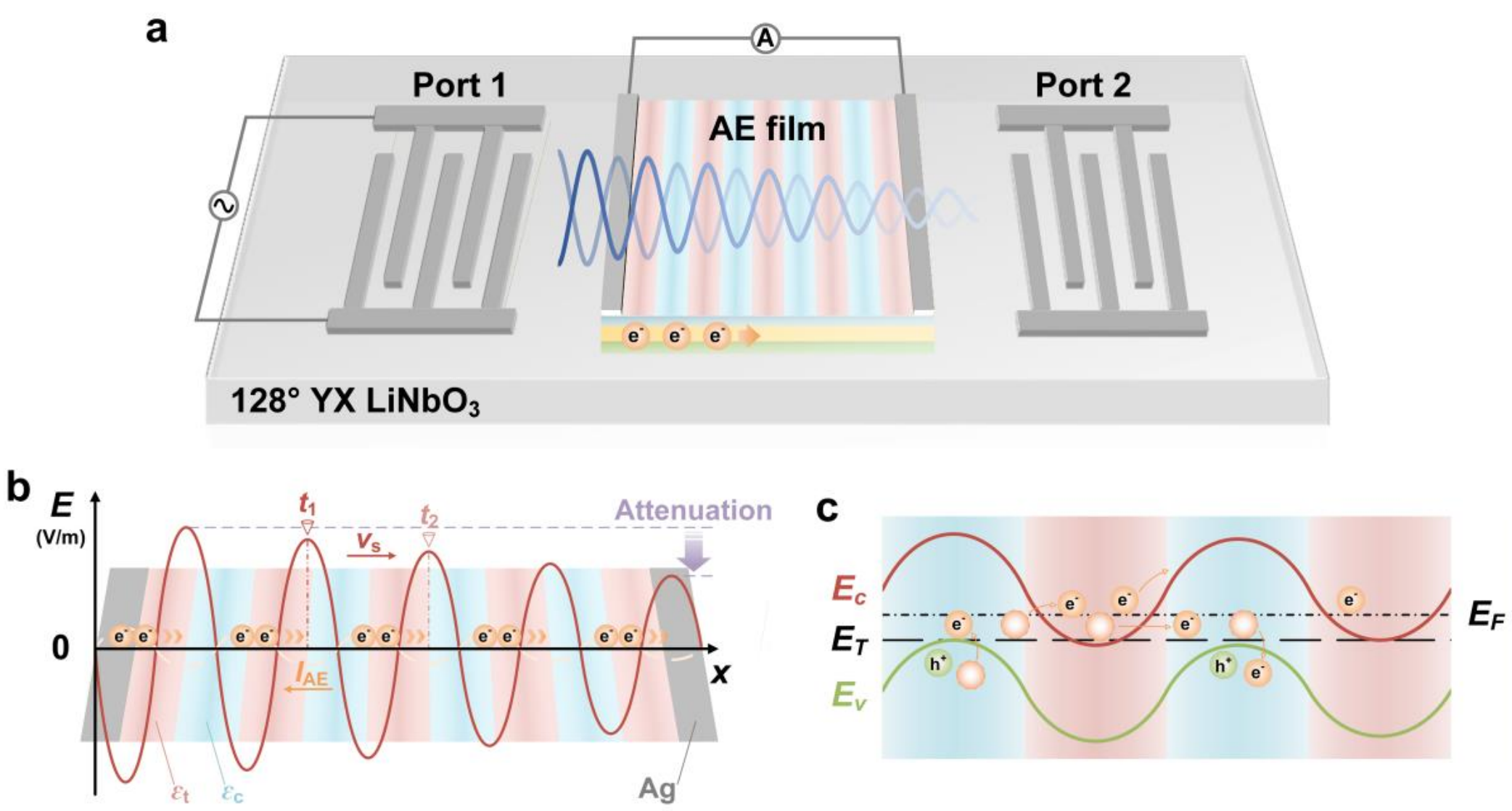

Fig. 7 Device structure and operating mechanism of the acoustic-driven current source. **a** Schematic diagram of the device structure. **b** Directional transport of electrons under the traveling-wave piezoelectric polarization field. The red solid line denotes the magnitude and direction of the piezoelectric polarization field, while the light orange dashed line represents those of the internal electric field. ε_t and ε_c represent tensile strain and compressive strain, respectively. **c** Processes of

generation, movement and recombination of electron-hole pairs, where E_T is the energy level of SRH centers.

The specific process is as follows:

1. Device Structure and Composition

(1) Piezoelectric substrate ($LiNbO_3$) and interdigital transducer (IDT): The input IDT receives radio frequency (RF) electrical signals, which are converted into surface acoustic waves (SAWs) via the inverse piezoelectric effect to propagate along the substrate surface. Conversely, the mechanical energy of SAWs can be converted back into electrical energy through the direct piezoelectric effect, serving as one of the fundamental conditions for AE conversion.

(2) AE film ($SiO_x/InSb/In_{0.6}Al_{0.4}Sb$): The InSb layer is the key functional layer for AE conversion, as it converts the mechanical energy of SAWs into the directional transport kinetic energy of carriers through AE coupling. During magnetron sputtering deposition, numerous intrinsic defects (e.g., indium interstitials, antimony vacancies) and complex defects (Shockley-Read-Hall centers, SRH) form in the InSb layer. Among them, intrinsic defects serve as the primary sources of single-type carriers, while SRH centers act both as the main generation pathway for electron-hole pairs (e^--h^+) and as the core sites for carrier recombination.

(3) Metal electrodes (Ag): The Ag electrodes are fabricated by vacuum thermal evaporation. Due to the similar work functions of Ag and InSb, stable ohmic contacts are formed at both ends of the InSb film, providing channels for the directional transport of carriers and DC output.

2. Dynamic Characteristics of the Traveling-Wave Piezoelectric Polarization Field

Upon the traveling-wave propagation of SAWs along the substrate, periodic lattice strain is induced in the substrate's surface region. Owing to the piezoelectric polarization effect of $LiNbO_3$, this periodic lattice distortion generates a periodic electric field distribution both in and on the substrate surface—specifically, a traveling-wave piezoelectric polarization field that propagates synchronously with the SAWs. Its amplitude and direction exhibit periodic variations with spatial position (with the period matching the SAW wavelength), while the peak position travels along the propagation direction at the SAW phase velocity. Equivalent to a dynamically moving electric field, it can exert a continuous directional driving force on the carriers in the semiconductor layer.

3. Generation, Directional Motion, Recombination Equilibrium, and Effective Electron Accumulation of Carriers

(1) Generation of Carriers

Indium interstitials, as donor defects, undergo ionization to release a large number of free electrons into the conduction band. Conversely, antimony vacancies, acting as acceptor defects, ionize to release holes. However, the formation density of indium interstitials is substantially higher than that of antimony vacancies, leading to a significantly greater number of electrons compared to holes.

In addition, SRH center-assisted enhanced excitation constitutes the primary pathway for e^{-}-h^{+} generation. The InSb film fabricated via magnetron sputtering contain a high density of SRH centers—defects that introduce midgap levels within the band gap, significantly lowering the energy barrier for carrier excitation. The energy imparted by SAWs not only excites electrons trapped at SRH centers to the conduction band but also facilitates the transition of valence band electrons to SRH centers, with an efficiency substantially higher than that of intrinsic excitation.

(2) Directional Motion of Carriers

The traveling-wave piezoelectric polarization field generated by the substrate penetrates into the InSb film. Free carriers in the InSb film are electron-dominated. Under the electric field force of this polarization field, electrons are dragged forward by the moving electric field, achieving directional drift along the SAW propagation direction. During this process, the mechanical energy carried by SAWs is transferred to carriers via the electric field force of the piezoelectric polarization field, converting into the directional kinetic energy of carriers. Meanwhile, the spatial concentration gradient induced by carrier directional drift induces an internal electric field. This internal electric field is opposite to the piezoelectric polarization field and partially offsets its effect, but the directional drift trend of carriers remains dominant overall.

(3) Recombination Equilibrium and Effective Electron Accumulation of Carriers

Carrier generation (G) and recombination (R) proceed synchronously during motion, with dynamic equilibrium regulated by SRH centers. On one hand, the continuous energy supply from SAWs renders the generation rate significantly higher than the intrinsic generation rate. On the other hand, electrons and holes that are not promptly transported recombine through SRH centers. Since newly generated electrons are capable of rapidly escaping the recombination region by virtue of their high mobility,

thereby minimizing recombination-related losses, whereas holes exhibit inherent difficulty in departing from the recombination region due to their low mobility, effective electrons competent for directed transport are persistently retained, laying a foundation for the formation of macroscopic AE current.

4. Carrier Transport through Ohmic Contacts: DC Output

Owing to the similar work functions between Ag electrodes and the InSb film, stable ohmic contacts are formed. This contact characteristic eliminates the carrier transport barrier, enabling directionally moving carriers to be continuously output through the electrodes. Ultimately, the AE current is converted to stable DC electrical energy.

5. Influence of Operating Frequency on AE Current

AE current can be generated across various frequencies of SAWs, with 433 MHz identified as the resonant center frequency of the device. At this frequency, the acoustic wave intensity reaches its maximum; with increasing acoustic wave intensity, the energy imparted to carriers increases correspondingly, which in turn enhances the AE current. Therefore, this paper focuses on examining the AE current characteristics of the device at this center frequency.

In summary, the AE effect of the acoustic-driven current source arises from the synergistic effect of three processes: defect-assisted efficient carrier generation, AE coupling-mediated directional carrier transport, and efficient DC output through ohmic contacts. It is inherently dependent on the periodic dynamic field properties of SAWs, as well as the high electron mobility and tunable defect characteristics of the InSb film. In the **Results section** of the revised manuscript, a corresponding schematic diagram has been added to manuscript **Fig. 1a**. This diagram intuitively elucidates the overall mechanism of the conversion of RF electrical energy into acoustic energy and further into DC electrical energy, and simultaneously clearly illustrates the directional transport process of carriers driven by acoustic waves.

References:

1. Bhattacharjee, P., Mishra, H., Iyer, P. K. & Nemade, H. B. Acoustic interactions with semiconductors: progression from inorganic to organic material system. *Journal of Physics D-Applied Physics* **57**, 423001 (2024).

2. Nie, X. C. *et al*. Surface acoustic wave induced phenomena in two-dimensional materials. *Nanoscale Horizons* **8**, 158-175 (2023).

3. Hernández-Mínguez, A., Liou, Y. T. & Santos, P. V. Interaction of surface acoustic waves with electronic excitations in graphene. *Journal of Physics D-Applied Physics* **51**, 383001 (2018).

Comment

2. The induced current between the electrodes is also influenced by the piezoelectric properties of the $LiNbO_3$ substrate. The manuscript should explain why current is generated only in the InSb layer (current will also be induced in the LN substrate) and describe the mechanisms involved.

Response:

We sincerely thank the reviewer for the insightful comments regarding the role of the $LiNbO_3$ substrate in the generation of the induced current. We agree that the piezoelectric properties of $LiNbO_3$ play an essential role in forming the accompanying electric field of the SAW, and we now provide a more detailed explanation of why the current is generated only within the InSb layer.

The induced current originates from the acoustoelectric effect. When SAWs propagate on the $LiNbO_3$ substrate, their piezoelectric nature generates a periodic accompanying electric field that penetrates the InSb layer and drives the directional drift of charge carriers. When the carrier drift velocity is lower than the acoustic wave velocity, part of the acoustic energy is transferred to the carriers, resulting in a net current along the propagation direction.

The measurable current arises exclusively within the InSb film. Although $LiNbO_3$ generates the electric field required for acoustoelectric coupling, it lacks free carriers and has a high resistivity, which prevents it from conducting current. In contrast, the InSb thin film possesses high carrier mobility and moderate carrier concentration, allowing efficient energy transfer and acoustoelectric conversion. Thus, current generation occurs only in the InSb layer, while the $LiNbO_3$ substrate serves as the coupling medium without contributing to current conduction.

This conclusion was confirmed by control experiments. When Ag electrodes were fabricated directly on $LiNbO_3$ without the SiO_x/InSb/$In_{0.6}Al_{0.4}Sb$ multilayer, no detectable current was observed. Once the InSb layer was introduced, a significant

acoustoelectric current appeared, confirming that current generation originates from the semiconductor layer rather than the piezoelectric substrate.

Under the periodic electric field accompanying the SAW, the carriers in the InSb film undergo unidirectional drift, forming a stable direct current between the electrodes. The SAW effectively converts acoustic energy into direct current electrical output through carrier–wave interaction.

Overall, the $LiNbO_3$ substrate provides the acoustic and electric field coupling required for the acoustoelectric effect, but due to its insulating nature, it does not directly contribute to current generation. The acoustoelectric current is generated exclusively within the InSb film, where the high carrier mobility enables efficient energy transfer and stable DC output.

Comment

3. The current flow generates a new E-field at both ends of the InSb. The manuscript should explain how this newly generated E-field, which depends on the current magnitude, affects both the piezoelectric properties of the material and the DC current.

Response:

We thank the reviewer for raising this important point. It should be noted that the current itself does not generate an electric field but rather a magnetic field. In our work, Gauss's law is applied under the conventional quasi-static assumption, and the induced magnetic field is not considered.

Although the InSb layer is not piezoelectric, the SAWs excited in the $LiNbO_3$ substrate generate a piezoelectric polarization field that drives carrier motion in the InSb layer. After redistribution, these carriers form an internal electric field that exists throughout the semiconductor layer. This internal field influences the piezoelectric properties of the substrate through a screening effect, which depends on both the driving frequency and the carrier concentration. Specifically, the acoustoelectric interaction involves multiple coupled fields operating at different timescales[1]. The excitation frequency corresponds to a timescale T, whereas the conduction and diffusion frequencies of carriers correspond to timescales T_c and T_d, respectively.

1. When the carrier concentration is relatively high, T_c greatly exceeds the driving period, resulting in a strong screening effect. In this situation, the piezoelectric

polarization field is significantly weakened, because carrier drift responds much faster than the wavefront oscillation, reducing the efficiency of acoustoelectric current generation.

2. Conversely, when the carrier concentration is relatively low, T_c is much smaller than the driving period, and the screening effect is weak. Here, the polarization field remains nearly intact; however, due to the high excitation frequency and short acoustic wavelength, the carrier diffusion–induced smoothing effect becomes significant[1,2], which again suppresses the acoustoelectric current. This explains why a pronounced acoustoelectric current is observed near 433 MHz, where the driving frequency approximately matches the carrier conduction frequency.

Additionally, some boundary effects could, in principle, produce electric fields at the ends of the InSb layer. To avoid this, our device uses vacuum thermal evaporation processing and silver electrodes to ensure ohmic contacts at both ends. No external bias is applied in the wireless configuration, so Schottky or tunneling barriers are effectively eliminated.

References:

1. Hutson, A. R. & White, D. L. Elastic Wave Propagation in Piezoelectric Semiconductors. *Journal of Applied Physics* **33**, 40-47 (1962).
2. Yang, W., Zhang, S., Guo, L. & Hu, Y. Effects of charge carriers on elastic waves in piezoelectric semiconductors. *International Journal of Mechanical Sciences* **291**, 110180 (2025).

Comment

4. More detailed information is needed about the type of InSb, its carrier concentration, carrier generation due to the acoustic wave, and the flow of carriers.

Response:

We sincerely thank the reviewer for these insightful comments. Hall effect measurements confirmed that in the $SiO_x/InSb/In_{0.6}Al_{0.4}Sb$ heterostructure, the $In_{0.6}Al_{0.4}Sb$ buffer layer exhibits p-type conductivity, while the InSb active layer is n-type. The heterostructure demonstrates an electron mobility of 1700 $cm^2/(V{\cdot}s)$, a sheet carrier concentration of 7×10^{11} cm^{-2}, and a sheet conductivity of 1.9×10^{-4} S, which are primarily determined by the InSb layer. Regarding the role of SAWs, they do not

generate additional carriers but instead interact with the pre-existing carriers in the InSb film through the AE effect. This interaction drives the majority carriers (electrons) into directional motion along the acoustic wave propagation, thereby giving rise to the observed AE current.

Revised:

1. "According to the Hall measurements (ECOPIA, HMS-5500), the $SiO_x/InSb/In_{0.6}Al_{0.4}Sb$ heterostructure also shows the highest mobility and the lowest sheet carrier density among all compositions, with a mobility of 1700 $cm^2V^{-1}s^{-1}$, a sheet carrier density of 7×10^{11} cm^{-2}, and a sheet conductivity of 1.9×10^{-4} S at room temperature. These results confirm that the InSb active layer is n-type, whereas the $In_{0.6}Al_{0.4}Sb$ buffer layer exhibits p-type conductivity." (Page 7)
2. "When the acoustic wave propagates as the elastic vibrations on the surface of the piezoelectric substrate, the resulting strain field induces polarization of substrate materials, establishing a mobile piezoelectric potential field. The transient electric field associated with the acoustic waves facilitates charge accumulation, generating measurable AE currents within the film through the interaction between phonons and electrons." (Page 5)

Comment

5. The 500°C, 1-hour annealing process has a significant effect on the piezoelectric polarization characteristics of the LN substrate. A thorough explanation of this impact is required.

Response:

We thank the reviewer for raising this important point. Our experimental results indicate that annealing at 500 °C for 1 hour has no significant impact on the piezoelectric polarization of the 128°YX $LiNbO_3$ substrate or the frequency response of the SAW device.

The annealing was carried out with a heating rate of 5 °C/min, a target temperature of 500 °C, a soaking time of 1 hour, and furnace cooling. To evaluate its effect on the piezoelectric properties, the longitudinal piezoelectric coefficient d_{33} was measured at five different locations on the 128°YX $LiNbO_3$ substrate using a room-temperature d_{33} meter (ZJ-3, Institute of Acoustics, Chinese Academy of Sciences). The measurement results are shown in Table 3. The average d_{33} increased slightly from 19.92 pC/N to

20.42 pC/N, a difference of only 2.5%, indicating that the annealing process had a negligible impact on the piezoelectric performance of the substrate.

Table 3 Measured d_{33} values of 128°YX $LiNbO_3$ before and after annealing (unit: pC/N)

Sample	1	2	3	4	5	Average
Before annealing	19.9	19.9	19.9	20.0	19.9	19.92
After annealing	20.4	20.4	20.5	20.4	20.4	20.42

To evaluate potential changes in the frequency response characteristics of the SAW device, we fabricated devices directly on the LN substrate without any thin-film deposition in the acoustic propagation region and measured their performance before and after annealing, as shown in Fig. 8. The insertion loss increased slightly from 6.12 dB to 7.18 dB, and the center frequency shifted from 433.48 MHz to 433.60 MHz. These minimal changes indicate that the annealing process has negligible impact on the device's frequency response characteristics.

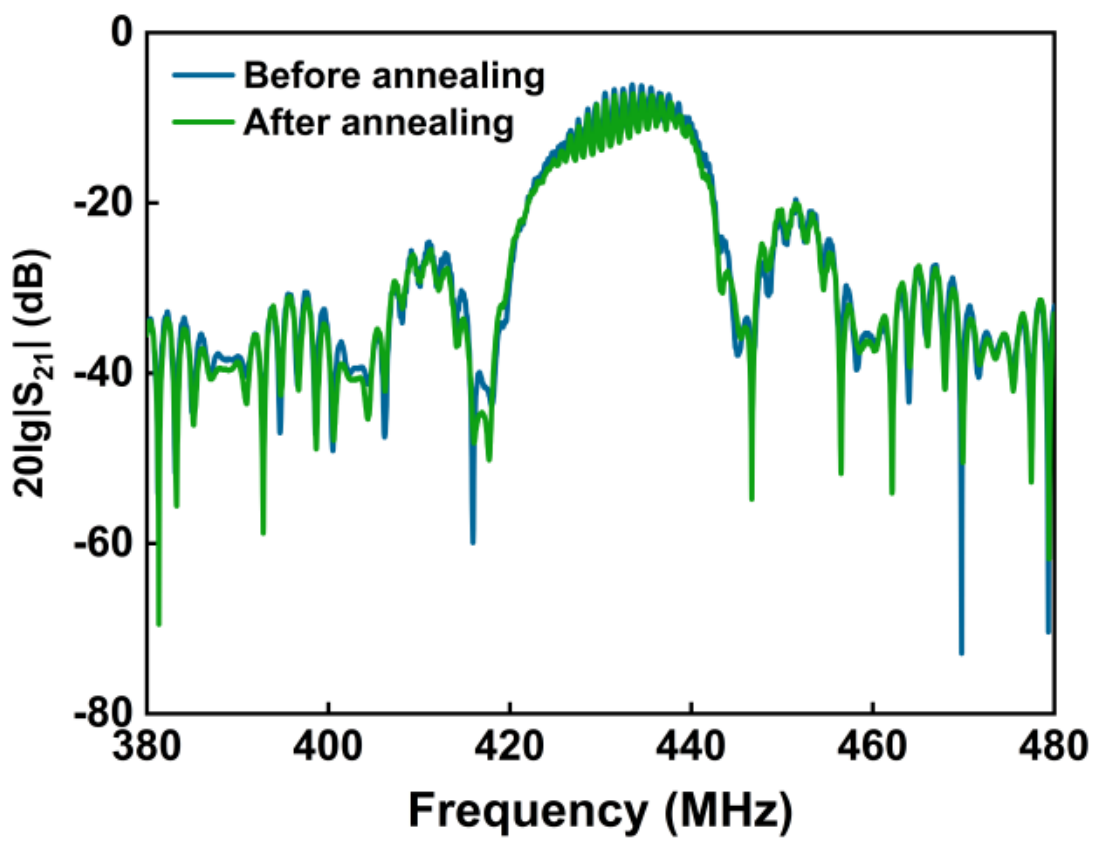

Fig. 8 Frequency response characteristics of the SAW device before and after annealing.

In summary, our experimental results demonstrate that annealing at 500 °C for 1 hour does not cause any significant changes in the piezoelectric polarization characteristics of the 128°YX $LiNbO_3$ substrate or in the frequency response of the corresponding SAW device.

Comment

6. DC current measurements are being performed with a current meter. The manuscript should clarify whether the experiment uses a common ground in the existing setup or a separate ground configuration. This clarification is essential for ensuring experimental

accuracy. Additionally, it should confirm whether any reverse current is caused by the grounding of the test equipment.

Response:

We appreciate the reviewer's attention to the grounding configuration, which is crucial for ensuring accurate current measurements. In our setup, an **isolated grounding scheme** was adopted, where the ground of the source measurement unit (SMU) was fully isolated from the ground of the acoustic-driven current source.

To verify the effectiveness of this configuration and rule out any influence from reverse currents, we conducted the following tests:

1. Connection Method

All current measurements were performed using a four-wire Kelvin connection with a Keithley 2450 SMU to ensure high precision.

2. Instrument Noise Test

The background current of the SMU under open-circuit conditions was measured to be below 5 pA, corresponding to the instrument's noise floor (Fig. 9).

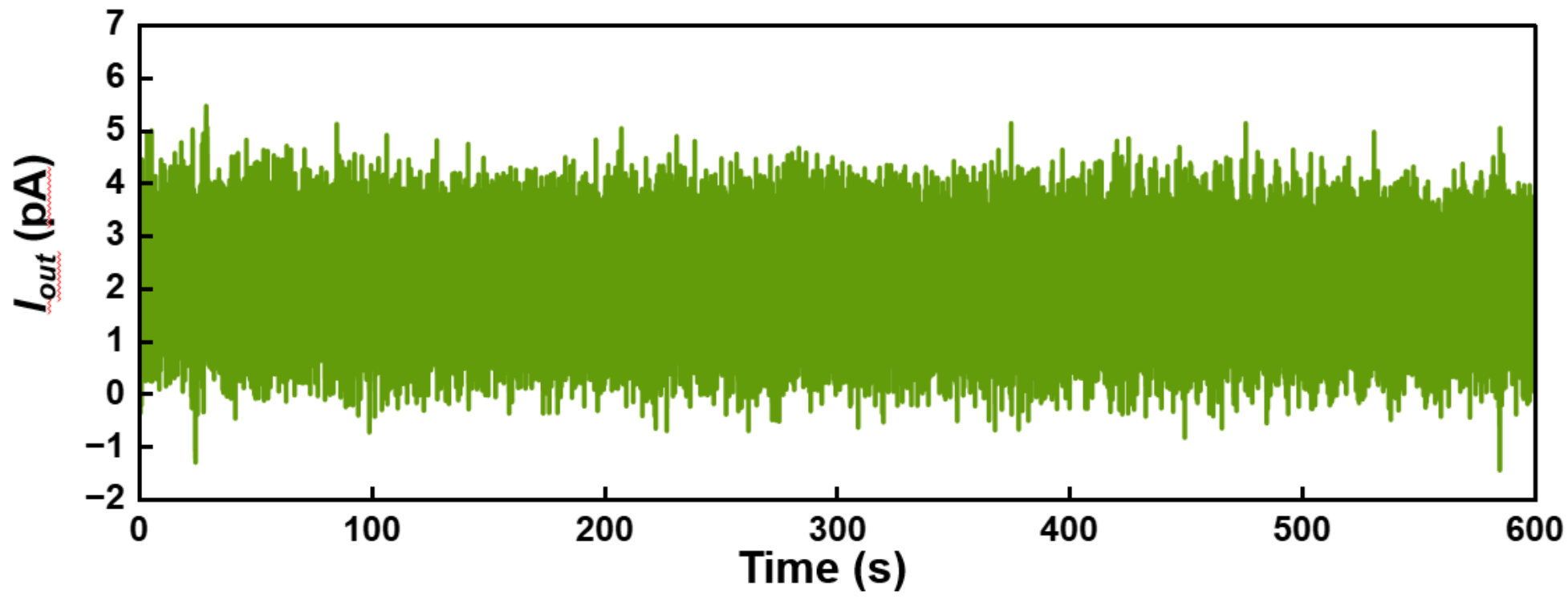

Fig. 9 Background current of the SMU under open-circuit conditions.

3. Reverse Current Validation

We employed a second Keithley 2450 SMU to assess whether ground potential differences could induce reverse currents. The procedure consisted of two steps:
First, we measured the current between the ground terminal of the SMU in the acoustic-driven current source measurement system (G_{SMU}) and the common ground.
Then, G_{SMU} was connected to the ground of the acoustic-driven current source (G_{ADCS}), and the current was measured again.

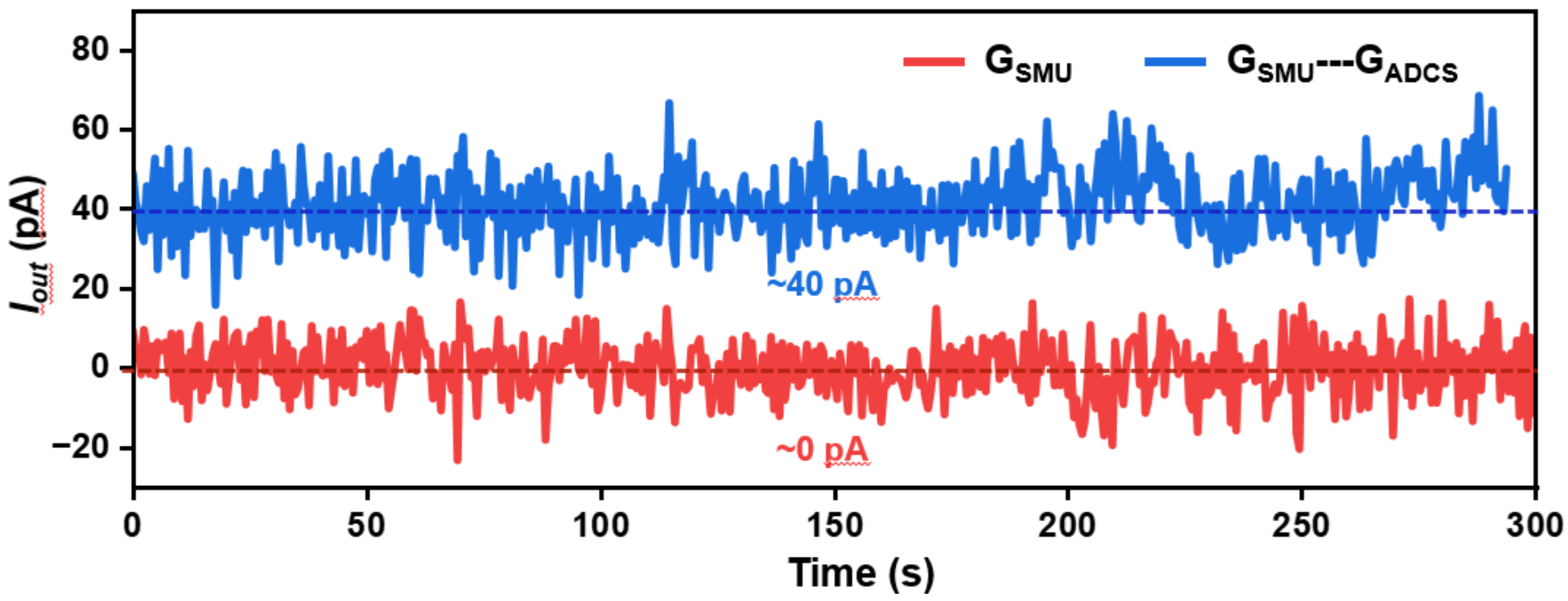

Fig. 10 Reverse current induced by ground potential difference.

As shown in Fig. 10, the current between G_{SMU} and the common ground was negligible. However, when G_{SMU} was connected to G_{ADCS}, a reverse current of approximately 40 pA was detected, caused by a ground potential difference. This reverse current accounts for only ~4% of the minimum output current of the acoustic-driven current source (1.22 nA), and thus has a negligible effect on measurement accuracy.

These validations demonstrate that the isolated grounding configuration is effective and does not introduce measurable error. Consequently, the experimental results obtained from the acoustic-driven current source are reliable and unaffected by grounding-related artifacts.

Clarification of the grounding configuration has been added to the revised manuscript, and **Supplementary Note XIII** provides detailed validation experiments supporting its effectiveness.

Revised:

1. "To ensure measurement accuracy, an isolated grounding scheme was adopted, in which the ground of the source measurement unit was electrically isolated from that of the acoustic-driven current source, thereby effectively eliminating ground loops and ensuring signal integrity by suppressing errors caused by ground potential differences (see Supplementary Note XIII for validation of this configuration and its negligible impact on measurement accuracy)." (Page 22)

Comment

7. The XRD measurements appear to have been taken on a separate InSb film. Please clarify whether these measurements were taken on the multilayer structure on the actual

LN substrate.

Response:

We thank the reviewer for the insightful comment regarding the XRD measurements. We confirm that the XRD data presented in this study were acquired from the $SiO_x/InSb/In_{0.6}Al_{0.4}Sb$ multilayer structure deposited on the $LiNbO_3$ substrate used in our device fabrication.

To accurately characterize the InSb layer within the multilayer structure, we employed grazing incidence X-ray diffraction (GIXRD). By carefully adjusting the incident angle, the X-ray penetration depth was controlled, effectively suppressing diffraction contributions from the underlying $In_{0.6}Al_{0.4}Sb$ layer and the $LiNbO_3$ substrate. This ensured that the diffraction patterns predominantly reflect the crystal structure of the InSb layer. No peaks corresponding to the SiO_x layer were observed, suggesting that it is amorphous or lacks long-range order.

These clarifications have been added to the revised manuscript. We also confirm that the XRD pattern shown corresponds exclusively to the multilayer structure and not to a separately grown InSb film.

Revised:

1. "**Fig. 2 Structural and electrical characterization of $SiO_x/InSb/In_{0.6}Al_{0.4}Sb$ heterostructure films**. **a** GIXRD patterns of the InSb layer, and **b** mobility and sheet carrier concentration of $SiO_x/InSb$ and $SiO_x/InSb/In_{0.6}Al_{0.4}Sb$ films. **c** SEM image, and **d-h** EDS mapping images of the $SiO_x/InSb/In_{0.6}Al_{0.4}Sb$ heterostructure films on a $LiNbO_3$ substrate." (Page 6)

Comment

8. Deposition of films thicker than 250 nm significantly affects the properties of the piezoelectric substrate, including SAW velocity, reflection, and electromechanical coefficients (K^2). It is crucial to explain how these effects were controlled during the experiment.

Response:

We would like to thank the reviewer for the valuable and constructive comment. The total thickness of the acoustoelectric (AE) film used in the experiments was 500 nm,

with the thickness of each layer being: SiO_x (100 nm)/InSb (250 nm)/InAlSb (150 nm). To characterize the effects of film thickness on the Rayleigh wave velocity V_p, electromechanical coupling coefficient K^2, and reflection properties, we focused on the active layer InSb, which plays a dominant role in the AE film. A periodic unit finite element model of the AE film region was established (Fig. 11a) to analyze the V_p and K^2 of InSb with different thicknesses (ranging from 50 nm to 450 nm), where the thicknesses of the SiO_x and InAlSb films were fixed at 100 nm and 150 nm, respectively. In addition, by simulating the cross-sectional model of the acoustic-driven current source along the acoustic wave propagation direction (Fig. 11c), the S-parameters of the device were obtained, thereby enabling the calculation of the variations between the maximum return loss RL_{max}, minimum insertion loss IL_{min}, and the thickness of the InSb film.

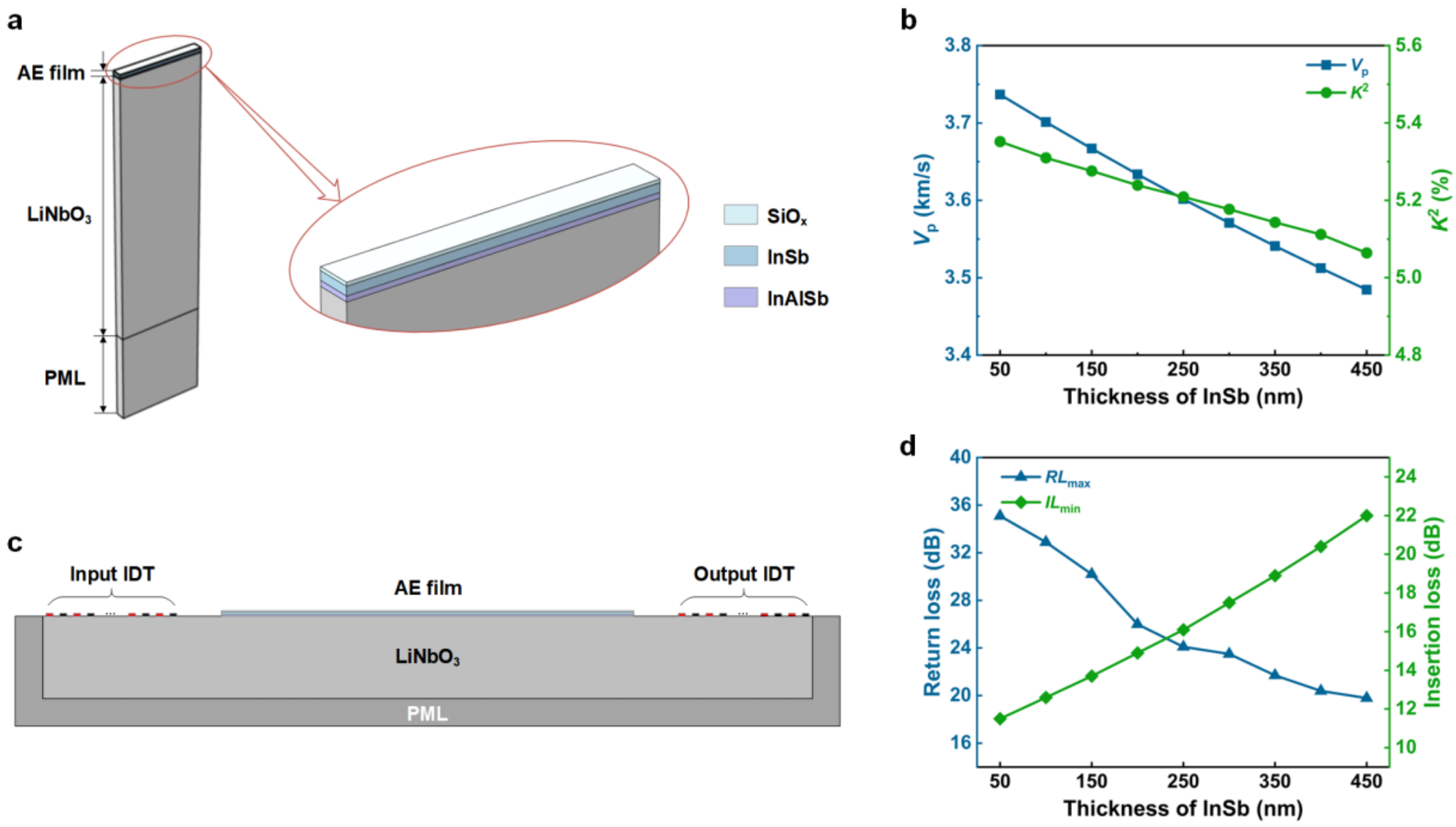

Fig. 11 a Periodic unit finite element model of the AE film region. b V_p and K^2 as functions of InSb thickness. c Schematic diagram of the cross-section of the acoustic-driven current source along the acoustic wave propagation direction. d RL_{max} and IL_{min} as functions of InSb thickness.

Fig. 11b and 1d show the variations between V_p, K^2, RL_{max}, IL_{min} and the thickness of InSb, where the calculation formulas for V_p and K^2 are as follows:

$$V_p = \frac{V_f + V_m}{2} \tag{1}$$

$$K^2 = \frac{V_f^2 - V_m^2}{V_f^2} \tag{2}$$

As the thickness of InSb increases from 50 nm to 450 nm, both V_p and K^2 show a decreasing trend (Fig. 11b), with V_p varying from 3737 m/s to 3484 m/s and K^2 ranging from 5.35% to 5.06%. RL_{max} and IL_{min} exhibit opposite variations (Fig. 11d): the former drops with increasing InSb film thickness (from 35.1 dB to 19.8 dB), while the latter grows continuously (from 11.5 dB to 22 dB).

From the perspective of physical mechanism, the Rayleigh wave velocity fundamentally reflects the combined effect of the effective elastic modulus E_{eff} and effective density ρ_{eff} of the propagation medium, and V_p is proportional to $\sqrt{E_{eff}/\rho_{eff}}$. As the thickness of InSb increases, its weight in confining the energy of Rayleigh waves gradually increases. Since InSb possesses a lower elastic modulus than the other layers ($LiNbO_3$ substrate, InAlSb and SiO_x) and a higher density, V_p decreases continuously. Simultaneously, thicker InSb allows more acoustic energy to transmit from the substrate into the InSb layer, and part of the acoustic energy is absorbed or converted into electrical energy in InSb. This reduces the effective acoustic energy density participating in piezoelectric coupling within the substrate, ultimately leading to a continuous decrease in K^2.

The decrease in RL_{max} stems from the dual negative effects of InSb thickness on electrical impedance and acoustic reflection: on one hand, the equivalent input resistance of the InSb layer decreases with increasing thickness, exacerbating impedance mismatch; on the other hand, as the thickness of InSb increases, constructive superposition of acoustic reflection echoes at multilayer interfaces becomes more likely, enhancing the total reflected acoustic energy. The increase in IL_{min} results from the dual effects of increased energy dissipation and reflection power encroaching on transmission power: thicker InSb increases the number of effective carriers, converting more acoustic energy into the kinetic energy of carriers, and meanwhile, the acoustic energy loss caused by the accumulation of lattice defects also intensifies; in addition, the increase in reflected power reduces the effective transmitted acoustic energy, further decreasing the power at the output end of the delay line.

In the AE film described in this paper, the thickness of InSb is set to 250 nm, at which point the V_p and K^2 of the AE film region are 3602 m/s and 5.21%, respectively. Although thinner InSb exhibits larger V_p and K^2, the insufficient ability to confine acoustic energy limits the intensity of AE conversion. In contrast, excessively thick InSb exacerbates acoustic wave reflection and energy dissipation. Based on these comprehensive considerations, it is finally determined that 250 nm is a relatively appropriate value.

Reviewer: 6

Comment

1. I'm not sure I agree that nano-amp level current sources cannot be generated using standard CMOS transistors techniques. This is done all the time in nanoWatt level integrated circuits highly published in the circuits research community. While such current sources may not be available commercially, I suspect this is due to the lack of a market for current sources with such small output levels as opposed to the inability of CMOS transistors to produce nA level current sources.

Response:

We thank the reviewer for the comment. We acknowledge that CMOS technology is capable of generating nanoampere-level current sources[1-3]. The proposed acoustic-driven current source, however, operates on a fundamentally different principle. It employs the AE effect to convert RF signals directly into a DC output.

Moreover, the use of a SAW device allows the current source to operate without wired power connections, enabling non-contact energy delivery to low-power devices. This feature opens new possibilities for powering compact or distributed systems, such as wireless sensors or implantable devices, where ultra-low-power and autonomous operation is essential.

In summary, while CMOS circuits can produce nanoampere currents, the acoustic-driven current source offers unique advantages in terms of wireless operation, miniaturization, and energy harvesting, which are not achievable with standard transistor-based approaches.

References:

1. Ballo, A., Grasso, A. D. & Privitera, M. A 6.3-ppm/°C, 100-nA Current Reference With Active Trimming in 28-nm Bulk CMOS Technology. *IEEE Access* **10**, 108342-108353 (2022).
2. Yan, Y., Almeida, G. F. F. D., Yang, Z. & Dong, T. in *2022 International Conference on Electronics, Information, and Communication (ICEIC)*. 1-5.
3. Ding, L., Wang, Y., Bao, Z., Liao, H. & Jin, X. A nano-ampere current reference circuit in a 0.5 μm CDMOS technology. *Microelectronics Journal* **90**, 336-341 (2019).

Revised:

1. "Although CMOS technology can generate nanoampere-level currents[9-11], most com-mercially available current source chips target higher current ranges, typically from mi-croamperes to milliamperes. For example, Texas Instruments offers adjustable current sources from 1 μA to 10 mA at millimeter-scale device sizes, which makes stable, contin-uous operation at the nanoampere scale a significant challenge." (Page 2)

Comment

2. What is the sheet resistance or resistivity of the InAlSb layer? If this layer is conductive, what is the impact on the AE effect of placing it between the InSb and the $LiNbO_3$?

Response:

We sincerely thank the reviewer for this insightful question. The following explanation clarifies the resistive properties of the InAlSb buffer layer and their influence on the AE effect.

The $In_{0.6}Al_{0.4}Sb$ buffer layer exhibits a resistivity of approximately 2000 Ω·cm, which is about four orders of magnitude higher than that of the SiO_x/InSb/$In_{0.6}Al_{0.4}Sb$ multilayer film, whose resistivity is below 1 Ω·cm. This pronounced difference is critical, as the high-resistivity buffer layer effectively suppresses the attenuation of SAWs, thereby enabling strong AE interactions between the charge carriers in the InSb active layer and the electric fields associated with the SAWs.

By contrast, if the buffer layer were conductive, it would screen these electric fields, reduce the coupling between electrons and phonons, and consequently diminish the AE effect.

Comment

3. What is the cause of the passband ripples in the AE current in figures 2 b and c? I would have thought this was triple transit in (a) without the InSb but shouldn't the AE effect reduce the triple transit since it damps the SAW wave in this case?

Response:

We appreciate the reviewer's insightful comment. The passband ripples observed in the AE current in manuscript Figs. 2b and 2c primarily originate from the triple transit. While the InSb film reduces triple transit, residual reflections remain, and passband ripples therefore persist in the AE current.

Specifically, these ripples from interference between the primary acoustic wave and its multiple reflections. Since the AE current amplitude is positively correlated with acoustic wave intensity, the ripples in S_{21} near the center frequency are directly manifested in the AE current.

Moreover, Supplementary Fig. 3 shows that passband ripples in S_{21} exist both before and after the deposition of the InSb film, consistent with our COM model simulations. The InSb film causes attenuation of the acoustic waves, leading to an increase in insertion loss from 8.61 dB to 16.74 dB. This attenuation reduces the reflected wave energy, partially suppressing the triple transit and thus decreasing the passband ripple amplitude. However, since reflection is not fully eliminated, the S_{21} still exhibits passband ripples, which are accordingly observed in the AE current curves in manuscript Figs. 2b and 2c.

Comment

4. Can the authors explicitly state that the output is a DC current from the input RF signal. The reviewer is afraid this point is not made explicitly clear and the RF plots in Fig. 2 may confuse some readers unfamiliar with the AE effect.

Response:

We thank the reviewer for the valuable comment. We clarify that the measured AE current in manuscript Fig. 2 is a direct current generated from the input RF signal via the acoustoelectric effect in the semiconductor film. To avoid confusion, the manuscript and the caption of manuscript Fig. 2 have been revised to explicitly state that the AE current is direct current.

Revised:

1. "The AE effect converts the applied RF signal into a DC current in the semiconductor film. Current characteristics of the acoustic-driven current source are shown in Fig. 3." (Page 8)
2. "The AE current, which is a DC current output generated from the input RF signal

via the AE effect, was then measured without any applied DC bias." (Page 8)

Comment

5. The current source has a very low RC filter characteristic that appears at 100 kHz. What is the source of the large capacitance causing such a low corner frequency?

Response:

We thank the reviewer for raising this insightful question. In our work, the acoustic-driven current source can be approximately represented by a parallel RC equivalent circuit, consistent with previous reports on acoustoelectric devices. The corner frequency of such a network is given by:

$$f_c = \frac{1}{2\pi RC} \tag{3}$$

Using the measured resistance, we can estimate the corresponding capacitance from the observed corner frequency. From our impedance measurements, the extracted resistance is about 10.5 kΩ. If the observed RC corner is around 100 kHz, the corresponding capacitance is estimated to be approximately 150 pF. This value is noticeably higher than the capacitance expected purely from the intrinsic properties of the piezoelectric substrate and the IDT structure, suggesting that additional contributions from parasitic elements (e.g., probing pads, cables, connectors, and instrument input) may be involved.

Overall, this analysis indicates that the apparent low RC corner frequency originates from both the intrinsic device capacitance and extrinsic parasitic contributions. Further optimization of the IDT design and measurement setup may help reduce these effects in future work.

References:

1. Ghosh, S., Hollis, M. A. & Molnar, R. J. Acoustoelectric amplification of Rayleigh waves in low sheet density AlGaN/GaN heterostructures on sapphire. *Applied Physics Letters* **114**, 063502 (2019).

Comment:

6. At 10 mW RF input power, a current of approximately 2 microA is produced with a

source impedance of approximately 10.5 kOhms. Under a maximum power transfer condition to the load, the amount of delivered power will be approximately 10 nW, making the efficiency 0.0001%. Can the authors comment on the utility of such an inefficient current source?

Response:

We thank the reviewer for this important and insightful comment. We acknowledge that the present design of the acoustic-driven current source exhibits relatively low energy conversion efficiency. As illustrated in Fig. 12, this limitation arises from its intrinsic working principle, which requires a multi-stage energy conversion process—from electrical to acoustic, and subsequently back to electrical—unlike conventional transistor-based current sources.

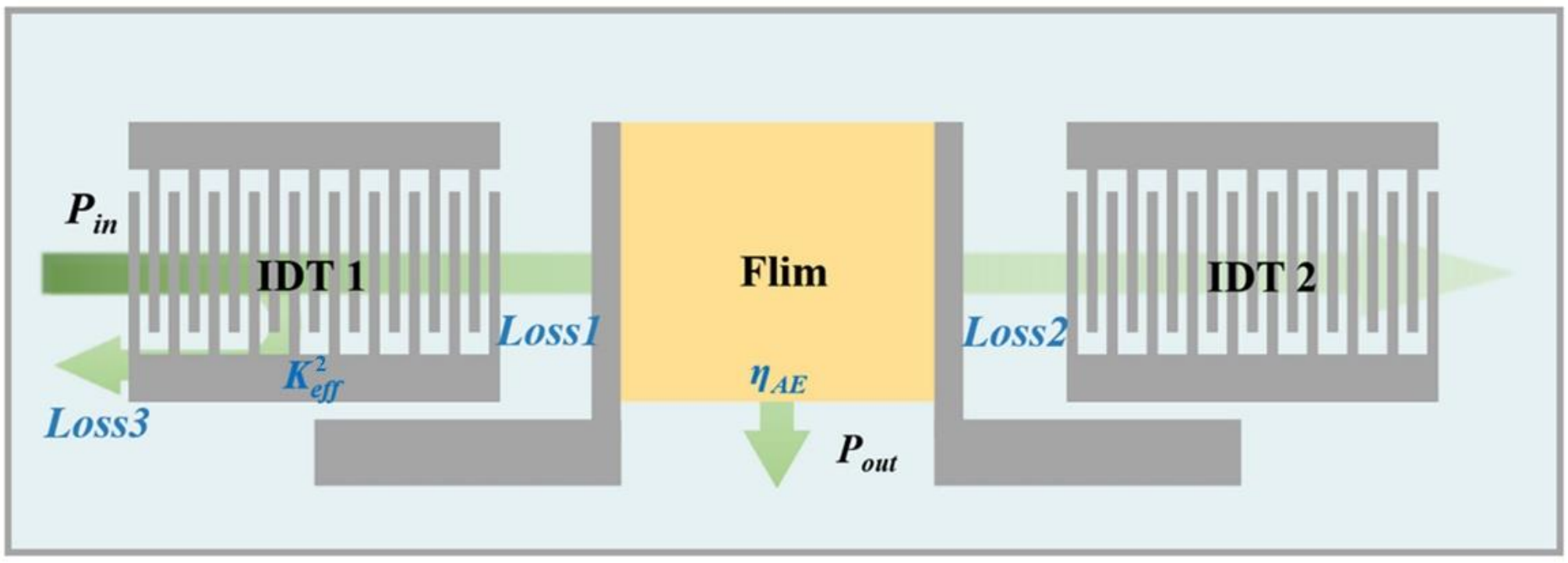

$$P_{out} \approx P_{in} \cdot \eta_{K^2_{eff}} \cdot \eta_{Loss} \cdot \eta_{AE} \cdot \eta_{other}$$

Fig. 12 Schematic illustration of the energy conversion process in the acoustic-driven current source.

The overall efficiency is determined by three key factors:

1. Electromechanical Coupling Coefficient (K^2_{eff})

The electromechanical coupling coefficient K^2_{eff} governs the conversion efficiency between RF electrical signals and SAWs and directly determines the magnitude of the acoustoelectric effect. The 128°YX $LiNbO_3$ substrate used in our device has a relatively high K^2_{eff} (~5%), superior to other common piezoelectric materials such as quartz (~2%) and $LiTaO_3$ (~4%).

2. Acoustic Wave Propagation Efficiency (η_{Loss})

Unavoidable losses occur during SAW propagation in the substrate, arising from

surface roughness, internal inhomogeneities, lattice defects, lattice thermal vibrations, and air loading. These losses are intrinsic to the material and increase with frequency. To quantify their impact, we measured the insertion loss before and after film deposition: −8.61 dB without the film (13.78% of power reaching IDT2) and −16.74 dB with the film (only 2.21% reaching IDT2). While part of the additional loss is attributable to acoustoelectric conversion in the film, the majority originates from propagation loss within the multilayer structure.

3. Acoustoelectric Conversion Efficiency (η_{AE})

The attenuation of SAW energy, Δα, induced by the acoustoelectric effect is expressed as[1]:

$$\frac{\Delta\alpha}{k} = \frac{K_{eff}^2}{2} \frac{\sigma_S/\sigma_M}{1+(\sigma_S/\sigma_M)^2} \tag{4}$$

where k is the wavenumber; K_{eff}^2 is the electromechanical coupling coefficient; σ_S is the sheet conductivity of the acoustoelectric film; and σ_M is the characteristic sheet conductivity of the piezoelectric substrate. This formula indicates that the acousto-electric conversion efficiency, η_{AE}, depends not only on the conductivity of the film but also strongly on the electromechanical coupling coefficient, K_{eff}^2. Accordingly, we optimized the film conductivity and selected substrates with higher K_{eff}^2 to enhance the overall efficiency.

Despite its currently low energy conversion efficiency, the acoustic-driven current source offers several unique advantages that conventional transistor-based sources cannot provide. Its millimeter-scale footprint enables significant miniaturization, and the device can operate under extreme environmental conditions. Moreover, it can be wirelessly powered via RF signal excitation, eliminating the need for wired connections. These features render it particularly promising for emerging applications such as implantable medical devices and miniature RF systems. For example, an implanted piezoelectric chip could harvest external RF signals to generate a stable current for biosignal detection, replacing invasive catheter-based wiring. Similarly, the device could be deployed wirelessly on rotating or enclosed components, overcoming the limitations of conventional wired current sources.

Looking forward, we aim to enhance overall energy conversion efficiency through

multiple strategies. First, employing piezoelectric substrates with higher electromechanical coupling coefficients, such as 41°YX $LiNbO_3$ (K^2_{eff} ≈ 17%), can improve Acoustoelectric conversion efficiency. Second, the use of shear horizontal (SH) waves may reduce acoustic energy leakage. Third, advanced IDT designs, including single-phase unidirectional transducers (SPUDTs), can minimize bidirectional losses. Finally, further optimization of the acoustoelectric layer conductivity will facilitate more effective energy transfer. Collectively, these improvementsare expected to pave the way toward practical, efficient, and scalable acoustic-driven current sources.

References:

1. Hutson, A. R. & White, D. L. Elastic wave propagation in piezoelectric semiconductors. *Journal of Applied Physics* **33**, 40-47 (1962).

Comment

7. The output impedance of the current source of 10.5 kohms is quite low compared to transistor based cascode current sources. Can the authors comment on the errors induced by the low output resistance?

Response:

We thank the reviewer for raising this insightful question regarding the relatively low output impedance (~10.5 kΩ) of the proposed acoustoelectric current source. Overall, while this impedance is substantially lower than that of typical transistor-based current sources and may impose limitations in certain high-precision applications, its practical effect depends strongly on the load conditions. Moreover, optimizing film fabrication and device structure provides a promising route to enhance the output impedance.

A finite output impedance introduces deviation from ideal current source behavior, and its impact depends on the relative magnitude of the source and load resistances. For example, when the load resistance is comparable to the source resistance (e.g., 1 kΩ), the resulting error in delivered current is modest (<10%). In contrast, for much higher resistive loads (e.g., 100 kΩ), the output current would be more significantly affected. This indicates that the present device is better suited for loads with relatively low resistance.

The output resistance of an acoustoelectric current source is primarily determined by the electrical properties of the acoustoelectric multilayer film. According to the AE

current expression:

$$I_{AE}=\frac{\mu Pf}{v_S{}^2}K_{eff}^2\pi\frac{\sigma_S/\sigma_M}{1+(\sigma_S/\sigma_M)^2} \quad (5)$$

optimal energy transfer occurs when the sheet conductance σ_S approaches the characteristic conductance σ_M. In our device, the InSb-based multilayer film has $\sigma_S \approx 1.9\times10^{-4}$ S, which is higher than $\sigma_M \approx 2.04\times10^{-6}$ S. Since film resistance is inversely proportional to conductance, reducing σ_S via process optimization (e.g., controlling doping, film thickness, or interface quality) can increase the output impedance while maintaining sufficient AE current due to the high mobility of InSb.

In summary, although the current device exhibits an output impedance of ~10.5 kΩ, suitable for wireless low-power applications, future advances in film fabrication and device structure may further increase the impedance and extend its use to higher-precision measurement scenarios.

Comment

8. What is output voltage range over which the current source can operate?

Response:

We thank the reviewer for the valuable comment. To clarify the operational characteristics of the acoustic-driven current source, we measured its output voltage range using a source measurement unit (Keithley 2450). As shown in Fig. 13, the output voltage spans from 0 to approximately 335 mV. These measurements were performed under the same conditions as those used for acoustoelectric current measurements.

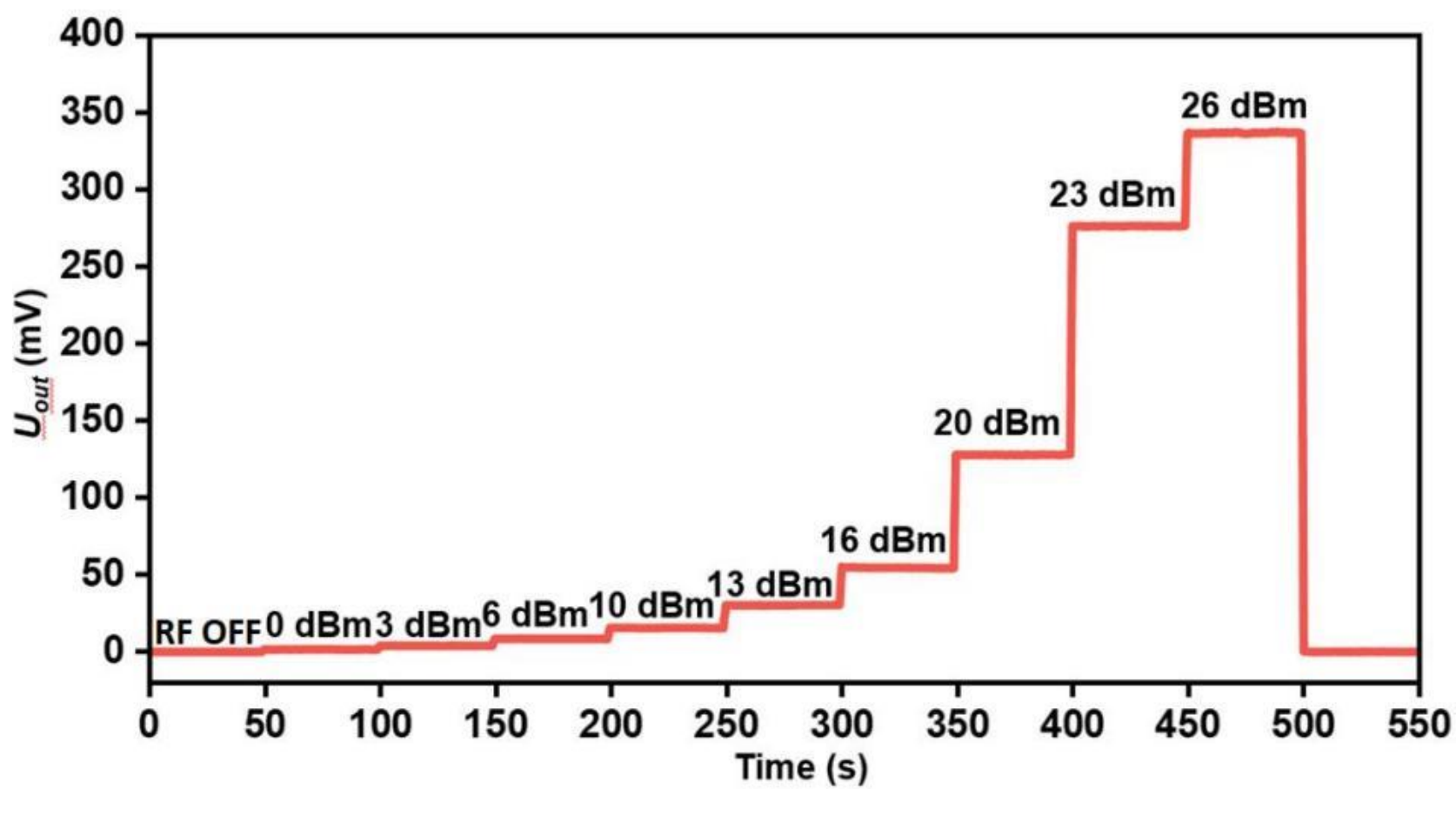

Fig. 13 Output voltage range measured during operation of the acoustic-driven current source.

The experimental details and corresponding data have been included in the revised **Supplementary Note IV.**

Comment

9. The authors state "The output impedance of the acoustic-driven current source is slightly lower than that of voltage-controlled current sources, which typically exhibit impedance in the mega-ohm range." Two orders of magnitude lower is not slightly lower. It is substantially lower.

Response:

We thank the reviewer for pointing out this inaccuracy. We agree that the phrase "slightly lower" could be misleading. A detailed discussion of the impact of output impedance on load-dependent performance is provided in our response to Comment 7. Regarding the manuscript, we have revised the original sentence for clarity and accuracy. This revision clarifies the statement while also highlighting the potential for future improvements.

Revised:

1. "Overall, while the output impedance of the acoustic-driven current source is lower than that of conventional voltage-controlled current sources, it remains sufficiently high for intended low-power wireless applications, particularly when the load resistance is much smaller than the source impedance (e.g., < 1 kΩ), ensuring that current errors due to loading remain below 10%." (Page 13)
2. "With further optimization of film fabrication and device structure, the output impedance may be further enhanced, potentially extending the applicability of the device to higher-precision measurement scenarios." (Page 13)

Reviewer: 3

Comment 1. Does and load resistor used in the AC measurement? If the load is high resistance, it could be possible that the output resistor-capacitor (RC) limits the observation of AC current component. Especially in the Fig. 5 in Author Response, there is a large DC bias current, indicating there might be a large capacitor holding the output current/voltage.

Response:

We fully agree with your comments. In the preliminary tests, a longer integration time was set on the source measure unit (Keithley, 2450) in order to acquire an extremely weak and stable nanoampere-level DC current. This, unfortunately, resulted in an extremely low actual sampling rate. This configuration effectively rendered the source measure unit equivalent to a strong low-pass filter at the hardware level: it performed long-term averaging of the high-frequency current variations, thereby smoothing out the AC components that should have been present. Consequently, only a large DC offset was observed in the previously obtained plots, without capturing AC signals. In addition, when the spectral analysis was previously performed using the built-in FFT function of the oscilloscope, the output impedance of the SAW device under test was mismatched severely with the 50 Ω input of the oscilloscope. As a result, the signal was significantly attenuated before reaching the sampling front end, and its level was fallen below the noise floor, leading to the absence of effective characteristic peaks in the FFT spectrum.

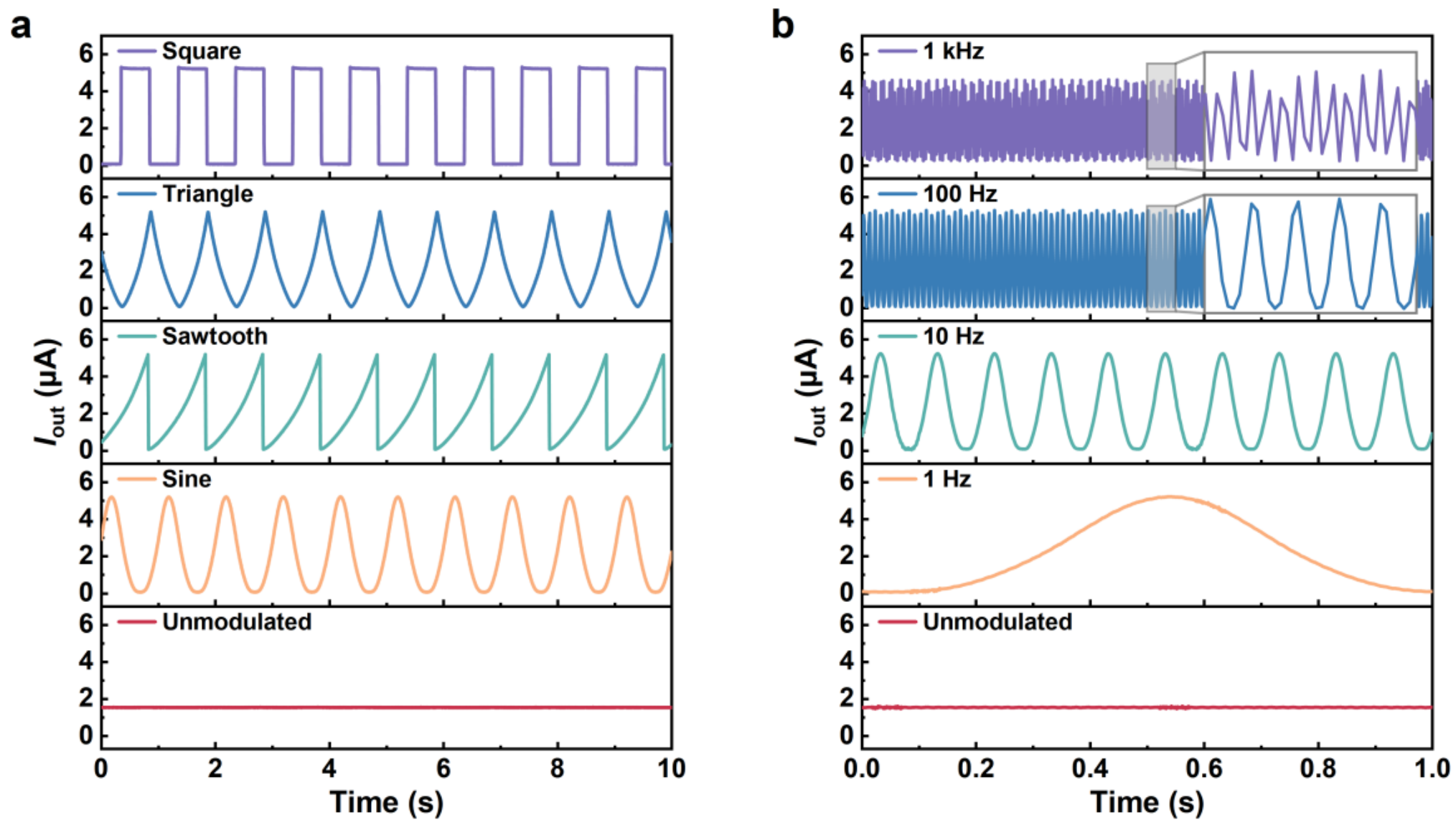

Fig. 1. **Modulation-dependent temporal characteristics of the output current.** **a** Periodic fluctuation of I_{out} over time under different amplitude modulation functions at a modulation frequency of 1 Hz. **b** Periodic fluctuation of I_{out} over time under different modulation frequencies with a sine-wave amplitude modulation function.

Following your good suggestion, we increased the sampling rate of the Keithley 2450 to its maximum possible (or hardware-supported) value (5000 samples/s). The retested results aligned well with expected physical behavior.

As shown in Fig. 1a, when the RF input was amplitude-modulated at 1 Hz (with modulation waveforms including sine, sawtooth, triangle, and square waves), we clearly observed periodic AC variations in the output currents I_{out} corresponding to the modulation signals.

As shown in Fig. 1b, when the modulation frequency was gradually increased to 1 kHz, limited by the theoretical maximum sampling rate of 5000 samples/s, the periodic fluctuation of the current was still be identified, but the recorded waveform was severely distorted. This clearly reveals that the high-frequency responses indeed exist but are constrained by the instrument's sampling limit.

Therefore, we can conclude that the major reason why the 1 kHz AC response was not observed previously is not because of the presence of a large parasitic capacitance inside the device, but is rather due to the time-averaging effect induced by the low-frequency sampling mode of the source measure unit.

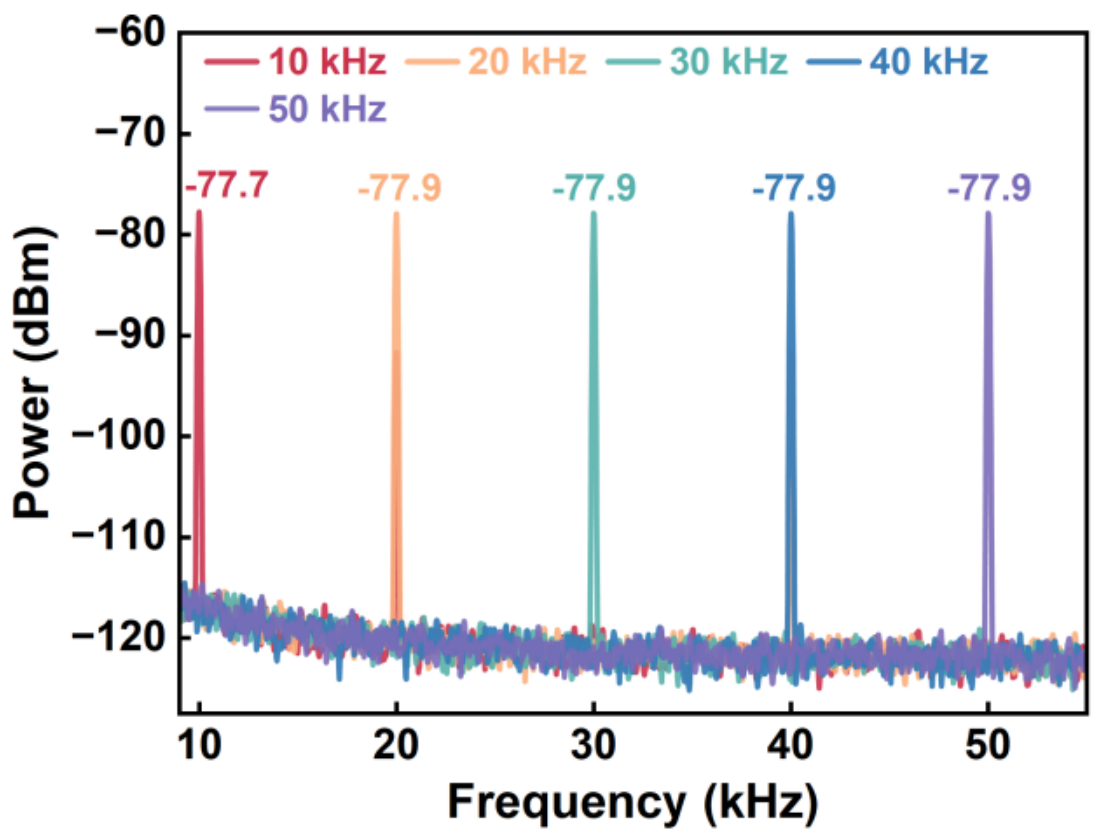

Fig. 2. Spectral response characteristics of I_{out} under different modulation frequencies (10 kHz, 20 kHz, 30 kHz, 40 kHz, and 50 kHz)

To further verify the AC response characteristics of the device in the high-frequency region and characterize its 3 dB bandwidth, the device's output was connected to a spectrum analyzer (RIGOL, RSA3030E-TG) via a 10 dB attenuator. The obtained data are shown in Fig. 2. Considering that the lower frequency limit of the spectrum analyzer was 9 kHz and the maximum amplitude modulation frequency of the RF signal generator (SRS, SG394) was 50 kHz, the spectral observation range in these tests was set from 9 kHz to 55 kHz. A sine wave was used as the modulation function, with the modulation frequency set sequentially to 10 kHz, 20 kHz, 30 kHz, 40 kHz, and 50 kHz. The obtained response curves exhibited the corresponding peaks at each frequency. Within the range of 10 kHz to 50 kHz, the fluctuation in the peak power was not larger than 0.2 dBm, indicating that

the device maintained a good AC response capability throughout the tests, and its 3 dB bandwidth was significantly larger than 50 kHz.

The above results are added in the **Supplementary Information (Supplementary Note XIV)**.

Comment 2. If the current response is so slow, maybe a current rise/fall time when turning on/off SAW could be measured?

Response:

We highly appreciate your good suggestion. As discussed in our response to Comment 1, the apparent "slow" current response observed previously was solely an artifact caused by the integration time limits of the source measure unit (i.e., the Keithley 2450). Because the acoustoelectric effect is fundamentally governed by the phonon-electron momentum transfer, its theoretical dynamic response time is, in principle, exceptionally fast, typically on the order of microseconds or less. The intrinsic transient response of the device is primarily determined by the acoustoelectric build-up time (τ), which is corresponding to the time required for the SAWs to traverse the active interaction region. This theoretical time can be estimated by the following equation:

$$\tau = \frac{L}{v_S} \quad (1)$$

where L=1200 μm is the effective interaction length, and v_S is the acoustic velocity on the 128°YX $LiNbO_3$ substrate, which is 3992 m/s. Accordingly, the theoretical build-up time is estimated to be 0.30 μs.

The standard source measure units cannot resolve such high-speed transients, and the standard high-frequency oscilloscopes is quite difficult to directly resolve minute nA-to-μA level current steps without involving in severe wideband noise. To solve this issue, we have now upgraded our transient measurement setup. We employed a high-frequency transimpedance amplifier (LTC6268) coupled with a high-speed, broadband oscilloscope (Siglent, SDS6104 H10 Pro). The RF signal generator was configured in a pulsed excitation mode with a pulse period of 500 μs, a pulse width of 5 μs, and an output power of 14 dBm. The gain of the high-frequency transimpedance amplifier was set to be 10^4 V/A, effectively converting μA-level current into a measurable mV-level voltage for the oscilloscope.

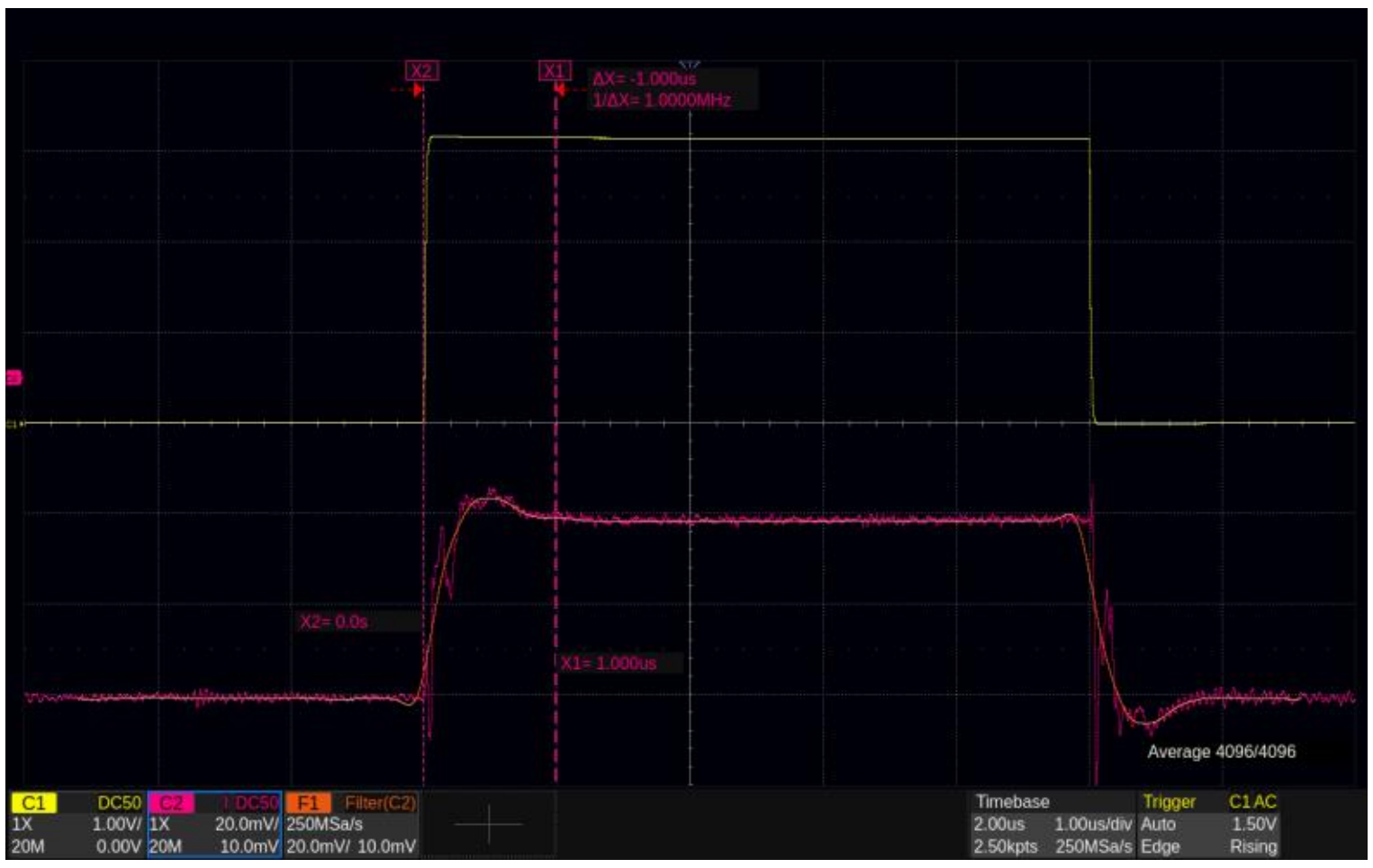

Fig. 3 Transient response measurement. The yellow curve (C1) represents the RF pulse excitation, the pink curve (C2) denotes the output response, and the orange curve (F1) shows the filtered signal envelope.

The typical time-domain waveform is presented in Fig. 3. The measured intrinsic response time is less than 1 μs, defined as the interval from the onset of the RF pulse (C1) to the point where the response signal reaches a steady-state flat top (C2). It is important to note that this measured macroscopic time (<1 μs) naturally exceeds the theoretical acoustoelectric build-up time (0.30 μs). This value is physically consistent, as the measured total response time represents a convolution of the following three sequential delays. (1) The acoustic delay time required for the SAW to travel from the input IDT to the edge of the active film; (2) The aforementioned intrinsic acoustoelectric build-up time required for the SAW to fully cover the 1200 active region to reach the maximum steady-state current; (3) The minute parasitic charging delays within the external transimpedance amplifier measurement circuit. Considering these cumulative factors, the measured macroscopic transient time of sub-1 μs correlates well with our sub-microsecond theoretical estimation, strongly confirming that the acoustic transit time is indeed the core physical factor dictating the device's dynamic speed.

It is worth noting that the initial ringing observed on the rising and falling edges of the raw signal (C2) is a known artifact which is originated from the inherent underdamped response and parasitic LC resonance of the high-frequency transimpedance amplifier circuit when processing ultra-fast, weak current edges. This oscillation rapidly attenuates within 1 μs. By applying a 2 MHz digital low-pass filter to the raw data, the resulting envelope (F1) closely resembles a smooth and ideal step response.

Through these rigorous transient measurements and theoretical validations, we have unequivocally verified the microsecond-level intrinsic response time of the device, thereby confirming its capability for extremely rapid AC tracking. We thanks for these good comments, which guided us

to fully uncover and prove the high-speed dynamic nature of our acoustoelectric current source. The details of these transient measurements have now been incorporated into the **Supplementary Information (Supplementary Note XIII)**.

Comment 3. In Fig. 4c of previous paper [Hackett, L., Miller, M., Brimigion, F. et al. Towards single-chip radiofrequency signal processing via acoustoelectric electron–phonon interactions. Nat Commun 12, 2769 (2021). https://doi.org/10.1038/s41467-021-22935-1], the acousto-electric interaction shows μs level response time. Are there any significant difference in devices that lead to no AC response in this work? Do authors have any insights / comments explaining this no AC response?

Response:

We thank you for pointing out the outstanding work by Hackett et al. These are profound and highly insightful questions.

We would like to clarify that there are no fundamental device differences which would intrinsically lead to the absence of high-speed AC response in our work. At the foundational physical level, where the charge carriers are directed by the piezoelectric field of propagating SAWs, our device operates based on the same electron-phonon momentum transfer mechanism as the devices reported in Hackett's work. Theoretically, both these devices should possess extremely rapid, microsecond-level acoustoelectric dynamic response times.

As proven by our newly performed transient measurements as provided in our response to Comment 2, we successfully captured a sub-1 μs response using a high-frequency transimpedance amplifier coupled with a high-speed, broadband oscilloscope, and proved that our device inherently possesses excellent high-speed AC tracking capabilities.

Our explanation for the apparent discrepancy in macroscopic behaviors reported in these two studies (prior to our new transient measurements shown above) is that the fundamentally different measurement setups, designed for distinct application purposes, led to differences in the observed response characteristics. The details are discussed below.

In Hackett's work: Their primary research objective was to achieve single-chip radiofrequency signal processing, including non-reciprocal RF amplification and isolation. Consequently, they employed ultra-high-speed microwave testing platforms such as vector network analyzers and high-bandwidth RF probe stations. These RF instruments possess gigahertz bandwidths, allowing them to resolve and capture the nanosecond-to-microsecond acoustoelectric interactions.

In our present work: Our core objective was to provide an extremely stable and continuous DC

microcurrent source for miniaturized, low-power bioelectronic applications, such as *in vivo* wound healing. To precisely calibrate and characterize the absolute accuracy and long-term stability of these minute nanoampere-to-microampere DC outputs over hours of operation, we deliberately selected a highly sensitive source (i.e., Keithley 2450). As discussed in the Comment 1, achieving such extreme DC precision inherently requires long hardware integration times to suppress noise. This integration mechanism inevitably acted as a powerful low-pass filter, inadvertently masking the device’s inherent high-speed μs-level AC dynamic response and presenting it as a "pure, slow DC" signal in our original manuscript.

We are grateful for these raised questions, and have been guided to fully uncover the true high-speed physical nature of our device, thereby clarifying a critical detail that could have otherwise misled future readers.

As detailed in our responses to Comments 1 and 2, we have thoroughly addressed the sampling limitations of the source measure unit and provided new transient response and high-speed AC modulation data in the **Supplementary Notes XIII and XIV**.

Reviewer: 6

Comment 1. This concept is not new and the reported work is not an advancement over previously published work. A superior performance device using LN (piezo) on Si (semiconductor) was reported by Mansoorzare in DOI: 10.1109/MEMS58180.2024.10439505 where an RF input power of 10 mW (at 661 MHz) produced a DC output current of 10 μA via the acoustoelectric effect compared to the approximately 2 μA current reported here for the same RF input power.

Response:

We thank the reviewer for bringing this excellent and highly relevant work by Mansoorzare et al.. We apologize for missing this very recent progress in our previous literature review, and we have now cited and briefly discussed this paper in the revised manuscript.

We fully acknowledge that, as you correctly pointed out, the device reported by Mansoorzare et al. achieved a superior DC output of approximately 10 μA at a 10 mW RF input. However, we would like to clarify that the underlying acoustic wave modes, fabrication paradigms, and semiconductor material systems in their work are fundamentally different from ours, representing two distinct approaches with different trade-offs and scientific objectives.

The high yield reported in Mansoorzare's work was achieved by utilizing a suspended micromachined lithium niobate on silicon (LN-on-Si) waveguide. Such the suspended membrane architectures primarily excite Lamb waves (specifically the S_0 mode). The physical advantage of such suspended Lamb wave devices is their ability to strictly confine acoustic energy within the ultrathin membrane. It effectively prevents radiation leakage into the bulk substrate, thereby maximizing the effective electromechanical coupling coefficient (K_{eff}^2). However, this high performance is inherently accompanied by highly complex MEMS fabrication processes, which require precise wafer bonding, thinning, and deep reactive ion etching for cavity release, thereby significantly increasing manufacturing costs and limiting their direct monolithic integration, particularly on flexible or large-area platforms.

In contrast, our work is focused on the monolithic integration of an InSb semiconductor heterostructure directly deposited onto a bulk piezoelectric substrate via physical vapor deposition, exciting standard Rayleigh SAWs. As analyzed in our manuscript based on Eq. (1), the generated AE current is theoretically governed by three key factors, i.e., (1) the piezoelectric substrate (proportional to K_{eff}^2 and inversely proportional to v_{S}; (2) the IDT structure design; and, most crucially, (3) the material properties of the AE film. To maximize the energy transfer from acoustic waves to semiconducting carriers, it is physically vital to maximize the carrier mobility μ while ensuring that the film's sheet conductivity σ_{S} strictly matches the characteristic conductivity σ_{M} of the piezoelectric substrate.

Mansoorzare et al. utilized silicon platform, where conductivity can be easily tuned through standard doping on transferred crystalline wafers. However, we addressed the challenging by monolithically growing InSb directly on a highly lattice-mismatched $LiNbO_3$ substrate. We selected the InSb to take advantage of its theoretically superior electron mobility required for the acoustoelectric mechanism. The Rayleigh SAW platform on a bulk substrate experiences certain acoustic energy dissipation, which lowers the effective electromechanical coupling coefficient and reduces the current yield to about 2 μA for a single delay line. Nevertheless, this approach provides substantial benefits, including simpler fabrication, lower cost, and improved monolithic scalability.

To compensate for the natural efficiency limits of the bulk Rayleigh SAW platform, our work explores system-level optimizations (SPUDT and multi-IDT arrays) and, most importantly, advances the application to an in vivo animal model, validating that this μA-level current is already highly effective for applications in the bio-modulation. We have added a concise discussion in the manuscript **(Introduction section, Page 3)** to acknowledge these different structural approaches.

Revised:

1. "Recent advancements in AE devices have explored suspended thin-film architectures, such as lithium niobate-on-silicon waveguides supporting Lamb waves, which achieve impressive DC yields by strictly confining acoustic energy within the membrane[20]. However, fabrication of such suspended structures typically involves complex wafer bonding and cavity release processes, significantly increasing manufacturing costs and integration complexity. Therefore, developing AE current sources based on simpler and monolithically grown heterostructures on bulk piezoelectric substrates remains highly desirable, despite the inherent challenges of material integration." (Page 3)

Comment 2. The current source still has an extremely poor RF-to-DC power conversion efficiency of 0.0001%. Despite claims to the contrary in the paper, this will result in significantly degraded performance when compared to traditional approaches of forming wireless current sources such as the use of rectifiers combined with transistor circuit based current sources. More on this below.

Response:

We greatly appreciate your rigorous critique regarding the RF-to-DC power conversion efficiency of our device. As you accurately pointed out, the conversion efficiency of the acoustoelectric effect in our current device configuration is orders of magnitude lower than that of conventional diode-based rectifiers. We completely agree that in any traditional application aiming for highly efficient, point-to-point wireless power transfer, this inherently low efficiency would inevitably result in significantly degraded system performance compared to those standard transistor-based circuits. We apologize for our previous overstatements that may have misinterpreted this fundamental physical

limitation.

Following your stringent comments and the Editor's advice to "contain a clear and transparent discussion of the limitations," we have detailed the key limitations and future perspectives into the revised manuscript, in the **Discussion section (Page 21)**. We honestly acknowledge this efficiency gap to ensure readers not misled regarding the device's capability in general power harvesting scenarios.

However, we would also like to clarify how this efficiency deficit can be partially mitigated in the specific context of our application. Although the efficiency of a single and isolated device is substantially low in practical wireless environments, this RF energy is broadcasted spatially. A single RF source can simultaneously excite multiple acoustoelectric devices distributed over an area, which can harvest energy from the ambient RF field without the need for complex localized impedance matching networks for each individual node.

In addition, as demonstrated in our study (Fig. 6 in the manuscript), rather than relying solely on the intrinsic conversion efficiency of a single active region, the output current can be linearly scaled up by employing the multi-IDT arrays (e.g., enhancing the output by approximately 113%). This provides a viable and new engineering route to compensate for the low single-element yield, allowing the system to meet the specific DC current thresholds required for our targeted bio-stimulation applications.

We believe that this transparent approach, which acknowledges the low absolute efficiency while highlighting the spatial broadcast and array scaling capabilities, provides the scientifically accurate representation of our work.

Revised:

1. "Although the proposed acoustic-driven current source demonstrates a unique rectifier-free mechanism and shows significant potential for specific bioelectronic applications, it is needed to acknowledge its current performance boundaries if compared to those using the conventional semiconductor technologies. Firstly, the RF-to-DC power conversion efficiency of the AE effect in the current single-device configuration is substantially lower than that of conventional rectification circuits[35,36]. Consequently, this technology is not intended to serve as a direct replacement for highly evolved rectifiers in scenarios which demand the maximum point-to-point power transfer efficiency. In contrast, in the ambient or broadcast RF environments, its simplified architecture allows multiple distributed AE devices to be excited simultaneously. As demonstrated in this study, multi-IDT arrays can effectively scale up the harvested current to meet practical application thresholds." (Page 21)

Comment 3. While acknowledging that transistor circuits can readily achieve nA level current sources, the authors still claim in the introduction "which makes stable, continuous operation at the nanoampere scale a significant challenge." Making nA level current sources with sustained operation is not at all challenging, especially ones with such poor conversion efficiency and output resistance.

Response:

We sincerely thank the reviewer for this important clarification. We fully agree with the assessment. Transistor-based circuits are well established in their ability to generate stable and continuous nanoampere-level current sources with a high degree of controllability and reproducibility, and such functionality does not constitute a technical challenge. Our previous statement suggesting otherwise was inaccurate and potentially misleading, especially in the context of well-established circuit design capabilities.

In response, we have **completely removed this statement from the Introduction** to avoid any incorrect implication. We also acknowledge that, given the relatively low conversion efficiency and limited output resistance of the present device, it is inappropriate to position this work in comparisons with those conventional transistor-based current sources in terms of performance metrics.

Instead, we have revised the Introduction to more clearly delineate the scope and intent of this work, as reflected in the revised manuscript (**Introduction section, Page 3**). The focus is now placed on the distinct operational paradigm of the proposed acoustoelectric approach, which enables direct generation of nanoampere-level currents under wireless excitation without relying on the conventional rectification and regulation circuitry. This distinction is presented as a difference in system architecture rather than an advantage in current generation capability.

Comment 4. The authors acknowledge that transistors can make nA scale current sources with much higher output resistance, but the claim is that these are not or cannot be wireless. To quote the response letter "In summary, while CMOS circuits can produce nanoampere currents, the acousticdriven current source offers unique advantages in terms of wireless operation, miniaturization, and energy harvesting, which are not achievable with standard transistor-based approaches." I could not disagree more. At the RF power levels in this paper, diode-based rectifiers have many orders of magnitude (~ 10^6) higher RF-to-DC conversion efficiency, meaning that a rectifier followed by a transistor current source easily realizes a much more efficient wireless current source and such current sources have been reported countless times in the literature. DOI 10.1186/s40486-017-0051-0 is a semi recent (2017) review article on RF power harvesting. Table 3

reviews rectifier technologies, where for example, http://dx.doi.org/10.1016/j.mejo.2015.11.012 achieves much higher RF-to-DC conversion efficiencies of greater than 50% across a wide range of load conditions. Again, there are many such rectifiers in the literature, some of which are listed in the review article. These rectifiers are small and implemented in transistor circuit technologies. A rectifier followed by a current source has been implemented on a single IC chip countless times to realize vastly superior wireless current sources.

Response:

We sincerely apologize for the highly inaccurate and scientifically inappropriate statements made in our previous response letter and the original manuscript regarding the presumed "unique advantages" of our device over standard transistor-based approaches.

We fully agree with your rigorous criticism and the engineering facts you have present. It is true that the modern and highly evolved diode-based and CMOS rectifiers have demonstrated outstanding RF-to-DC conversion efficiencies (easily exceeding 50%) and have been successfully implemented on miniature, single integrated circuits for fully passive wireless energy harvesting, as summarized in the literature you provided. Our previous assertion that achieving miniaturized, wireless energy harvesting was "not achievable with standard transistor-based approaches" was factually incorrect.

We deeply appreciate your correction for this point and thank for providing the crucial references (DOI: 10.1186/s40486-017-0051-0 and 10.1016/j.mejo.2015.11.012). Your feedback has effectively prevented us from inappropriately framing a preliminary physical mechanism exploration as a narrative capable of overturning traditional, highly optimized circuit designs.

Following the Editor's explicit requirement to tone down our claims and avoid downplaying alternative technologies, we have conducted a thorough modification of our manuscript. We have completely removed all the statements which attempted to claim superior engineering performance or integration advantages over existing CMOS rectification technologies.

Instead, we have fundamentally reshaped the scientific positioning of this research. Our objective is absolutely not to challenge these highly perfected RF rectification technologies in terms of conversion efficiency. Conversely, our goal is to explore and demonstrate, at a fundamental physical level, an alternative, and new physical energy conversion mechanism that is "rectifier-free."

In modern micro-fabrication systems, traditional RF energy harvesting typically relies on a cascade of components, commonly including an antenna for RF reception, an impedance matching network, nonlinear rectification devices, and filtering capacitors. The mechanism demonstrated in this work bypasses this classical electrical "rectification" step entirely. It generates DC current directly by utilizing the electric field accompanying SAWs propagating on a piezoelectric substrate to drive the

manipulation and transport of charge carriers.

While this physical conversion mechanism currently incurs a substantial efficiency issue due to limited acoustoelectric coupling strength, it remarkably demonstrates the feasibility of generating DC current in a simple thin-film system without relying on conventional rectifying semiconductor junctions. We believe this alternative pathway in physical mechanisms holds the scientific reference values for highly specific bio-interfacing explorations that are volume-constrained and require extremely low absolute power, as demonstrated by the in vivo weak current stimulation in our study.

Revised:

1. "Wireless power transfer (WPT) offers a viable pathway toward autonomous and untethered operation. In the conventional WPT systems, a stable DC output is typically obtained through a cascade of functional stages, including an antenna for RF reception, impedance matching networks, nonlinear rectification elements, and filtering components. While effective, such multi-stage architectures inevitably increase system-level complexity and introduce inherent trade-offs among device size, transmission distance, and output stability, particularly under high-loss or harsh operating environments." (Page 3)

2. "In this context, rather than extending the established semiconductor-based power conversion technologies, we explore an alternative physical mechanism based on the AE effect. In this approach, a continuous DC is generated through the interaction between SAWs and semiconductors, and such mechanism enables a simplified, rectifier-free architecture which consolidates the RF-to-DC conversion process within a single heterostructure platform. This enables a compact and battery-free solution capable of stable nanoampere-level output under wireless RF excitation, where the incident RF signal is coupled via an antenna to directly drive the device." (Page 3)

Comment 5. In wirelessly powered applications, such as the one proposed by the authors, having a high output resistance is critical for the current source and it will need to be much higher than 10 kΩ. Let's explore why. The authors state in the paper, "Overall, while the output impedance of the acoustic driven current source is lower than that of conventional voltage-controlled current sources, it remains sufficiently high for intended low-power wireless applications, particularly when the load resistance is much smaller than the source impedance (e.g., < 1 kΩ), ensuring that current errors due to loading remain below 10%." Even at the highest RF input power levels input to the SAW in this paper of 200 mW (23 dBm), the current source only produces about 20 μA. If we source 20 μA into a 1 kΩ load as the authors suggest, the voltage supplied to the load will be 20 mV, far too low of a supply voltage to operate any known transistor or semiconductor logic technology. Perhaps the

authors can cite what useful they could power at such a low voltage. Given the extremely poor RF-to-DC conversion efficiency of this device it can only power extremely low power circuits. Those circuits must have a supply volage of at least a few hundred mV which means, by definition, the load impedance must be large. Let's assume an ultralow supply voltage of 300 mV and the maximum current that can be supplied by this device of 20 µA at the ridiculously high received RF power level of 23 dBm for a wirelessly powered device. In such a case, the load impedance is 15 kΩ, above the output impedance of the current source, which means that the 300 mV won't even be achievable. If the RF input power were to drop to a still very large 10 mW (10 dBm), then the load impedance will need to increase to 150 kΩ, well above the output resistance of this current source. And the authors are claiming this current source can be used down to the nA level. I really can't see how this device can wirelessly power anything useful unless the conversion efficiency and output resistance are significantly improved.

Response:

We sincerely thank the reviewer for this insightful and constructive critique. We fully acknowledge the validity of the electrical analysis presented. We fully agree with your view that, when "powering something useful" is defined as driving silicon-based logic circuits or transistors, our device is fundamentally incapable of doing so. As you correctly calculated, the maximum voltage compliance of our device (tens to hundreds of millivolts) falls drastically short of the threshold voltage required to turn on any known semiconductor logic technology. Following your rigorous assessment and the Editor's directive to clearly state limitations, we have incorporated limitations and future perspectives into the revised manuscript and have explicitly acknowledged this inability to drive high-impedance logic circuits in the **Discussion section (Page 21)**.

However, we should clarify that the primary design intention and the actual in vivo application target of this acoustic-driven current source are not to power silicon logic chips, but rather to be applid for application of living biological tissues (as successfully demonstrated in our rat wound healing model). In the field of biomedical electrostimulation, the load characteristics and driving requirements are fundamentally different from those of semiconductor circuits.

1. Ultra-Low Load Impedance

Real living tissues, extracellular fluids, or wound exudate environments are highly conductive aqueous solutions rich in electrolytes. Their localized equivalent tissue impedance is typically very low, generally ranging from a few hundred ohms to less than 2 kΩ. In fact, in the design and testing of implantable biomedical stimulators (including those based on advanced CMOS ICs), an equivalent load resistor of 1 kΩ is widely used to model body tissues or agar gel phantoms, as demonstrated in previous studies (DOI: 10.1109/ISCAS56072.2025.11043922).

2. Therapeutic Efficacy Depends on Microcurrent Density, Not Absolute High Voltage

In clinical microcurrent therapy and studies promoting cellular galvanotaxis, the recognized effective therapeutic window for physiological modulation is typically in the range of microamperes (e.g., 10 μA to 20 μA), without the need for high driving voltages.

As per your exact calculation, driving a 20 μA current into a 1 kΩ biological load results in a voltage drop of exactly 20 mV across the tissue. Although 20 mV may be considered useless noise for a silicon circuit, it is highly useful and appropriate for a biological entity. Because a typical cell size is on the micrometer scale, on the order of 10 μm, a 20 mV transcellular voltage drop is sufficient to establish a remarkably strong localized electric field gradient of approximately 20 V/cm within the cellular microenvironment. Such field strengths are entirely sufficient to induce cell membrane depolarization, activate ion channels, and successfully promote wound healing. For eexample, A very recent state-of-the-art study published in *Nature Electronics* (DOI: 10.1038/s41928-024-01138-8) perfectly corroborates this, showing that a micro-galvanic cell generating extremely low voltages in the sub-volt to millivolt range can provide continuous microcurrent stimulation to intestinal epithelial tissues and substantially accelerate wound healing[2].

Conversely, if one wants to apply the hundreds of millivolts or volt-level potentials (required by silicon logic) directly across such ultra-low impedance biological tissues, it could risk irreversible tissue burning, electrolysis, and severe electrochemical toxicity.

Therefore, our device provides a "self-limiting low-voltage, safe constant microcurrent," which perfectly matches the safety and stimulation requirements of low-impedance biological loads. We are immensely grateful for your great comments, and now we have thoroughly and transparently incorporated this discussion into the revised Discussion section, ensuring that future readers can appreciate the specific utility of this device.

Revised:

1. "Secondly, the current device exhibits a moderate output impedance and a voltage compliance in the range of tens to hundreds of millivolts. While these characteristics may present challenges for directly driving standard high-impedance silicon logic gates without supplementary voltage boosting, they are highly suitable for a variety of low-voltage, low-impedance micro-loads. Specifically, the device is well-matched for driving biological interfaces[37,38], where continuous nA-to-μA level currents are functionally effective. Future optimizations focusing on enhancing the electromechanical coupling coefficient and semiconductor carrier mobility will be pursued to further broaden the electrical output capabilities of this platform." (Page 21)

Reviewer: 3

In my opinion, the authors have successfully addressed all technical comments from both Reviewers by this revision. I recommend publication of this work in *Nature Communications*.

Response:

We sincerely thank you for your rigorous review, insightful questions, and your final recommendation for publication. Your profound questions regarding the transient response in the previous round significantly elevated the physical depth of our manuscript.

Reviewer: 5

I co-reviewed this manuscript with one of the reviewers who provided the listed reports. This is part of the *Nature Communications* initiative to facilitate training in peer review and to provide appropriate recognition for Early Career Researchers who co-review manuscripts.

Response:

We deeply appreciate your time and effort in co-reviewing our manuscript. Your contributions have been invaluable in improving the quality of our work.

Reviewer: 6

The authors present a variable amplitude current source based on the acoustoelectric (AE) effect. The current source takes in an RF input power at the 430 MHz input frequency of a Rayleigh SAW IDT and outputs a DC current whose amplitude is proportional to the RF input power.

I thank the authors for more directly addressing my review of their paper, and specifically the limitations of the reported technology, in this round of revision. I have grave reservations about the impact of this paper and its appropriateness for publication in *Nature Communications*. If it is to be published in such a widely read journal that targets very high impact scientific findings to a broad audience, in my view the context provided by the authors needs a presentation where a non-expert can clearly see the significant limitations of the work. My comments below should not be interpreted as an endorsement to publish this paper in *Nature Communications*, it should be taken as feedback to further improve the paper should the editors ultimately decide to publish it.

Response:

We sincerely thank the reviewer for this thoughtful and constructive assessment. We agree that, for the broad readership of *Nature Communications*, the proposed technology should be presented within a clear and objective engineering context, including its current practical limitations. Following the reviewer's suggestions, we have substantially revised the manuscript to provide a more balanced and transparent discussion of the work. In particular, we have clarified the positioning of the proposed acoustoelectric current source relative to well-established RF-to-DC rectification technologies, expanded the discussion of its current limitations and future development, and added additional quantitative analyses and comparisons where appropriate. We believe these revisions significantly improve the clarity, transparency, and overall balance of the manuscript.

Comment 1. It is well understood in energy harvesting that highly efficient RF-to-DC conversion is achievable at the large input powers reported in this paper. It is also quite common to show a plot of P_{out} (DC) on the y-axis vs. P_{in} (RF) on the x-axis. I understand the authors are presenting a current source, but ultimately the current source is to deliver wireless power, and it does so with a very poor efficiency (0.0001% and output resistance of 10 kOhm) whose value is not explicitly reported in the paper. The authors should include a plot of P_{out} (DC) vs. P_{in} (RF) of their current source (simply match it for maximum power transfer) and on the same graph include points or curves from state-of-the art literature. The Nature Comms readers should be able to clearly see this technology in its current form is 6 orders of magnitude lower in conversion efficiency when compared to the state-of-the-art and will require significant advancements before it exhibits practical utility. I see no path where increases in electromechanical coupling (at most 10x for known piezo materials and modes) and arraying close a gap of 6 orders of magnitude in performance.

Response:

We thank the reviewer for this critical and constructive comment. We fully agree that a transparent, quantitative comparison with state-of-the-art RF-to-DC rectifiers is essential for readers to properly assess the current limitations of this technology.

Following the reviewer's suggestion, we have added **Supplementary Note X** and **Supplementary Fig. 14** in the Supplementary Information, plotting P_{out} versus P_{in} for our device against data from representative state-of-the-art RF rectifiers.

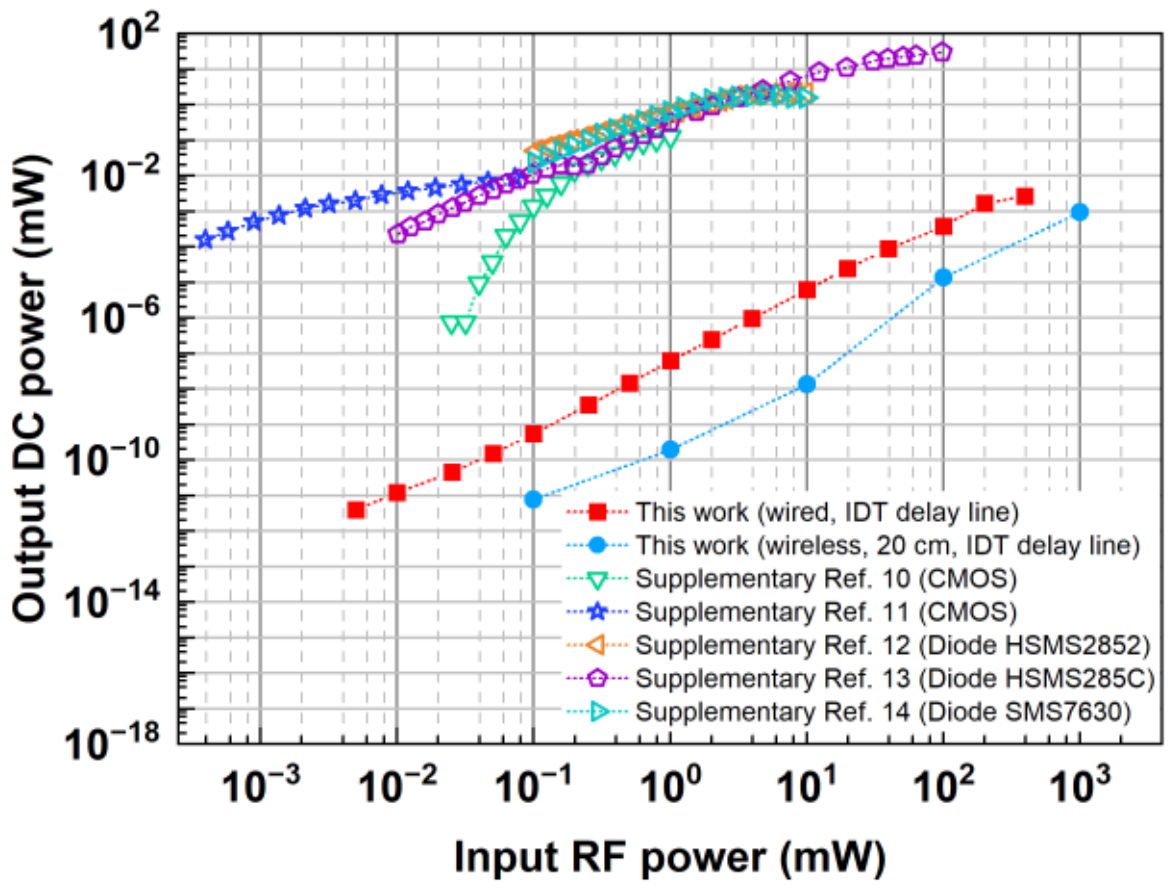

Supplementary Fig. 14. Output DC power P_{out} versus input RF power P_{in}. The acoustic-driven current source (wired operation, and wireless operation at a transmission distance of 20 cm, based on the SAW delay line configuration) is compared with state-of-the-art RF-to-DC rectifiers[10–14]. These include CMOS implementations, such as those by Chouhan[10] and Wang[11]. In addition, discrete Schottky-diode-based implementations include those by Abdelaal[12], Tariq[13], and Saini[14]. For the acoustic-driven current source, P_{out} is calculated under the maximum power transfer condition (R_L=R_{out}) with R_{out}≈10.5 kΩ. Source data are provided as a Source Data file.

As shown in **Supplementary Fig. 14**, we have explicitly reported this efficiency value and its performance gap in the manuscript (Discussion section) and included the quantitative comparison in the Supplementary Information, as the reviewer recommended. Nevertheless, we acknowledge that substantial improvements in materials and device architecture are still required before this acoustoelectric approach can be translated into practical wireless power delivery applications.

We believe these additions provide readers with a clear and honest assessment of the technology's current performance boundaries while preserving the novelty of the acoustoelectric approach.

Supplementary References:

10. Chouhan, S. S., Nurmi, M., & Halonen, K. Efficiency enhanced voltage multiplier circuit for

RF energy harvesting. Microelectronics journal 48, 95-102 (2016).

11. Wang, G., Li, X. & Chen, Z. A 2.4-GHz −33-dBm sensitivity battery-free RF energy harvesting system with 17-dB input power range. *IEEE Trans. Circuits Syst. II Exp. Briefs* **73**, 1120–1124 (2026).
12. Abdelaal, M., Elsayed, M., & Mansour, I. Design of High-Efficiency RF Energy Harvesting Rectifier Circuit. In *2026 43rd National Radio Science Conference (NRSC)*, IEEE (2026).
13. Tariq, B. et al. Design and analysis of a dual-port multiband RF-to-DC converter for IoT energy harvesting applications. *PLoS One* **20**, e0321729 (2025).
14. Saini, P., Malhotra, S. & Gupta, L. A compact and wideband rectifier for RF energy harvesting with robust performance across varying input power and load range. *Phys. Scr.* **100**, 125026 (2025).

Revised:

1. "Although the proposed acoustic-driven current source demonstrates a rectifier-free mechanism and shows significant potential for specific bioelectronic applications, it is needed to acknowledge its current performance boundaries if compared to those using the conventional semiconductor technologies. Firstly, the low RF-to-DC power conversion efficiency of the AE effect in the current single-device configuration is lower than that of conventional rectification circuits[32,33]. As shown in Supplementary Note X, which compares output DC power and input RF power, the observed efficiency is orders of magnitude lower than that typically reported for rectification circuits." (Manuscript, Page 15)

Comment 2. In response to my prior feedback the authors point to the fact that traditional WPT systems include a cascade of stages: "Wireless power transfer (WPT) offers a viable pathway toward autonomous and untethered operation. In the conventional WPT systems, a stable DC output is typically obtained through a cascade of functional stages, including an antenna for RF reception, impedance matching networks, nonlinear rectification elements, and filtering components. While effective, such multi-stage architectures inevitably increase system-level complexity and introduce inherent trade-offs among device size, transmission distance, and output stability, particularly under high-loss or harsh operating environments." (Page 3). The authors should acknowledge that at the input powers where their technology produces useful output currents of more than 1 nA, that the traditional WPT systems become significantly simplified. No impedance matching circuits will be required at these input powers since the voltages are well in excess of the diode turn on voltage. As such, rectifiers can be extremely small (simply 4 tiny diodes on a chip) and realized in extremely inexpensive Si technology. The only possible advantage I can see is the removal of the smoothing capacitor, as both traditional and acousto-electric WPT will require an antenna.

Response:

We thank the reviewer for this insightful comment regarding the operating regime of conventional rectification circuits. We fully agree that, at sufficiently high RF input power levels corresponding to our experimental conditions (where output currents exceed ~1 nA), the voltage across diode-based rectifiers is typically well in excess of the turn-on threshold, enabling highly efficient operation. In this regime, rectification circuits can be implemented in extremely compact integrated implementations and often do not require external impedance matching networks.

In response to this comment, we have revised the manuscript to accurately reflect the state of the art in integrated rectifier design. In particular, we have removed previous statements that could be interpreted as overstating the system-level complexity or trade-offs of conventional WPT approaches.

In the revised Introduction, we now present a more balanced description. We acknowledge that while conventional RF-to-DC conversion involves discrete functional stages (RF reception, rectification, and filtering), mature integrated rectifiers can achieve highly compact implementation and excellent efficiency under these operating conditions. We have consequently refocused the narrative entirely on the fundamental physical distinction of the AE effect. Our goal is solely to present the AE mechanism as an alternative physical pathway for direct RF-to-DC conversion, without implying any superiority in circuit simplicity or integration level.

Revised:

1. "At sufficiently high RF input power levels, the conventional diode- or transistor-based rectification circuits can be substantially simplified, and complex impedance matching networks are often no longer required. Such rectifiers can therefore be realized in highly compact integrated forms using mature silicon technologies.

   In this context, rather than pursuing conventional semiconductor-based RF-to-DC conversion technologies, we explore an alternative physical mechanism based on the acoustoelectric (AE) effect. In this approach, a continuous DC current is generated through the interaction between surface acoustic waves (SAWs) and semiconductors. This mechanism enables RF-to-DC conversion without relying on discrete semiconductor rectification elements, conceptually consolidating the energy conversion process within a single heterostructure platform." (Manuscript, Page 3)

Comment 3. The authors provide a biomedical interface application where their current source would have functional utility, and generally I agree the DC currents produced, and the frequency response demonstrated would be applicable to biological stimulation. However, the poor efficiency

is also quite limiting in this application. The IEEE Standard for Safety Levels with Respect to Human Exposure to Radio Frequency Electromagnetic Fields, 3 kHz to 300 GHz", IEEE C95.1-1991 limits the maximum continuous power density on the body at 430 MHz (the frequency of the reported device) to approximately 0.3 mW/cm^2. According to the authors' response letter, "In clinical microcurrent therapy and studies promoting cellular galvanotaxis, the recognized effective therapeutic window for physiological modulation is typically in the range of microamperes (e.g., 10 μA to 20 μA), without the need for high driving voltages." To produce just 1 μA of stimulation current, the reported device requires 10 mW of received power. This will require at a minimum a 33 cm^2 receive antenna to remain within the safe exposure limits and this analysis doesn't even account for any loss between the transmit and receive antennas in the proposed biomedical application. If 10 μA of current is needed as identified by the authors, 100 mW of received power will be needed requiring a 333 cm^2 receive antenna (18 x 18 cm!), again with no loss between the transmit and receive antennas which will make this situation much worse. A simple inductive loop, diode, and smoothing capacitor could do this over a 1 cm distance while occupying less than 1 mm on a side. Go to 1 cm on a side, and 5 cm distances with 100 mW level power delivery is commonly reported, from which any biologically relevant current can be delivered. The proposed biomedical application simply highlights the premium placed on efficiency in WPT applications and why no device that is 1 million times less efficient than the state-of-the-art can claim to simply the system. I go back to another comment from the authors about traditional WPT "While effective, such multi-stage architectures inevitably increase system-level complexity and introduce inherent trade-offs among device size, transmission distance, and output stability, particularly under high-loss or harsh operating environments." The poor efficiency of the reported device greatly exasperates the tradeoffs between size and transmission distance. It cannot be reasonably claimed via a quantitative analysis that it will simplify these tradeoffs.

Response:

We sincerely thank the reviewer for this insightful quantitative analysis based on the IEEE C95.1 safety standard. We fully agree that the current conversion efficiency substantially limits the practical implementation of the proposed biomedical application under realistic RF exposure constraints.

Following the reviewer's excellent suggestion, we have incorporated this exact quantitative analysis into the revised manuscript. We explicitly discuss the resulting trade-offs among power transfer efficiency, required antenna size, and transmission distance under standard safety regulations. We have accordingly revised the text to objectively clarify that, in its current form, the proposed device should not be regarded as a practical wireless power source for deep-tissue biomedical implants. By transparently presenting these constraints, we ensure that readers have a realistic understanding of the technology's current engineering boundaries.

Revised:

1. "Secondly, the low conversion efficiency of the developed device presents its constraints for practical implementation in clinical biomedical interfaces when realistic radiofrequency exposure regulations are considered. According to the IEEE Standard for Safety Levels (IEEE C95.1-1991), the maximum safe continuous power density on the human body at 430 MHz is limited to approximately 0.3 mW/cm^2. If the device were required to operate within these safe exposure limits to harvest RF power, the required receiving antenna area would be impractically large, even if the therapeutic current were limited to just 10 μA. This imposes a constraint on the trade-off between device miniaturization and transmission distance. Finally, the current device exhibits a moderate output impedance and a voltage compliance in the range of tens to hundreds of millivolts. While these characteristics may present challenges for directly driving standard high-impedance silicon logic gates without supplementary voltage boosting, they are applicable for a variety of low-voltage and low-impedance micro-loads. Specifically, the device is well-matched for driving biological interfaces[34,35], where continuous nA-to-μA level currents are functionally effective. Consequently, the present work should primarily be regarded as a fundamental demonstration of an alternative acoustoelectric mechanism for direct RF-to-DC conversion. Future advances in material properties, including enhancing the electromechanical coupling coefficient and semiconductor's carrier mobility, together with improvements in device architecture and system-level design, will be required before this approach can be considered for broader wireless power transfer applications." (Manuscript, Page 15)

Statement on Manuscript Refinements

In addition to addressing the reviewers' comments, we have taken this opportunity to further improve the precision and clarity of our data presentation. Specifically, we have revised the presentation formats of Figures 5c and 6f to provide a more transparent and objective representation of the experimentally observed results.

- For Fig. 5c, we revised only the vertical axis label from "current attenuation" to "ratio of wireless current to wired current ($I_{wireless}/I_{wired}$)" to provide a more direct and accurate description of the relationship between wireless and wired current outputs.
- For Figure 6f, we revised the data presentation from the original current-power response to the output current ratio (I_{SPUDT}/I_{IDT}) to provide a clearer representation of the relative current response between the SPUDT and IDT structures. The SPUDT-based device exhibits higher output current than the conventional IDT structure. In addition, the I_{SPUDT}/I_{IDT} ratio was experimentally observed to increase with increasing input power. This experimentally verified phenomenon is presented more explicitly to provide a clearer understanding of the device performance. Although the underlying mechanism responsible for this variation remains to be clarified, investigating its origin will be an important point for future research.

We wish to clarify that these revisions are strictly limited to the data visualization format; no original experimental data has been altered, and no new data has been added. These changes were made solely to improve the objectivity, transparency, and readability of the data presentation, ensuring that the experimental observations are communicated in a clear and unbiased manner.
